# Global, regional, and national burden of cancers attributable to tobacco smoking in 204 countries and territories, 1990–2019

**DOI:** 10.1002/cam4.4647

**Published:** 2022-05-27

**Authors:** Saeid Safiri, Seyed Aria Nejadghaderi, Morteza Abdollahi, Kristin Carson‐Chahhoud, Jay S. Kaufman, Nicola Luigi Bragazzi, Maziar Moradi‐Lakeh, Mohammad Ali Mansournia, Mark J. M. Sullman, Amir Almasi‐Hashiani, Ali Taghizadieh, Gary S. Collins, Ali‐Asghar Kolahi

**Affiliations:** ^1^ Aging Research Institute Tabriz University of Medical Sciences Tabriz Iran; ^2^ Social Determinants of Health Research Center, Department of Community Medicine, Faculty of Medicine Tabriz University of Medical Sciences Tabriz Iran; ^3^ Systematic Review and Meta‐analysis Expert Group (SRMEG) Universal Scientific Education and Research Network (USERN) Tehran Iran; ^4^ Social Determinants of Health Research Center Shahid Beheshti University of Medical Sciences Tehran Iran; ^5^ Australian Centre for Precision Health University of South Australia Adelaide South Australia Australia; ^6^ School of Medicine University of Adelaide Adelaide South Australia Australia; ^7^ Department of Epidemiology, Biostatistics and Occupational Health, Faculty of Medicine McGill University Quebec Canada; ^8^ Centre for Disease Modelling York University Toronto Ontario Canada; ^9^ Preventive Medicine and Public Health Research Center Iran University of Medical Sciences Tehran Iran; ^10^ Department of Epidemiology and Biostatistics, School of Public Health Tehran University of Medical Sciences Tehran Iran; ^11^ Department of Life and Health Sciences University of Nicosia Nicosia Cyprus; ^12^ Department of Social Sciences University of Nicosia Nicosia Cyprus; ^13^ Department of Epidemiology, School of Health Arak University of Medical Sciences Arak Iran; ^14^ Tuberculosis and Lung Diseases Research Center Tabriz University of Medical Sciences Tabriz Iran; ^15^ Centre for Statistics in Medicine, NDORMS, Botnar Research Centre University of Oxford Oxford UK; ^16^ NIHR Oxford Biomedical Research Centre Oxford University Hospitals NHS Foundation Trust Oxford UK

**Keywords:** cancer, death, disability‐adjusted life year, global burden of disease, smoking

## Abstract

**Background:**

Cancers are leading causes of mortality and morbidity, with smoking being recognized as a significant risk factor for many types of cancer. We aimed to report the cancer burden attributable to tobacco smoking by sex, age, socio‐demographic index (SDI), and cancer type in 204 countries and territories from 1990 to 2019.

**Methods:**

The burden of cancers attributable to smoking was reported between 1990 and 2019, based upon the Comparative Risk Assessment approach used in the Global Burden of Disease (GBD) study 2019.

**Results:**

Globally, in 2019 there were an estimated 2.5 million cancer‐related deaths (95% UI: 2.3 to 2.7) and 56.4 million DALYs (51.3 to 61.7) attributable to smoking. The global age‐standardized death and DALY rates of cancers attributable to smoking per 100,000 decreased by 23.0% (−29.5 to −15.8) and 28.6% (−35.1 to −21.5), respectively, over the period 1990–2019. Central Europe (50.4 [44.4 to 57.6]) and Western Sub‐Saharan Africa (6.7 [5.7 to 8.0]) had the highest and lowest age‐standardized death rates, respectively, for cancers attributable to smoking. In 2019, the age‐standardized DALY rate of cancers attributable to smoking was highest in Greenland (2224.0 [1804.5 to 2678.8]) and lowest in Ethiopia (72.2 [51.2 to 98.0]). Also in 2019, the global number of DALYs was highest in the 65–69 age group and there was a positive association between SDI and the age‐standardized DALY rate.

**Conclusions:**

The results of this study clearly illustrate that renewed efforts are required to increase utilization of evidence‐based smoking cessation support in order to reduce the burden of smoking‐related diseases.

## Graphical abstract

We show that almost one in every four deaths, and one in every five DALYs, due to cancer was as a result of exposure to smoking. In 2019, the global number of DALYs was highest in the 65‐69 age group and there was a positive association between SDI and the age‐standardized DALY rate.

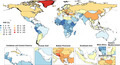

## 1 INTRODUCTION

Cancers are leading causes of mortality and morbidity across the world, with substantial increases being observed in the death rate (44.5%) and the disability‐adjusted life years (DALYs) (56.2%) from 1990 to 2019.^1^, ^2^ Moreover, in 2019 cancers were found to account for 3943.1 (95% uncertainty interval [UI]: 3342.2–4712.4) incident cases and 130.3 (121.7–137.8) deaths per 100,000 population.^1^, ^2^

The World Health Organization (WHO) has proposed tobacco smoking and alcohol use, unhealthy diet, insufficient physical activity, air pollution, and even some chronic viral infections as risk factors for the development of cancer.^3^ Smoking is a risk factor for several communicable and noncommunicable diseases. Smoking may also interact with some drug pharmacokinetics and can also affect treatment outcomes,^4^ including cancer treatments.^5^ Furthermore, smoking can be a risk and prognostic factor for different types of cancers, such as lung, and digestive and urinary tract cancers.^6^

In 2019, 7.4 trillion (7.1–7.7) cigarette‐equivalents of tobacco were used by 1.1 billion (1.1–1.2) current smokers across the world. The age‐standardized prevalence of current tobacco smoking in males was higher than among females aged 15 years and older in 2019 (32.7% vs. 6.6%). Although a decreasing trend for the prevalence of smoking has been observed in females and males since 1990 (−37.7% and −27.5%, respectively), the prevalence and the burden of tobacco smoking is still considerable.^7^ Moreover, there is concern by many tobacco control and public health experts that electronic cigarettes (e‐cigarettes) are acting as a gateway to the initiation of new smokers,^8^, ^9^, ^10^ whereas e‐cigarettes have also been suggested as a harm‐reduction policy.^11^

In 2019, the overall age‐standardized prevalence of tobacco smoking was 24.0% (22.8%–25.4%), but it is noteworthy that this is a very diverse behavior.^12^ In addition, smoking was one of the largest risk factors in all age groups worldwide and was responsible for 7.9% (7.2%–8.6%) of all‐cause DALYs in 2019.^12^ Since the incidence and death rates of cancers are substantial and smoking is a major risk factor for cancers,^6^, ^13^ estimating the burden of cancers attributable to smoking is an important public health issue.

The association between smoking and different types of cancers have been evaluated in previous studies,^14^, ^15^, ^16^ although there is a lack of research investigating these associations by location and socio‐demographic index (SDI). Although the burden of different cancers attributable to smoking has previously been evaluated at the regional and national levels,^17^, ^18^, ^19^, ^20^, ^21^ there is no published paper to report the burden of cancers attributable to smoking by cancer type, age, sex, and SDI. Therefore, using data from the GBD 2019 study, we aimed to report the burden of cancers attributable to smoking by age, sex, SDI, and cancer type in 204 countries and territories from 1990 to 2019.

## 2 METHODS

### 2.1 Overview

The GBD project, which estimates the burden of diseases and injuries across the world, is administered by the Institute for Health Metrics and Evaluation (IHME). In GBD 2019, the most recent version of the study, 369 diseases and injuries and 87 risk factors were investigated from 1990 to 2019 at the global, regional, and national levels.^2^, ^12^, ^22^ The GBD 2019 methodology for estimating the burden of diseases, injuries and risk factors have been reported in detail elsewhere.^2^, ^12^, ^22^ Complete information on nonfatal estimates can be found at https://vizhub.healthdata.org/gbd‐compare/ and http://ghdx.healthdata.org/gbd‐results‐tool.

### 2.2 Case definition and data inputs

Nationally representative household surveys were used to estimate the prevalence of current and former smokers.^7^ According to the GBD project, current smokers are individuals who currently use any smoked tobacco product on a daily or occasional basis. Former smokers include as individuals who quit using all smoked tobacco products for at least 6 months, where possible, or according to the definition used by the survey. All other data points were adjusted to be consistent with either of these definitions. There were several sources which reported information using more than one case definition and this information was used to develop the adjustment coefficient to transform alternative case definitions to the GBD case definition.

Primary data were extracted from individual level microdata and survey report tabulations. Data on current, former, and/or lifetime smoked tobacco use were reported as any combination of frequency of use (daily, occasional, and unspecified, which includes both daily and occasional smokers) and type of smoked tobacco used (all smoked tobacco, cigarettes, hookah, and other smoked tobacco products such as cigars or pipes). For microdata, relevant demographic information was extracted, including age, sex, location, and year, as well as survey metadata, including survey weights, primary sampling units, and strata. This information enabled the tabulation of individual‐level data in the standard GBD 5‐year age‐sex groups (from age 30 to 95^+^) and uncertainty estimates. For survey report tabulations, data in the smallest age‐sex groups have been provided. Finally, data on smoking prevalence was found for 3439 data sources from 201 countries. More detailed information on the data sources are available here: http://ghdx.healthdata.org/gbd‐2019/data‐input‐sources.

### 2.3 Smoking prevalence and exposure distribution

The prevalence of smoking (former/current) was modeled using spatio‐temporal Guassian process regression (ST‐GPR) models. Further details on the ST‐GPR method are available in the appendix of a previous publication.^12^ Prevalence estimates of current and former tobacco smoking define the full population at risk, but the risk of disease varies substantially within these groups, according to the smoking intensity and time since cessation. These risk differences were incorporated in the estimation framework by modeling continuous exposure distributions among current and former smokers. Cigarettes per smoker per day and pack‐years were used to estimate exposure among current smokers. Pack‐years includes aspects of both duration and quantity, with one pack‐year representing the equivalent of smoking one packet of cigarettes (assuming a 20‐cigarette pack) per day for 1 year. However, as the pack‐years indicator combines duration and intensity into a single dimension, one pack‐year of exposure could mean smoking 40 cigarettes per day for 6 months or smoking 10 cigarettes a day for 2 years.

These indicators were produced using individual‐simulated smoking histories, which were estimated using distributions of the age of initiation and the quantity smoked. The simulation used data from cross‐sectional surveys which measured these indicators, modeled using ST‐GPR at the mean level for all locations, years, ages, and sexes.^7^ The estimated number of cigarettes per smoker per day was re‐scaled to an envelope of cigarette consumption. The pack‐years of exposure was estimated by summing samples from age‐ and time‐specific distributions of cigarettes per smoker for each birth cohort to capture trends associated with both age and time, as well as avoiding the common assumption that the quantity an individual currently smokes is the amount they have smoked since they started smoking. All distributions were age‐, sex‐, and region‐specific ensemble distributions, which produced more accurate results than any single distribution.

The years since cessation was used to estimate exposure among former smokers. ST‐GPR was again used to model the mean age of cessation using data on the age of cessation from cross‐sectional surveys. These estimates were used to produce ensemble distributions of years since cessation for every location, year, age group, and sex.

### 2.4 Data on estimated relative risk

In order to systematically examine the epidemiologic evidence supporting the association between smoking and various cancers, the Bradford Hill^23^ and World Cancer Research Fund grading^24^ criteria were used. In total, 16 cancers were found to be at least partially attributable to smoking, including bladder cancer^25^; breast cancer^26^; cervical cancer^27^; colorectal cancer^28^; esophageal cancer^28^; kidney cancer^27^; larynx cancer^29^; leukemia^30^; lip and oral cavity cancer^29^; liver cancer^28^; nasopharynx cancer^31^; pancreatic cancer^26^; prostate cancer^32^; stomach cancer^26^; tracheal, bronchus and lung cancer^27^; and other pharynx cancers.^12^

The relative risks were estimated using the number of cigarettes per smoker per day, pack‐years, and years since cessation from cohort and case–control studies. A Bayesian meta‐regression model used this information to produce nonlinear dose–response curves. Age‐specific risk curves were also produced for outcomes with significant differences in effect sizes by sex or age. Furthermore, the risk curves of former smokers, compared with those who never smoked, were estimated, taking into consideration the rate of risk reduction among former smokers (from cohort and case–control studies) and the cumulative exposure among former smokers within each age, sex, location, and year group.

### 2.5 Estimation of the proportion of cancers attributable to smoking

The population‐attributable fraction (PAF) by country, age, sex, and year were calculated to estimate the burden of cancers attributable to smoking using the following formula:

$$\mathrm{PAF}=\frac{p\left(n\right)+p\left(f\right)\int \exp\left(x\right)*\mathrm{rr}\left(x\right)+p\left(c\right)\int \exp\left(y\right)*\mathrm{rr}\left(y\right)-1}{p\left(n\right)+p\left(f\right)\int \exp\left(x\right)*\mathrm{rr}\left(x\right)+p\left(c\right)\int \exp\left(y\right)*\mathrm{rr}\left(y\right)}$$

Where *p*(*n*) is the prevalence of those who never smoked, *p*(*f*) is the prevalence of former smokers, *p*(*c*) is the prevalence of current smokers, exp(*x*) is the distribution of years since cessation among former smokers, *rr*(*x*) is the relative risk for years since cessation, exp(*y*) is a distribution of pack‐years, and *rr*(*y*) is the relative risk for pack‐years.

The smoking‐related deaths and DALYs from cancer were calculated for each country, age, sex, year, by multiplying the PAF by the total number of deaths or DALYs estimated in GBD 2019 for each country, age, sex, year, and type of cancer. The methods for calculating total deaths and DALYs due to cancers are reported elsewhere.^2^ All estimates were reported as counts, proportions (PAFs), and age‐standardized rates per 100,000, along with their 95% uncertainty intervals (UIs). The association between SDI and the burden of cancers attributable to smoking was investigated at both the regional and national levels. SDI is a composite measure of lag‐distributed income per capita, average years of schooling for the population older than 15 years of age, and total fertility rate under the age of 25. The SDI ranges from 0 (least developed) to 1 (most developed).

## 3 RESULTS

### 3.1 Global level

In 2019, there were an estimated 2.5 million cancer deaths (95% UI: 2.3 to 2.7) attributable to smoking globally, representing 24.7% (23.5 to 26.1) of all cancer‐related deaths (Table 1), with 2.0 (1.8 to 2.2) million of these deaths being among males and 461.7 (414.1 to 503.8) thousand females (Table S1). The age‐standardized death rate of cancers attributable to smoking in 2019 (30.6 [28.0 to 33.3] per 100,000) was 23.0% (−29.5 to −15.8) lower than the 1990 level (39.8 [37.3 to 42.1]) (Table 1). In addition, in 2019 smoking caused 56.4 million DALYs (51.3 to 61.7), representing 22.5% (21.2 to 23.8) of all cancer‐related DALYs, with 46.7 (42.1 to 51.7) million DALYs in men and 9.7 (8.8 to 10.7) million DALYs in women (Table S2). Between 1990 and 2019, the age‐standardized DALY rate of cancers attributable to smoking (per 100,000) decreased from 948.9 (888.1 to 1009.1) to 677.3 (616.4 to 740.3), a relative decrease of 28.6% (−35.1 to −21.5) from the 1990 level (Table 1).

**TABLE 1:** Cancer deaths and DALYs attributable to smoking in 2019, by GBD region

	Deaths (95% UI)				DALY (95% UI)			
	Counts (2019)	PAF (2019)	ASRs (2019)	% change in ASRs 1990–2019	Counts (2019)	PAF (2019)	ASRs (2019)	% change in ASRs 1990–2019
Global	2,493,026 (2,275,657, 2,716,575)	24.7 (23.5, 26.1)	30.6 (28, 33.3)	−23 (−29.5, −15.8)	56,446,919 (51,304,882, 61,747,452)	22.5 (21.2, 23.8)	677.3 (616.4, 740.3)	−28.6 (−35.1, −21.5)
High‐income Asia Pacific	135,234 (119,923, 146,880)	24.4 (22.9, 26.1)	28.3 (25.5, 30.4)	−37.2 (−40.8, −34.1)	2,429,548 (2,208,075, 2,606,176)	24.6 (23.2, 26.1)	585.9 (538.4, 626.8)	−41.8 (−44.7, −39.1)
High‐income North America	256,115 (237,465, 272,442)	29.5 (28.1, 31)	40 (37.3, 42.5)	−33.2 (−35.8, −30.3)	5,347,818 (5,042,527, 5,634,478)	28.8 (27.5, 30.1)	874.8 (826, 919.5)	−39.9 (−42, −37.5)
Western Europe	332,759 (307,650, 354,004)	25.8 (24.6, 27.2)	36.8 (34.5, 38.8)	−29.5 (−31.7, −27.3)	6,785,694 (6,407,173, 7,155,155)	27.2 (26, 28.5)	834.1 (791.3, 876.4)	−33.2 (−35.2, −31.1)
Australasia	11,523 (10,483, 12,420)	17.9 (16.9, 19)	23.2 (21.1, 24.8)	−48.2 (−51.1, −45.6)	244,642 (226,166, 262,136)	18.5 (17.4, 19.6)	525.2 (487.9, 563.9)	−50.4 (−53, −48)
Andean Latin America	4229 (3342, 5216)	6.5 (5.7, 7.3)	7.8 (6.2, 9.6)	−33.6 (−45.7, −19.7)	90,847 (70,790, 113,852)	5.5 (4.8, 6.3)	162.7 (126.9, 203.7)	−37.9 (−49.6, −23.7)
Tropical Latin America	53,293 (49,144, 57,412)	19.6 (18.4, 20.8)	22.2 (20.4, 23.9)	−41.7 (−45, −37.9)	1,255,064 (1,156,179, 1,350,816)	17.9 (16.8, 19.1)	506.9 (467.2, 545.4)	−44.5 (−47.7, −41)
Central Latin America	25,489 (21,490, 30,272)	11 (10, 11.9)	11.1 (9.4, 13.2)	−47.3 (−54.9, −38.4)	566,546 (468,545, 680,541)	9.3 (8.3, 10.2)	239.1 (198.6, 286.6)	−49.2 (−57.1, −40)
Southern Latin America	27,249 (25,382, 29,197)	21.5 (20.1, 22.8)	32.6 (30.4, 34.9)	−29.2 (−32.7, −25.8)	631,244 (586,495, 676,360)	21.9 (20.6, 23.3)	772.3 (717.8, 827.9)	−33.7 (−37.2, −30.5)
Caribbean	13,805 (11,718, 16,123)	19.8 (18.1, 21.5)	26.6 (22.6, 31.1)	−9.9 (−23, 4.2)	309,857 (260,087, 365,171)	18 (16.2, 19.7)	595.5 (500, 701.6)	−11.5 (−24.9, 3.2)
Central Europe	107,219 (94,339, 122,440)	31 (29.6, 32.6)	50.4 (44.4, 57.6)	−9.5 (−20.5, 1.9)	2,541,463 (2,230,735, 2,914,210)	32.4 (31.1, 33.8)	1261.1 (1103.2, 1447.4)	−15 (−25.8, −3.4)
Eastern Europe	116,035 (100,998, 131,039)	26.4 (24.3, 28.5)	33.6 (29.2, 37.9)	−23.5 (−32.7, −13.5)	3,007,670 (2,624,733, 3,415,500)	26.8 (24.8, 28.9)	894.4 (780, 1015.5)	−27.4 (−36.2, −17.8)
Central Asia	18,262 (16,349, 20,377)	20.6 (19.4, 21.8)	24.9 (22.5, 27.6)	−31.3 (−37.7, −23.7)	495,771 (441,353, 556,311)	18.4 (17.2, 19.6)	611.1 (545.9, 682.8)	−39 (−45, −31.8)
North Africa and Middle East	91,978 (81,237, 104,314)	21.9 (20.6, 23.2)	22.4 (19.8, 25.4)	−9.2 (−22.3, 8.7)	2,336,468 (2,053,920, 2,660,607)	19 (17.7, 20.4)	515.6 (454.9, 585.4)	−14.1 (−27, 3.5)
South Asia	189,502 (162,298, 220,845)	15.3 (13.7, 16.9)	14 (12.1, 16.3)	−23.3 (−35.5, −7)	4,759,026 (4,062,951, 5,602,234)	12.8 (11.4, 14.3)	326.4 (279.3, 382.2)	−23.8 (−35.9, −7.9)
Southeast Asia	140,857 (122,852, 162,001)	21.2 (19.5, 22.9)	24.4 (21.3, 28.1)	−6.5 (−19.6, 9)	3,469,585 (3,009,633, 4,007,278)	18.3 (16.7, 19.9)	546.4 (475.7, 628.9)	−11.2 (−24.2, 4)
East Asia	924,474 (758,790, 1,114,252)	32.8 (30, 35.5)	45 (37.1, 54)	−3.4 (−23.9, 23.2)	21,001,451 (16,984,703, 25,387,046)	30 (27.2, 32.6)	972.6 (790.5, 1171.3)	−12.7 (−32.4, 14.3)
Oceania	1413 (1086, 1863)	16.1 (14, 18.5)	21.4 (16.8, 27.6)	−8.2 (−24.6, 11.3)	39,453 (29,635, 52,553)	13.1 (11.2, 15.2)	512.5 (394.3, 674.9)	−9.6 (−27.4, 12.1)
Western Sub‐Saharan Africa	11,595 (9622, 13,931)	5.9 (5.2, 6.7)	6.7 (5.7, 8)	−1.6 (−15.9, 15.5)	294,397 (239,268, 358,661)	4.8 (4.1, 5.4)	152.2 (125.6, 183.5)	−4.6 (−19.4, 13.3)
Eastern Sub‐Saharan Africa	14,479 (11,831, 17,806)	7.6 (6.7, 8.7)	9.6 (8, 11.7)	−15.7 (−26.3, −3.8)	379,002 (302,179, 476,721)	5.7 (4.9, 6.7)	222.9 (180.7, 274.9)	−17.2 (−28.5, −3.5)
Central Sub‐Saharan Africa	5054 (3608, 7121)	8.7 (7.3, 11.1)	9.9 (7.1, 13.7)	−28.6 (−45.3, −8.9)	140,195 (99,531, 198,136)	7.3 (6, 9.4)	242 (172.7, 340.9)	−28 (−45.2, −6.2)
Southern Sub‐Saharan Africa	12,463 (11,186, 13,781)	16.8 (15.5, 18.1)	22.8 (20.6, 25.2)	−32 (−41.3, −22.4)	321,174 (287,410, 358,475)	15.7 (14.2, 17)	543.5 (487.1, 605.2)	−35.2 (−43.6, −25.7)

Abbreviations: ASRs, age‐standardized rates; DALY, disability‐adjusted life year; GBD, global burden of disease.

### 3.2 Regional level

In 2019, the total number of cancer‐related deaths attributable to smoking were highest in East Asia (924,474 [758,790 to 1,114,252]), Western Europe (332,759 [307,650 to 354,004]), and High‐income North America (256,115 [237,465 to 272,442]). In contrast, the lowest numbers were found in Oceania (1413 [1086 to 1863]), Andean Latin America (4229 [3342 to 5216]), and Central Sub‐Saharan Africa (5054 [3608 to 7121]) (Table 1). The proportion of all cancer‐related deaths (PAFs) that were attributable to smoking ranged from 5.9% to 32.8%. East Asia (32.8% [30.0 to 35.5]), Central Europe (31.0% [29.6 to 32.6]), and High‐income North American (29.5% [28.1 to 31.0]) had the three highest PAFs, while the lowest were found in Western Sub‐Saharan Africa (5.9% [5.2 to 6.7]), Andean Latin America (6.5% [5.7 to 7.3]), and Eastern Sub‐Saharan Africa (7.6% [6.7 to 8.7]) (Table 1).

The age‐standardized death rates for cancers attributable to smoking (per 100,000) in 2019 were highest in Central Europe (50.4 [44.4 to 57.6]), East Asia (45.0 [37.1 to 54.0]), and High‐income North America (40.0 [37.3 to 42.5]). The lowest rates were found in Western Sub‐Saharan Africa (6.7 [5.7 to 8.0]), Andean Latin America (7.8 [6.2 to 9.6]), and Eastern Sub‐Saharan Africa (9.6 [8.0 to 11.7]) (Table 1). All GBD regions showed decreases in the age‐standardized death rates of cancer attributable to smoking from 1990 to 2019, with the largest decreases being seen in Australasia (48.2% [‐51.1 to ‐45.6]), Central Latin America (47.3% [‐54.9 to ‐38.4]), and Tropical Latin America (41.7% [‐45.0 to ‐37.9]) (Table 1). The age‐standardized death rates of cancers attributable to smoking (per 100,000) in 2019 are presented in Figure S1, by region and sex, and the changes from 1990 to 2019 are presented in Figure S2, by region and sex.

In 2019, the total number of cancer‐related DALYs attributable to smoking were highest in East Asia (21,001,451 [16,984,703 to 25,387,046]), Western Europe (6,785,694 [6,407,173 to 7,155,155]), and High‐income North America (5,347,818 [5,042,527 to 5,634,478). The lowest number of DALYs were in Oceania (39,453 [29,635 to 52,553]), Andean Latin America (90,847 [70,790 to 113,852]), and Central Sub‐Saharan Africa (140,195 [99,531 to 198,136]) (Table 1). The proportion of all cancer‐related DALYs that were attributable to smoking ranged from 4.8% to 32.4%. Central Europe (32.4% [31.1 to 33.8]), East Asia (30.0% [27.2 to 32.6]), and High‐income North America (24.6% [23.2 to 26.1]) had the three highest PAFs, while the lowest were found in Western Sub‐Saharan Africa (4.8% [4.1 to 5.4]), Andean Latin America (5.5% [4.8 to 6.3]), and Eastern Sub‐Saharan Africa (5.7% [4.9 to 6.7]) (Table 1).

In 2019, the age‐standardized DALY rates of cancers attributable to smoking per 100,000 were highest in Central Europe (1261.1 [1103.2 to 1447.4]), East Asia (972.6 [790.5 to 1171.3]), and Eastern Europe (894.4 [780.0 to 1015.5]). Conversely, the lowest rates were found in Western Sub‐Saharan Africa (152.2 [125.6 to 183.5]), Andean Latin America (162.7 [126.9 to 203.7]), and Eastern Sub‐Saharan Africa (222.9 [180.7 to 274.9]) (Table 1). All GBD regions showed decreases in the age‐standardized DALY rates of cancer attributable to smoking from 1990 to 2019, with the largest decreases being seen in Australasia (−50.4% [−53.0 to −48.0]), Central Latin America (−49.2% [−57.1 to −40.0]), and Tropical Latin America (−44.5% [−47.7 to −41.0]) (Table 1). The age‐standardized DALY rates of cancers attributable to smoking (per 100,000) for the 21 GBD regions in 2019 are presented by sex in Figure S3, and the percentage change in the age‐standardized DALY rates of cancers attributable to smoking, from 1990 to 2019, are presented by sex for the 21 GBD regions in Figure S4.

Across the globe, the number of deaths attributable to smoking increased from 1,543,971 (1,453,808 to 1,636,637) in 1990 to 2,493,026 (UI 2,275,657 to 2,716,575) in 2019. In 2019, East Asia (924,474 [758,790 to 1,114,252]), Western Europe (332,759 [307,650 to 354,004]), and High‐income North America (256,115 [237,465 to 272,442]) had the highest number of cancer‐ related deaths attributable to smoking. Similarly, in 1990 the number of deaths were highest in East Asia (396,273 [330,289 to 463,942]), Western Europe (301,576 [288,565 to 315,180]), and High‐income North America (210,146 [199,291 to 219,932]) (Figure S5 and Table S3). East Asia, Western Europe, and High‐income North America had the highest number of DALYs, due to cancers that were attributable to smoking, in both 1990 and 2019 (Figure S6 and Table S4).

**FIGURE 1:**
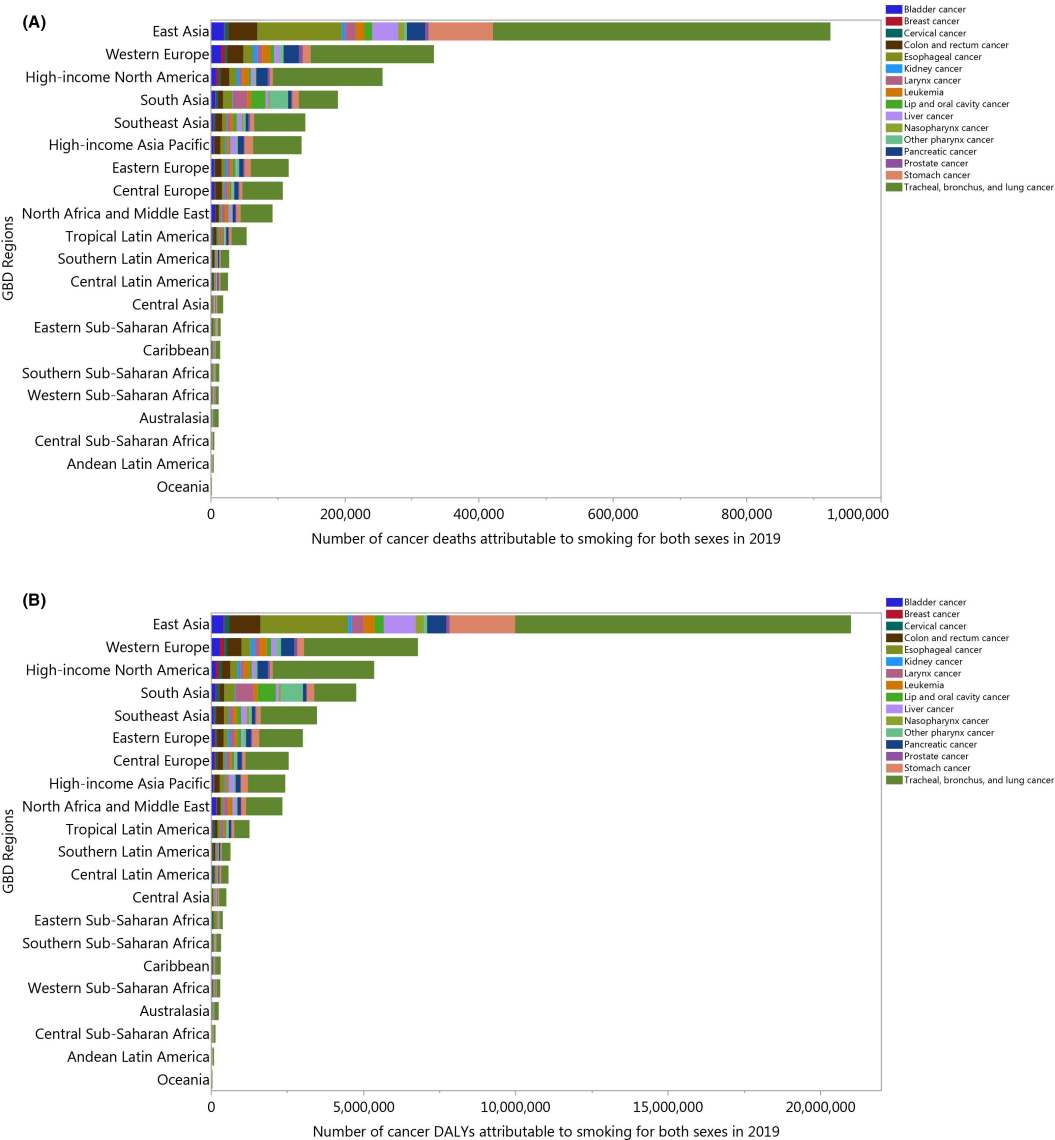
Number of cancer deaths (A) and DALYs (B) attributable to smoking in 2019 by cancer types and GBD region. DALY = disability‐adjusted life years (Generated from data available from http://ghdx.healthdata.org/gbd‐results‐tool)

The contribution of each cancer attributable to smoking also differed by region. The number of deaths due to stomach cancer were highest in East Asia (95,819 [72,688 to 120,868]), High‐income Asia Pacific (12,409 [9,832 to 14,997]), and Western Europe (11,588 [9,254 to 13,930]). In contrast, the number of deaths due to larynx cancer were highest in South Asia (21,733 [17,355 to 27,013]), East Asia (15,761 [12,530 to 19,329]), and Western Europe (6,871 [5,885 to 7,700]) (Figure 1A). The contribution of each cancer to the number of all cancer‐related DALYs also differed by region (Figure 1B).

### 3.3 National level

**FIGURE 2:**
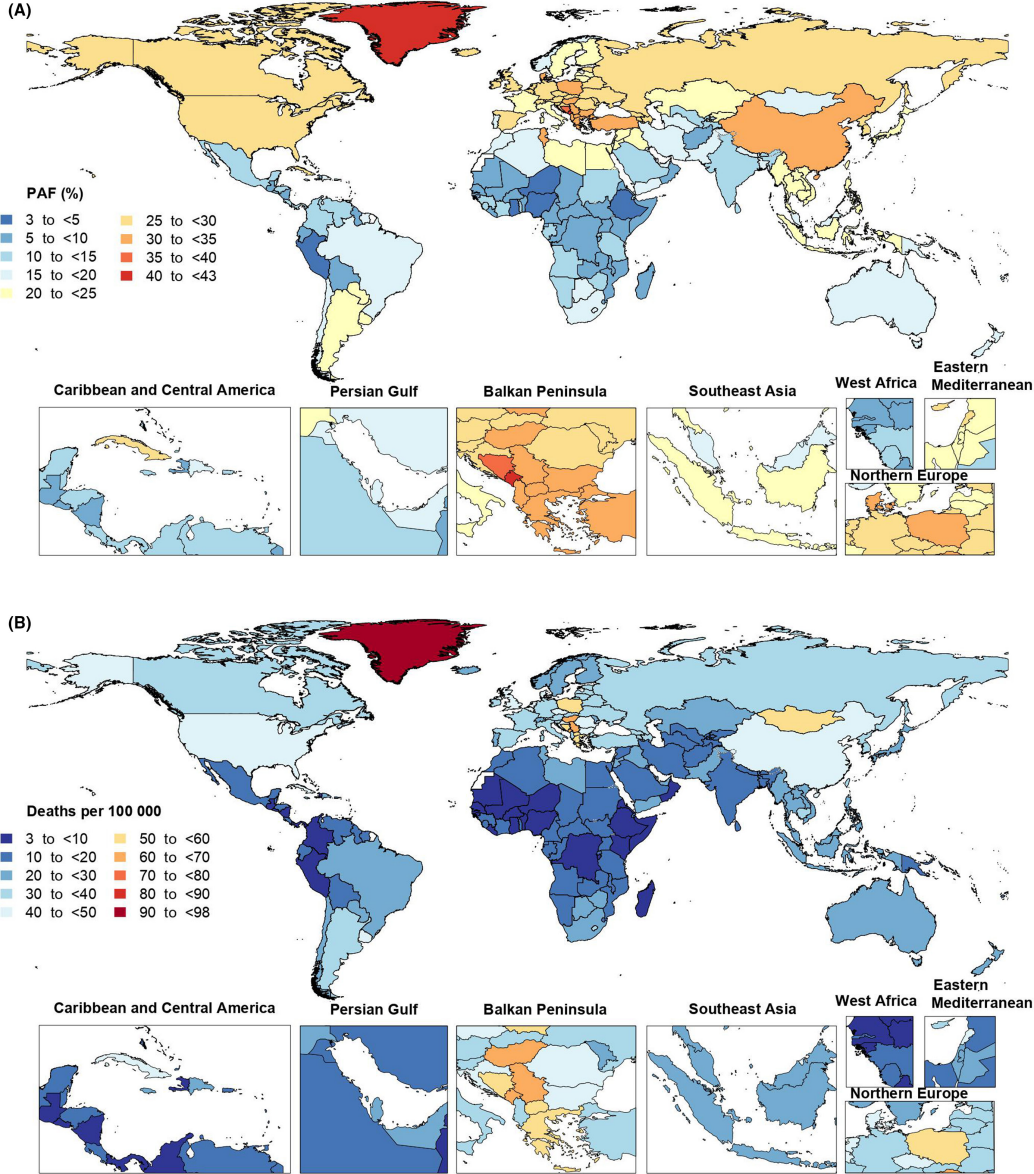
Population‐attributable fraction (PAF) (A) and age‐standardized rates (B) of cancer deaths attributable to smoking in 2019, by country. (Generated from data available from http://ghdx.healthdata.org/gbd‐results‐tool)

In 2019, the proportion of all cancer‐related deaths that were attributable to smoking differed substantially by country. Greenland (42.5% [39.9 to 45.2]), Montenegro (41.8% [39.5 to 44.1]), and Bosnia and Herzegovina (35.2% [33.5 to 37.1]) had the three highest PAFs. In contrast, the lowest PAFs were found in Ethiopia (3.2% [2.4 to 4.0]), Nigeria (3.5% [2.7 to 4.5]), and Niger (4.6% [3.7 to 5.6]) (Figure 2A and Table S3). The age‐standardized death rate of cancers attributable to smoking in 2019 ranged from 3.3 to 97.6 per 100,000. Greenland (97.6 [80.1 to 115.4]), Montenegro (68.4 [57.4 to 82.4]), and Monaco (68.3 [55.6 to 82.3]) had the three highest age‐standardized death rates for cancers attributable to smoking (per 100,000) in 2019. In contrast, the lowest rates were found in Ethiopia (3.3 [2.4 to 4.6]), Nigeria (3.8 [2.8 to 5.0]), and Niger (4.8 [3.3 to 6.5]) (Figure 2B and Table S3). Increases in the age‐standardized death rates for cancers attributable to smoking were found in several countries and territories over the measurement period. Sao Tome and Principe (54.6% [19.1 to 102.9]), Lesotho (44.0% [6.0 to 96.3]), and Egypt (36.5% [1.7 to 84.9]) had the largest increases in age‐standardized death rates over the duration of the study. In contrast, Singapore (−60.1% [−63.6 to −56.6]), Colombia (−57.6% [−67.2 to −44.9]), and Turkmenistan (−54.8% [−63.9 to −44.1]) showed the largest decreases over this period (Table S3).

The proportion of all cancer‐related DALYs that were attributable to smoking in 2019 differed considerably by country. Montenegro (41.9% [39.7 to 43.9]), Greenland (40.9% [38.1 to 43.8]), and Hungary (35.7% [34.2 to 37.3]) had the three highest PAFs. In contrast, the lowest PAFs were found in Ethiopia (2.1% [1.6 to 2.8]), Nigeria (2.8% [2.1 to 3.5]), and Niger (3.4% [2.7 to 4.1]) (Figure S7 and Table S4). The age‐standardized DALY rate of cancers attributable to smoking in 2019 ranged from 72.2 to 2224.0 per 100,000. Greenland (2224.0 [1804.5 to 2678.8]), Montenegro (1708.0 [1418.4 to 2070.1]), and Hungary (1588.6 [1290.6 to 1942.8]) had the three highest age‐standardized DALY rates. In contrast, the lowest rates were found in Ethiopia (72.2 [51.2 to 98.0]), Nigeria (80.6 [59.9 to 108.8]), and Niger (102.2 [70.0 to 142.4]) (Figure S8 and Table S4). Sao Tome and Principe (53.4% [14.9 to 106.1]), Lesotho (47.5% [4.4 to 106.8]), and Cabo Verde (31.1% [8.5 to 60.0]) were the only countries to show increases in the age‐standardized DALY rates of cancers attributable to smoking from 1990 to 2019. In contrast, Singapore (−63.7% [−67.0 to −60.6]) Colombia (−59.6% [−69.2 to −47.0]), and Bahrain (−57.7% [−68.3 to −43.7]) showed the largest decrease over the measurement period (Table S4).

### 3.4 Age and sex patterns

**FIGURE 3:**
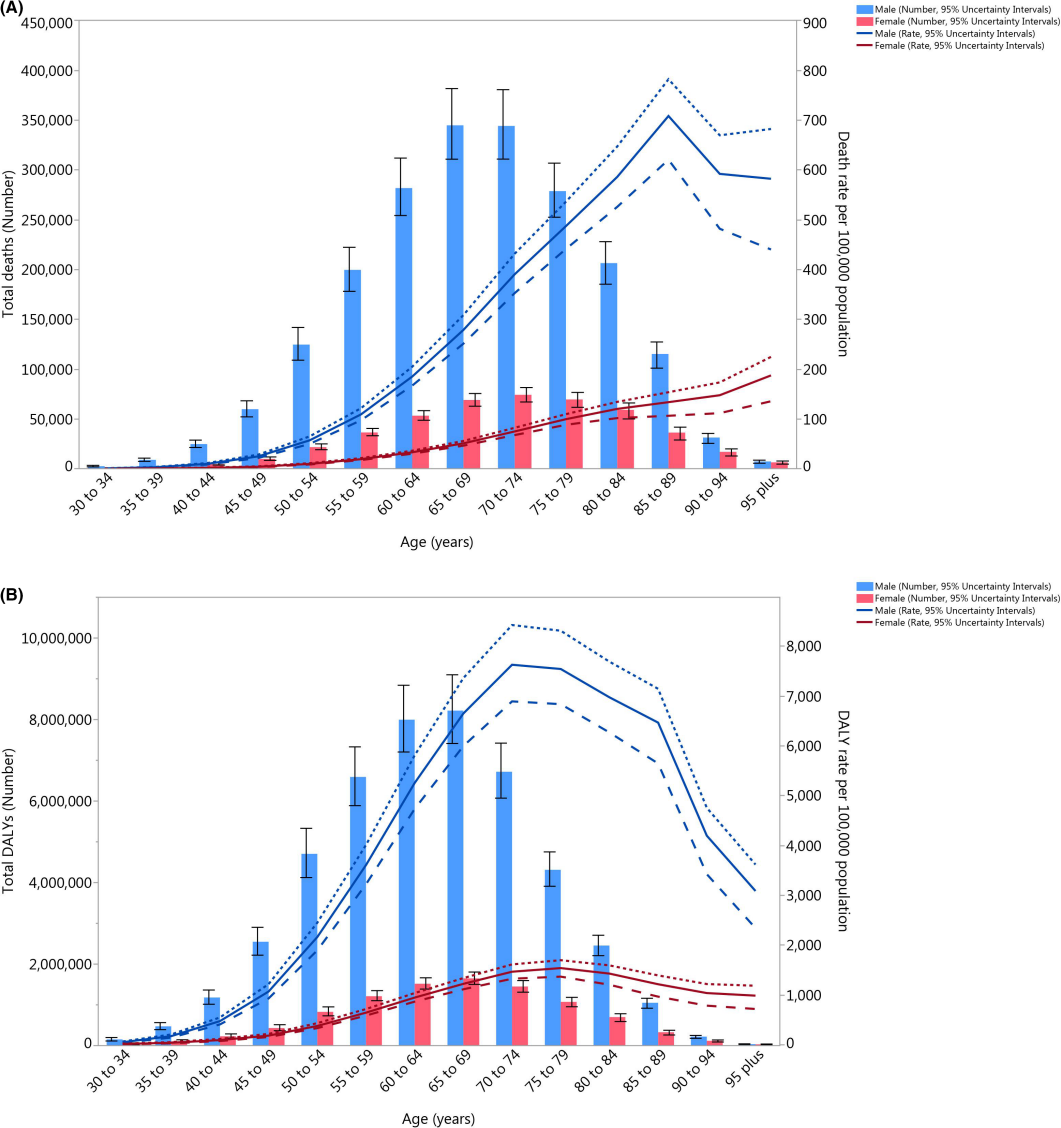
Global number of deaths and death rate (A) and global number of DALYs and DALY rate (B) of cancers attributable to smoking per 100,000 population by age and sex in 2019; Dotted and dashed lines indicate 95% upper and lower uncertainty intervals, respectively. DALY, disability‐adjusted life years. (Generated from data available from http://ghdx.healthdata.org/gbd‐results‐tool)

In 2019, the global number of deaths attributable to smoking was highest in the 65–69 and 70–74 age group for males and females, respectively. The death rate attributable to smoking started to increase from the 40–44 age group and peaked in the 85–89 and 95^+^ age group for males and females, respectively. There were substantial differences between males and females, in terms of the number of deaths and the death rate (Figure 3A). Furthermore, in 2019 the global number of DALYs was highest in the 65–69 age group. For males, the DALY rate started increasing in the early age groups, peaking in the 70–74 age group and then decreasing. For females, the DALY rate started to increase during the middle ages, with the highest DALY rate being in the 75–79 age group (Figure 3B).

### 3.5 Burden of cancers attributable to smoking by socio‐demographic index (SDI)

**FIGURE 4:**
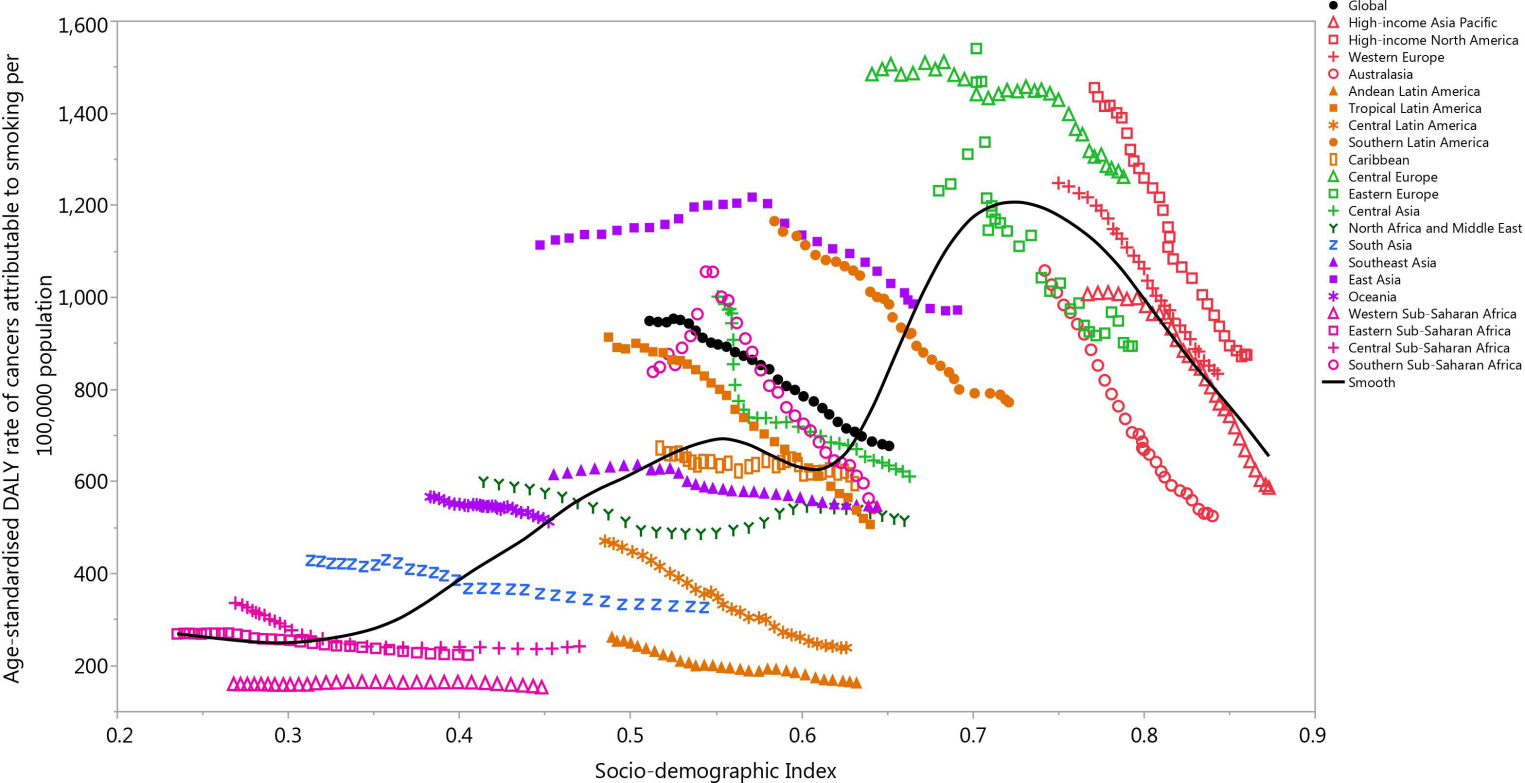
Age‐standardized DALY rates of cancers attributable to smoking for 21 Global Burden of Disease regions by socio‐demographic index, 1990–2019; Expected values based on socio‐demographic index and disease rates in all locations are shown as the black line. Thirty points are plotted for each GBD region and show the observed age‐standardized DALY rates from 1990 to 2019 for that region. DALY, disability‐adjusted life years. (Generated from data available from http://ghdx.healthdata.org/gbd‐results‐tool)

There was generally a positive association between the regional SDI and the corresponding age‐standardized DALY rates of all cancers attributable to smoking, from 1990 to 2019. Regions higher than the solid black line had a higher than expected burden (based on SDI), while those below the line had a lower than expected burden. Most of the GBD regions showed a decrease in the age‐standardized DALY rates across the measurement period. High‐income North America, Western Europe, and Central Europe had higher than expected burdens for all years during this period. In contrast, the burden of cancers attributable to smoking was lower than expected for Australasia, Central Latin America, Andean Latin America, and Western Sub‐Saharan Africa. Eastern Europe, East Asia, Southeast Asia, Southern Latin America, Tropical Latin America, the Caribbean, Central Sub‐Saharan Africa, and Eastern Sub‐Saharan Africa had a higher than expected burden in the early years of the measurement period, but their burden became lower than expected during the later years of the measurement period (Figure 4). A similar pattern was also found in the relationship between the regional SDI and the corresponding age‐standardized death rates of all cancers attributable to smoking, from 1990 to 2019 (Figure S9). The association between the age‐standardized DALY rate of cancers attributable to smoking in 2019 and each country's SDI was also generally positive (Figure S10). Countries and territories such as Greenland, Montenegro, Hungary, Monaco, and Serbia had much higher than expected levels of burden. In contrast, the corresponding burdens were much lowest than expected for several countries, including Singapore, Puerto Rico, Oman, Peru, and Nigeria (Figure S10). A similar pattern was also observed in the relationship between the age‐standardized death rate of cancers attributable to smoking in 2019 and each country's SDI (Figure S11).

## 4 DISCUSSION

The findings of the current study show that despite a substantial decrease in the age‐standardized burden of smoking‐attributable cancers over the period 1990–2019, there were 2.5 million deaths and 56.4 million DALYs from cancers attributable to smoking in 2019, which is substantial. The burden was found to be considerably higher among men and the elderly. There was also an almost positive association found between SDI and the age‐standardized DALY rate.

The GBD 2015 smoking study reported that cancer was the second largest cause of smoking‐attributable age‐standardized DALYs, with an attributable proportion of 27.6%, which was only lower than cardiovascular diseases (41.2%).^33^ In accordance with their findings, our results showed that 24.7% of deaths and 22.5% of all cancers‐related DALYs were attributable to smoking in 2019. Similar to our results, previous research found that lung cancer was the most common cancer attributable to smoking in men and women in 2015.^33^ The GBD 2019 smoking study performed dose–response meta‐regression analyses for 36 health outcomes, including some types of cancers caused by smoking, but the attributable burden of cancers were not reported.^7^ To the best of our knowledge, no previous study has reported the global burden of all cancers attributable to smoking, which is a unique contribution of our study.

Regionally, we found that Central Europe and East Asia had the largest age‐standardized death and DALY rates of cancers attributable to smoking in 2019. Interestingly, a study conducted in 2019 found that the prevalence of smoking was higher among men in East and Southeast Asia and was higher among women in Central Europe and Southern Latin America.^7^ Furthermore, according to Yang et al., Asian men start smoking at an earlier age and smoke a higher number of cigarettes per day than do Asian women.^34^ Similarly, Martiniuk et al. showed that the prevalence of smoking in the WHO Asia Pacific region was 18%–65% and 0%–50% in males and females, respectively.^35^ Therefore, the greater attributable burden of cancer in these regions might be due to a higher prevalence of this substantial risk factor (i.e., smoking). A study evaluating death and cancer incidence data in the Association of Southeast Asian Nations member countries showed that in 2012 tobacco smoking was responsible for 30.5% of the cancer‐related mortality in these countries and that lung cancer made up the single largest proportion.^36^ The Western and Eastern Sub‐Saharan Africa regions were among the regions with the lowest smoking‐attributable burden of cancers in our study. The potential reasons for the lower attributable burden in these regions might be the absence of cancer registries in some Sub‐Saharan African countries^37^ or a lower prevalence of smoking in Sub‐Saharan Africa, compared with other regions.^7^ A study found that joining up to the WHO tobacco control treaty was associated with a decrease in cancer burden in most Sub‐Saharan African countries, like Nigeria, Ethiopia, Ghana, Kenya, and South Africa.^38^

In a nationwide study in Canada, it was revealed that 20.1% and 14.7% of all cancers were attributable to active tobacco smoking in males and females, respectively.^17^ Our results showed that Canada, as a country in High‐income North America, had 29.5% of attributable DALYs in men and 23.5% in women were due to smoking. The discrepancy could be as a result of differences in the methodology used by these two studies, such as the use of different inclusion criteria and study populations. Moreover, our research found that in 2019, China, a country in East Asia, PAFs for deaths attributable to smoking were 46.3% in males and 9.4% in females. However, in 2013 a systemic assessment of cancer mortality attributable to smoking in China showed that men had a higher proportion than women (29.3% vs. 2.6%).^39^ These discrepancies could be due to different methodological techniques, including different data registries, definitions of tobacco smoking, or time ranges that the studies used.

The present research found that the global age‐standardized cancer‐related deaths attributable to smoking was much greater in men than among women (54.6 vs. 10.5 per 100,000 population). This finding supports the research of Yang and colleagues, who evaluated the proportion of lung cancer mortality attributable to smoking and reported an age‐standardized death rate of 26.69 (95% UI: 25.72 to 27.61) in males and 5.1 (95% UI: 4.81 to 5.42) in females.^40^ This pattern of findings is also in accordance with most research from different regions and countries.^17^, ^35^, ^40^, ^41^, ^42^, ^43^ Gender discrepancies in tobacco smoking and the smoking‐attributable burden of cancers may have different personal, social, or cultural reasons, one of which is the prevalence of smoking.^44^ The area with a high prevalence of smoking should be targeted with smoking cessation program through channels such as mass media campaigns, which also encourage utilization of evidence‐based cessation support initiatives, such as Quitlines.^45^

This research also found that the age‐standardized rate of DALYs and deaths from cancers attributable to smoking increased with age in both sexes and was highest in adults over 70 years of age. Similar to our findings, research in 2017 reported that the global burden of lung cancer attributable to smoking increased sharply after the age of 55.^40^ This may be due to the fact that older individuals have had a longer duration of exposure to risk factors (e.g., smoking), therefore increasing the attributable mortality and morbidity.^40^

Globally, smoking‐attributable lung cancer deaths had a close to positive association with SDI quintiles in 2017, in which high‐middle and low SDI quintiles had the highest and lowest age‐standardized mortality rates (19.58 vs. 5.13 per 100,000 population).^40^ Furthermore, research in 2012 on the relationship between the human development index (HDI) and the PAFs from gastric cancer attributable to smoking found that there was a negative association in men and a positive association in women, in which very high HDI countries had the lowest and highest values in males and females, respectively (17.2% and 4.3%).^41^ Moreover, our results showed a positive association between SDI and the burden of cancer attributable to smoking. This could be due to the fact that smoking was more prevalent among those in the higher SDI quintiles, so that the burden of attributable cancers in these countries would also be higher.^41^ Despite a small number of articles reporting the association between SDI levels and some limited types of cancers, as mentioned above our study is the first to report the burden of cancers attributable to smoking by SDI.

The WHO Framework Convention on Tobacco Control (WHO FCTC) has suggested different measures to control tobacco smoking.^46^ Many of these measures can be categorized into two groups, which are: (1) supply reduction (e.g., banning the sale of tobacco products via vending machines, the Internet or any other technology‐based methods and the prohibition of cigarette sales in small packets or individually) and (2) demand reduction measures. The demand reduction measures could be implemented via the cessation of tobacco advertising and sponsorship (e.g., by the prohibition of smoking in public places) and applying taxes and increasing the price of tobacco products.^38^, ^46^ A review article by Bafunno et al. on smoking cessation programs in Europe, showed that interventions targeting the social environment, in addition to individual factors, may be more effective.^45^ Moreover, school‐based educational interventions, providing smoke‐free environments, mass media interventions, health warnings and packaging, and increasing taxes on tobacco and cigarettes have been implemented in European countries.^45^ In addition to these recommended measures, using the Internet is superior to print material and is equivalent to telephone and in‐person counseling.^47^ Due to the coronavirus disease 2019 (COVID‐19) pandemic, policy makers should focus more on online methods for smoking cessation interventions in this period or even continue it after easing the lockdown.^48^

Increasing the uptake of existing evidence‐based smoking cessation support should be a focus of investment for global tobacco control. Pharmacotherapy and counseling remain the gold standard for smoking cessation,^49^ which are unfortunately underutilized in practice.^50^ Efforts to increase uptake of these existing strategies should supplement public policy efforts, as outlined by the WHO FCTC. Opportunities are available to increase offers of support by training health professionals in smoking cessation, as outlined in our own original research studies. Our Cochrane reviews have demonstrated that delivering even brief 1‐hour training to health professionals in a primary care setting^51^ and community pharmacists^52^ can significantly increase the success of long‐term attempts to quit and result in sustained abstinence. Our own original research studies have also shown that existing evidence‐based treatments for smoking cessation are still highly effective in helping smokers quit.^53^, ^54^, ^55^ For example, in our multi‐center randomized controlled trial (n = 392), among smokers who only received Quitline support, sustained smoking abstinence of 21.4% was observed after 12‐months^56^ and 18.8% after 2 years.^53^ These studies clearly indicate that existing strategies for smoking cessation have not been exhausted and using these approaches will provide implementation ready approaches for nationwide, low‐cost cessation support.

### 4.1 Strengths and limitations of this study

To our knowledge, this is the most up‐to‐date, comprehensive, and leading study that used GBD estimates to report the burden of different types of cancers attributable to smoking at the global, regional, and national levels and according to SDI. However, we acknowledge that this study has several limitations and they should be considered when interpreting the results. First, smokeless tobacco products, heated tobacco products, chewed tobacco, smoked plants like cannabis, and other electronic nicotine delivery systems (e.g., e‐cigarettes) were not included in this study, with only smoked tobacco products considered. Moreover, the different types or subtypes of cancers attributable to smoking were not reported. Second, the data on smoking were based on self‐reports and cancer registries, which may introduce inaccuracies and biases. However, there are also limitations to cancer registries, especially in less developed countries which may lead to under reporting. Third, since the risk‐outcome was minimal for the population aged 0–29 years, only adults aged ≥30 years were included in this study. Fourth, our estimates of the smoking‐attributable burden of cancers was restricted by the availability of data, particularly in regions with a high burden of smoking or cancers. Despite these limitations our findings are highly consistent with previous studies with different methods and limitations.^33^, ^40^

## 5 CONCLUSIONS

This study shows that almost one in every four deaths, and one in every five DALYs, due to cancer was as a result of exposure to smoking. Despite declines in the burden of cancers attributable to smoking since 1990, smoking is still a substantial risk factor, especially among elderly males and in higher SDI quintiles. The findings of this study highlight the need for a renewed global effort in smoking cessation and tobacco control, given that one of the most common reasons for cessation is disease prevention. Also, effective global, regional, and national tobacco control regulations need to be adopted, properly implemented and evaluated to achieve decreases in smoking and therefore a reduction in the burden of its attributable cancers. Policymakers and physicians should provide flexible, integrated, and community‐based methods for smoking cessation programs.

## CONFLICT OF INTEREST

None declared.

## AUTHOR CONTRIBUTIONS

SS, MA, and AAK designed the study. SS, MAM, and AAH analyzed the data and performed the statistical analyses. SS, SAN, KCC, JK, NLB, MML, MJMS, GC, and AAK drafted the initial manuscript. All authors reviewed the drafted the manuscript for critical content. All authors approved the final version of the manuscript.

## DECLARATION

None of the authors listed on the manuscript are employed by a government agency that has a primary function other than research and/or education. Also, none of the authors are submitting this manuscript as an official representative or on behalf of the government.

## AUTHOR NOTE

This study is based on publicly available data and solely reflects the opinion of its authors and not that of the Institute for Health Metrics and Evaluation.

## ETHICS APPROVAL

The present study was approved by the ethics committee of the Shahid Beheshti University of Medical Sciences, Tehran, Iran. (IR.SBMU.RETECH.REC.1399.1135).

## PATIENT CONSENT FOR PUBLICATION

Not required.

## PATIENT AND PUBLIC INVOLVEMENT

Patients and the public were not involved it the analyses or preparation of this manuscript.

## Supporting information


Figure S1

Figure S2

Figure S3

Figure S4

Figure S5

Figure S6

Figure S7

Figure S8

Figure S9

Figure S10

Figure S11

Table S1

Table S2

Table S3

Table S4

## Data Availability

The data used for these analyses are all publicly available at http://ghdx.healthdata.org/gbd‐results‐tool.
