# Supplementary figures and images for: Global, regional, and national burden of cancers attributable to tobacco smoking in 204 countries and territories, 1990–2019
Source: Cancer Med. 2022 May 27;11(13):2662–78. doi: 10.1002/cam4.4647 (PMC9249976; doi:10.1002/cam4.4647)

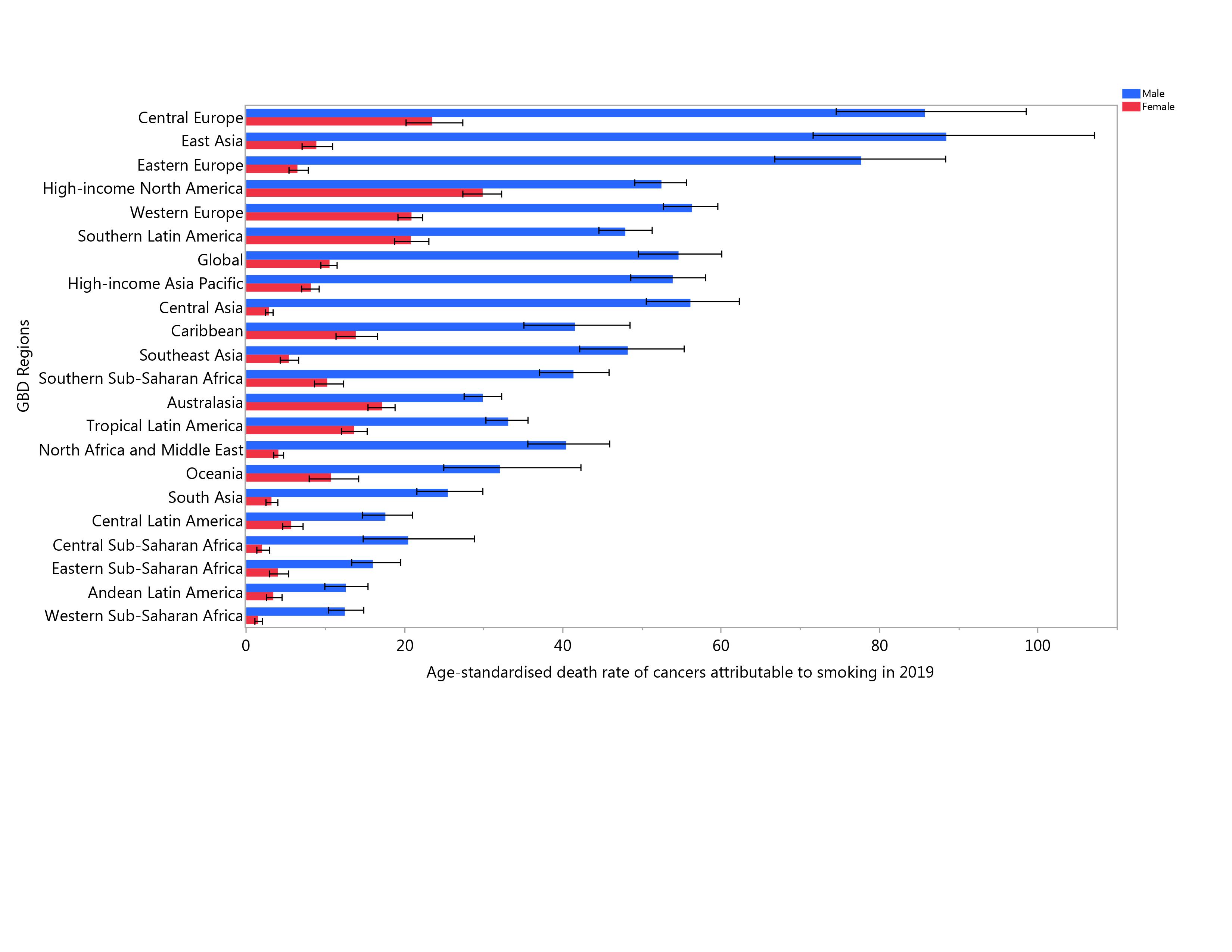

Supplement: Supplementary file 1 — Figure S1 [file CAM4-11-2662-s003.jpg]

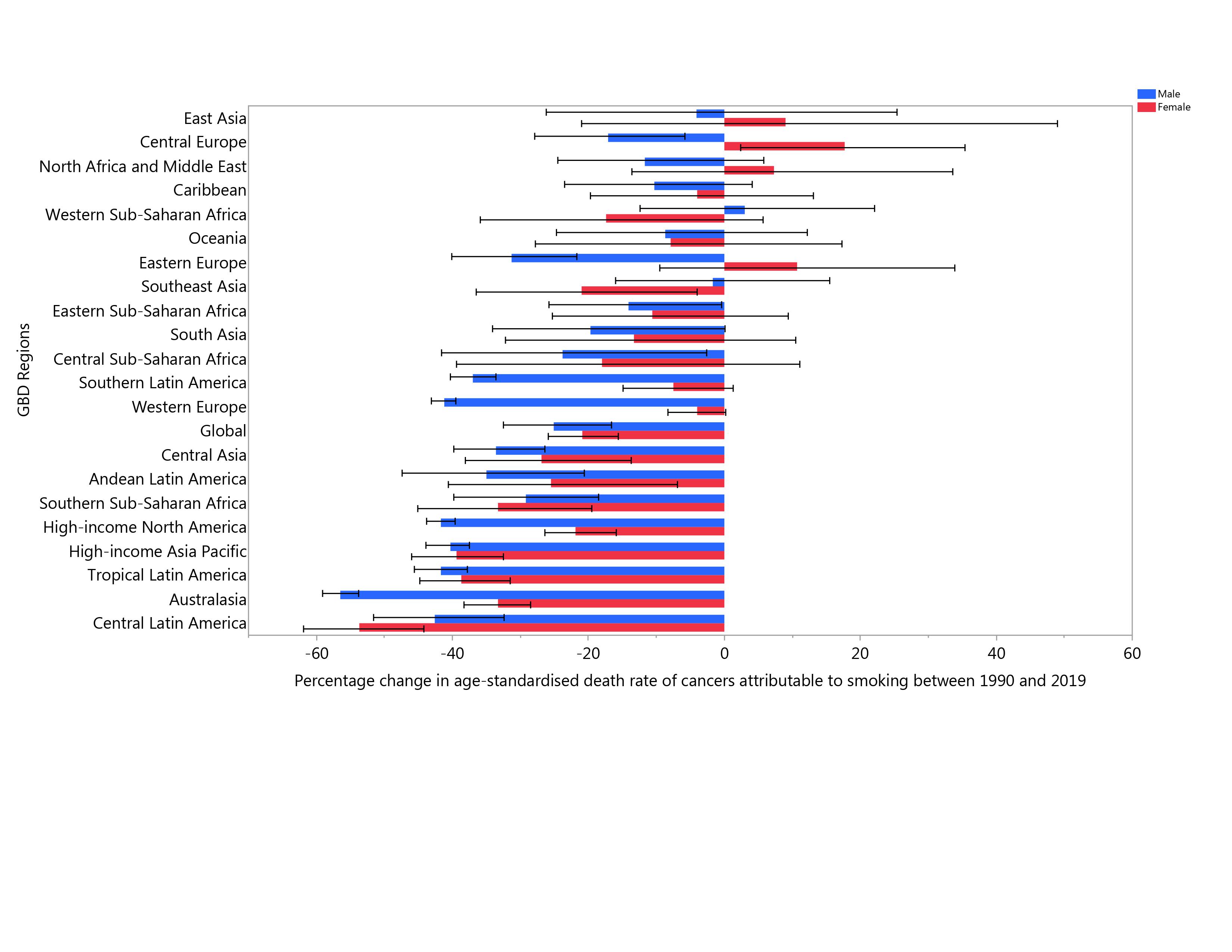

Supplement: Supplementary file 2 — Figure S2 [file CAM4-11-2662-s002.jpg]

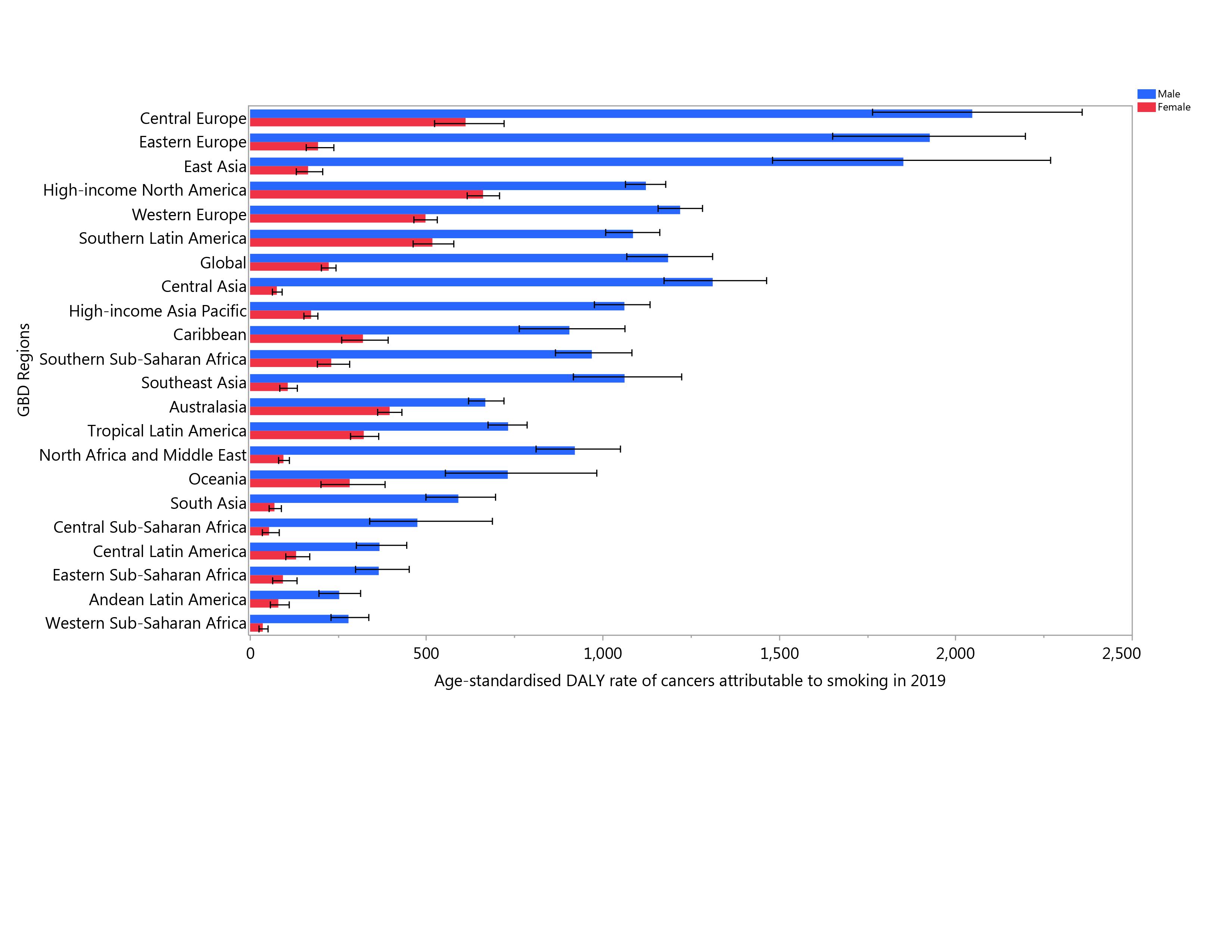

Supplement: Supplementary file 3 — Figure S3 [file CAM4-11-2662-s008.jpg]

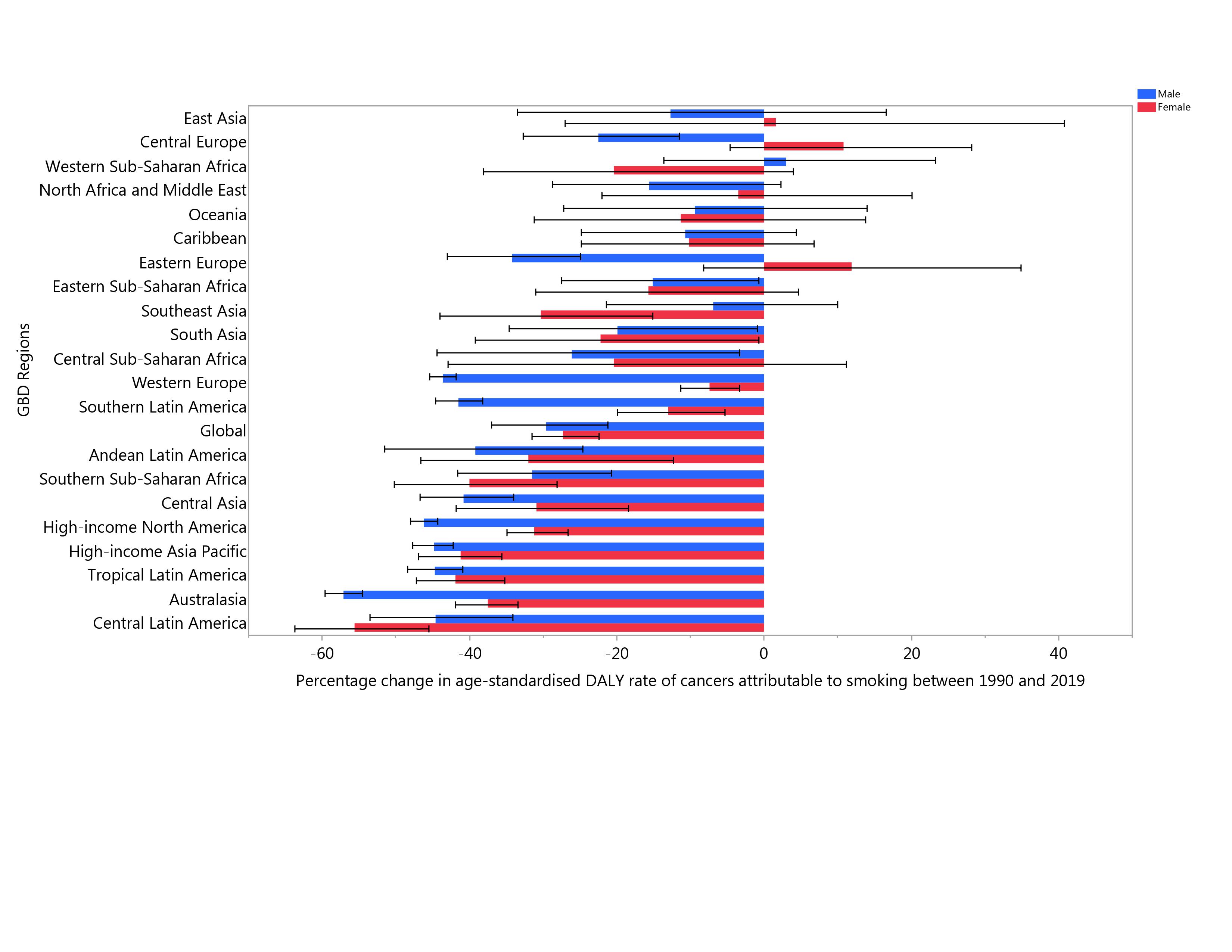

Supplement: Supplementary file 4 — Figure S4 [file CAM4-11-2662-s005.jpg]

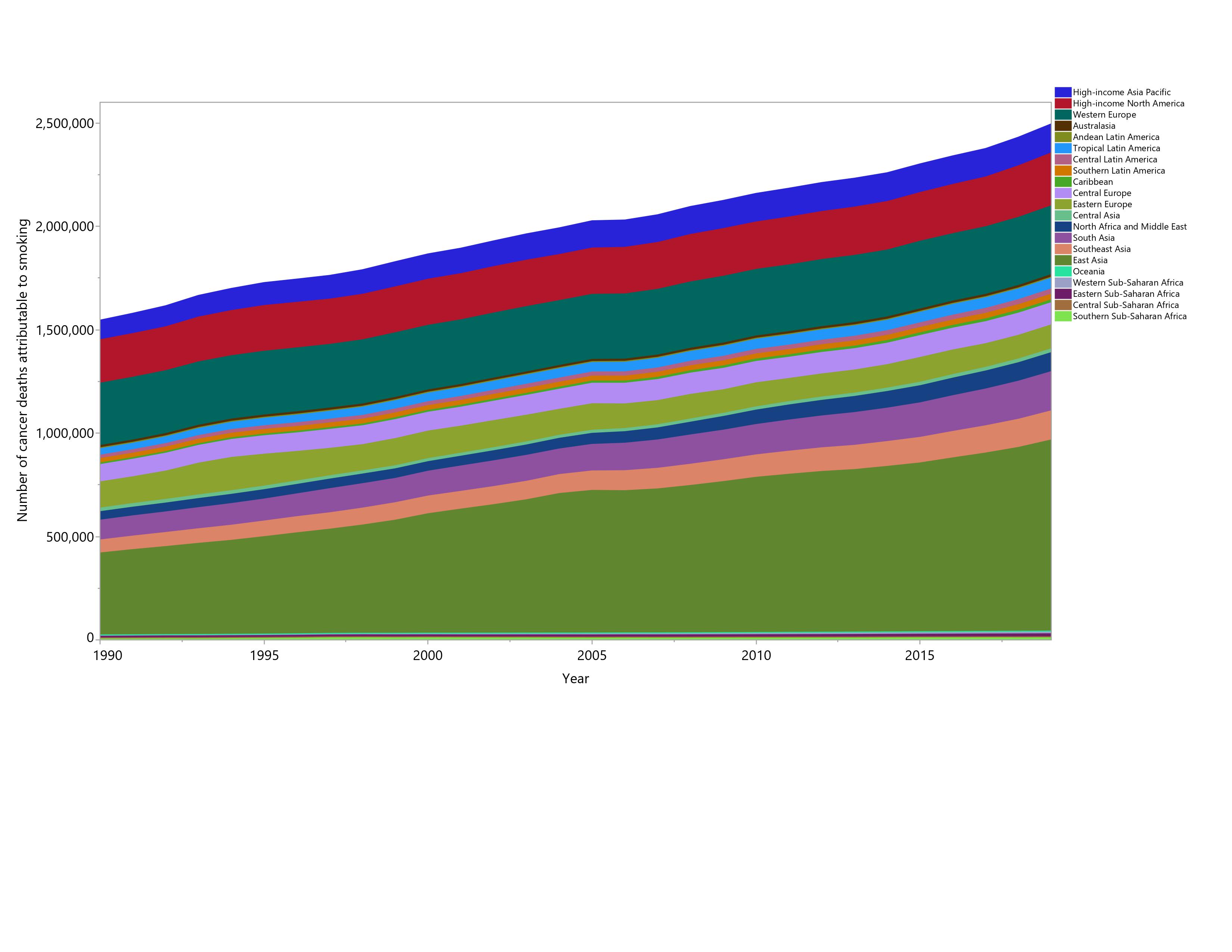

Supplement: Supplementary file 5 — Figure S5 [file CAM4-11-2662-s012.jpg]

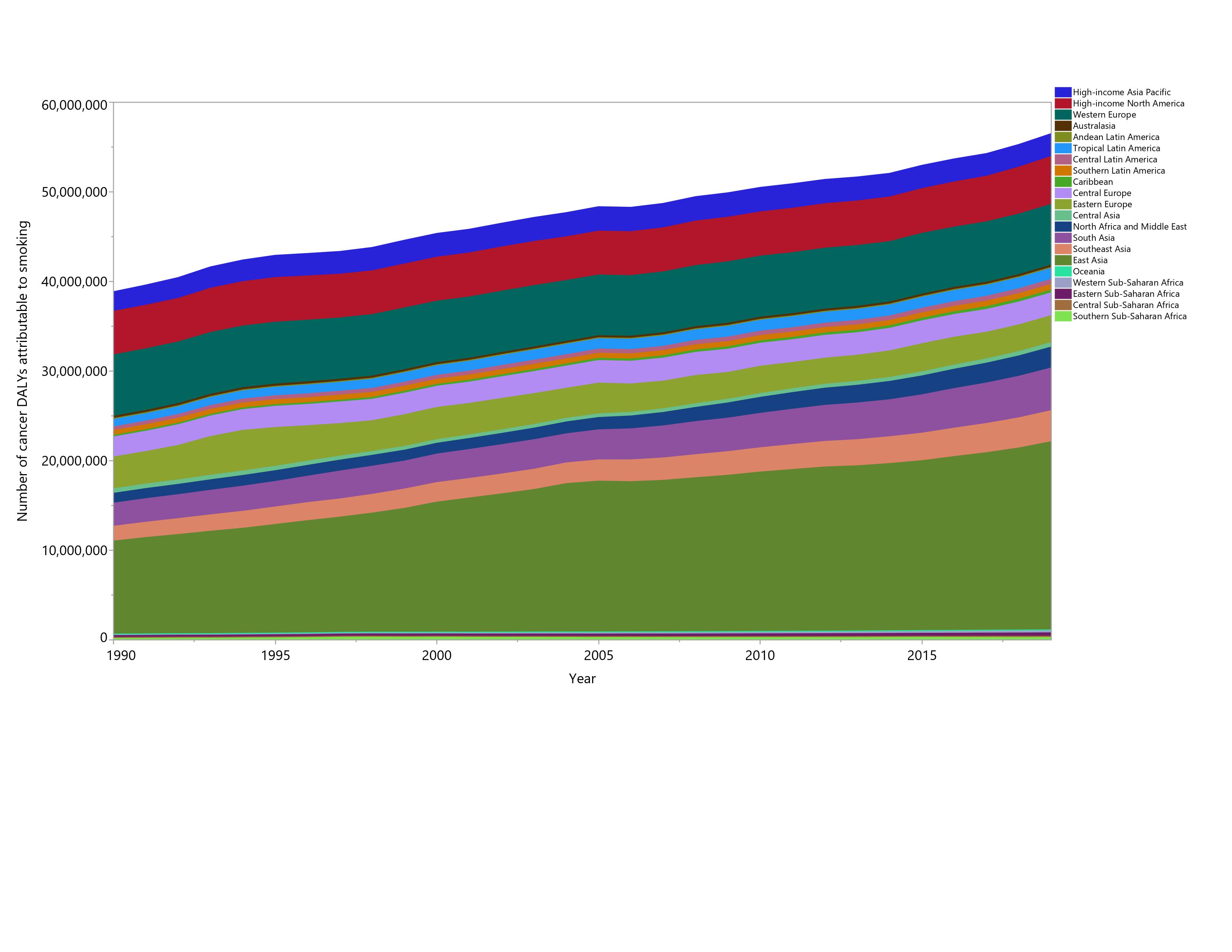

Supplement: Supplementary file 6 — Figure S6 [file CAM4-11-2662-s014.jpg]

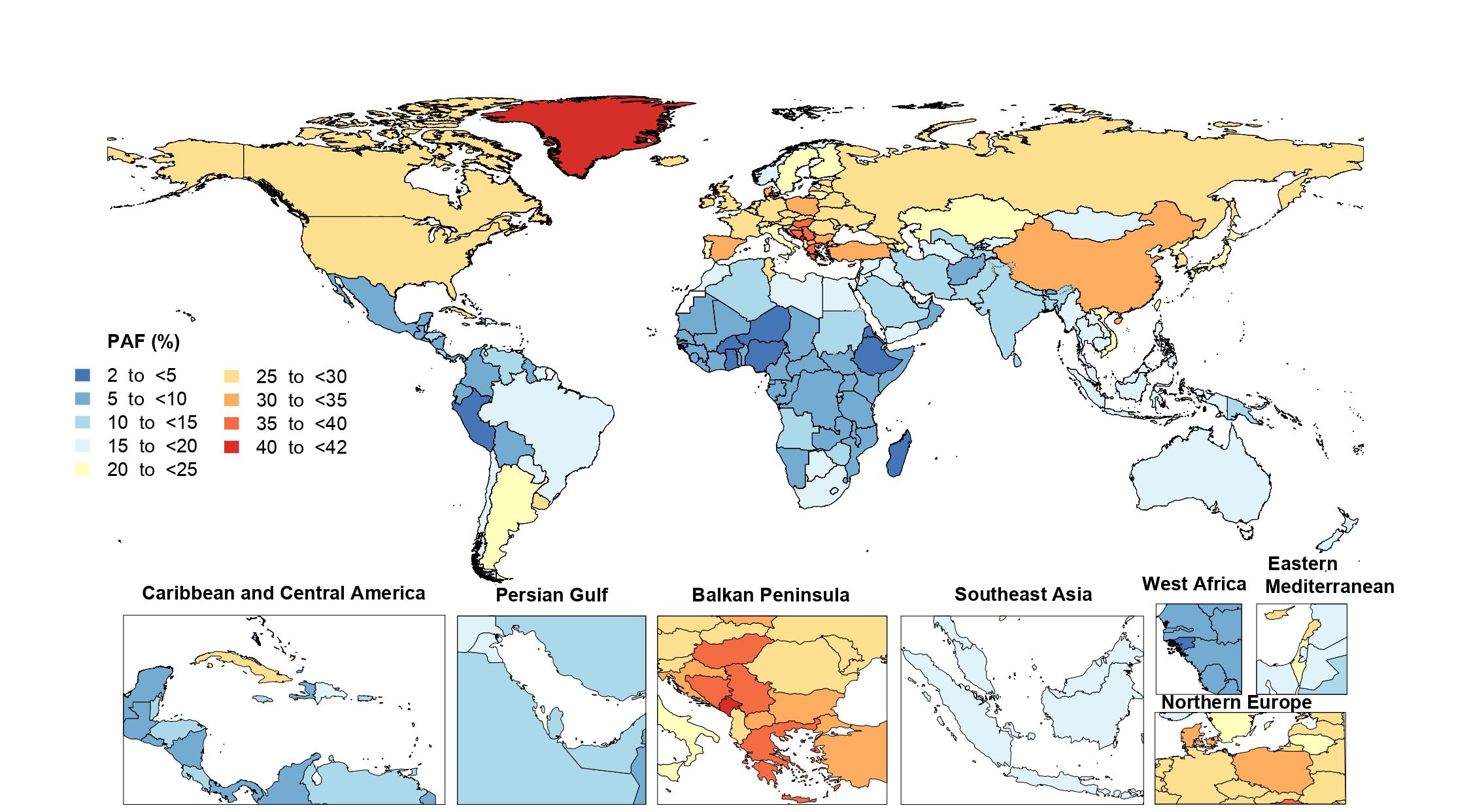

Supplement: Supplementary file 7 — Figure S7 [file CAM4-11-2662-s004.jpg]

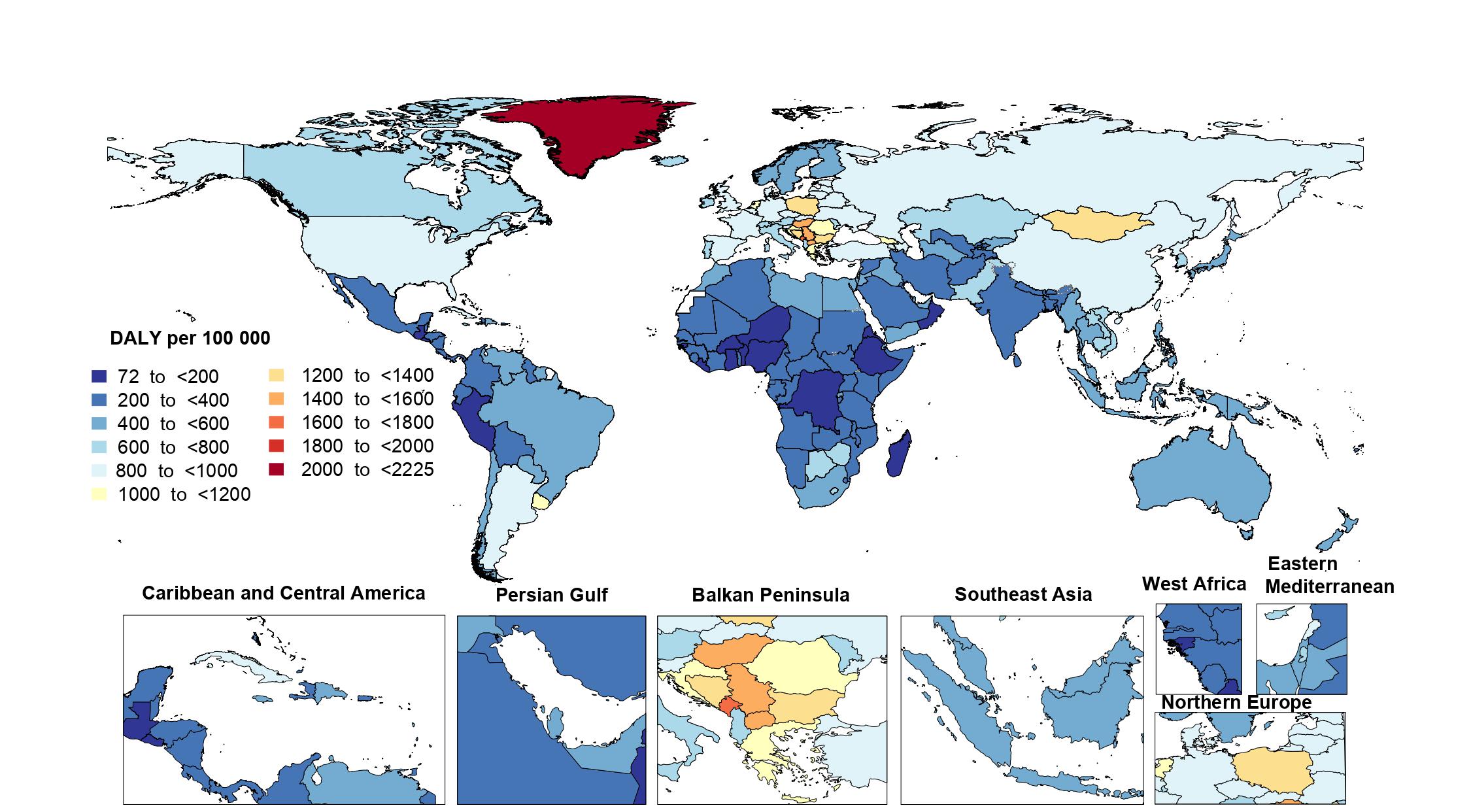

Supplement: Supplementary file 8 — Figure S8 [file CAM4-11-2662-s007.jpg]

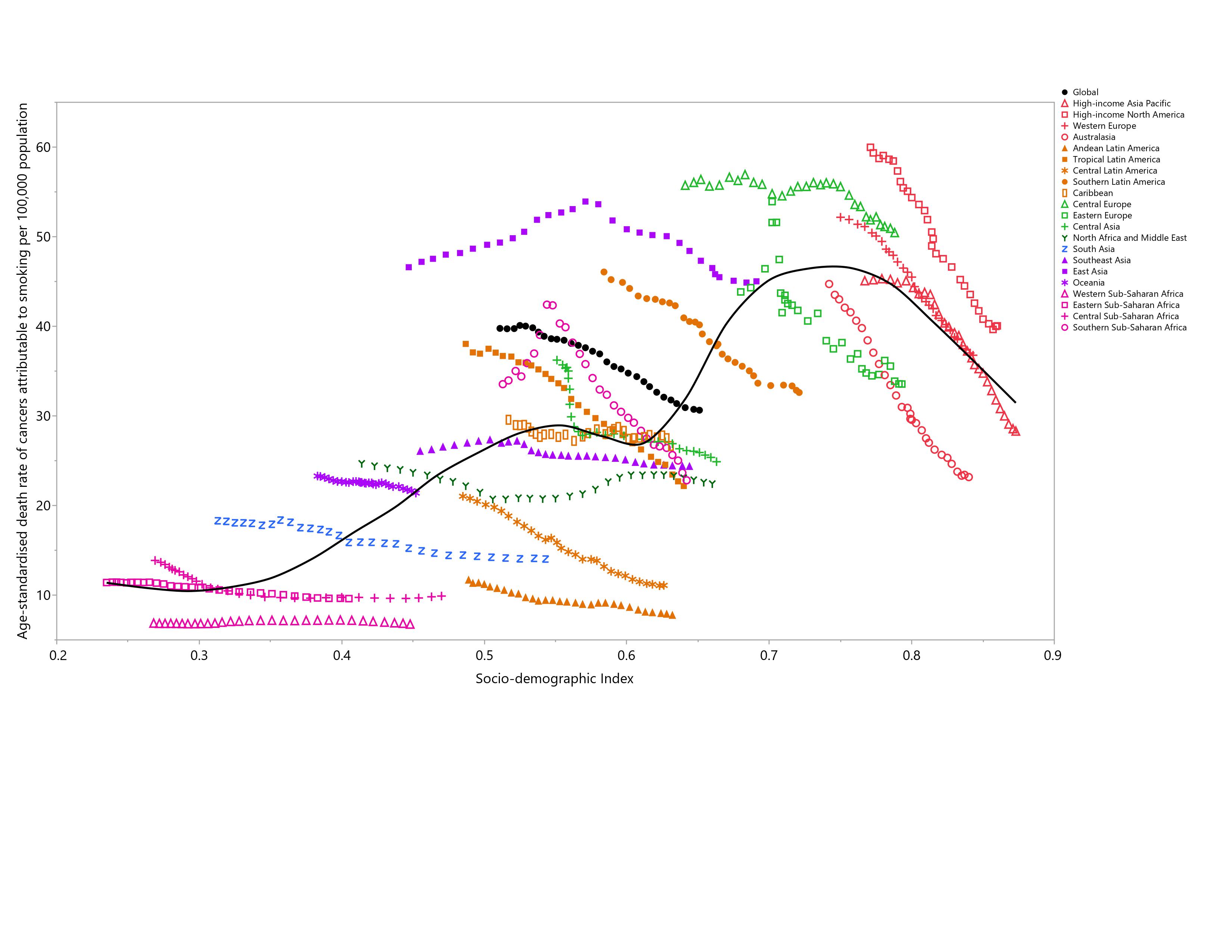

Supplement: Supplementary file 9 — Figure S9 [file CAM4-11-2662-s015.jpg]

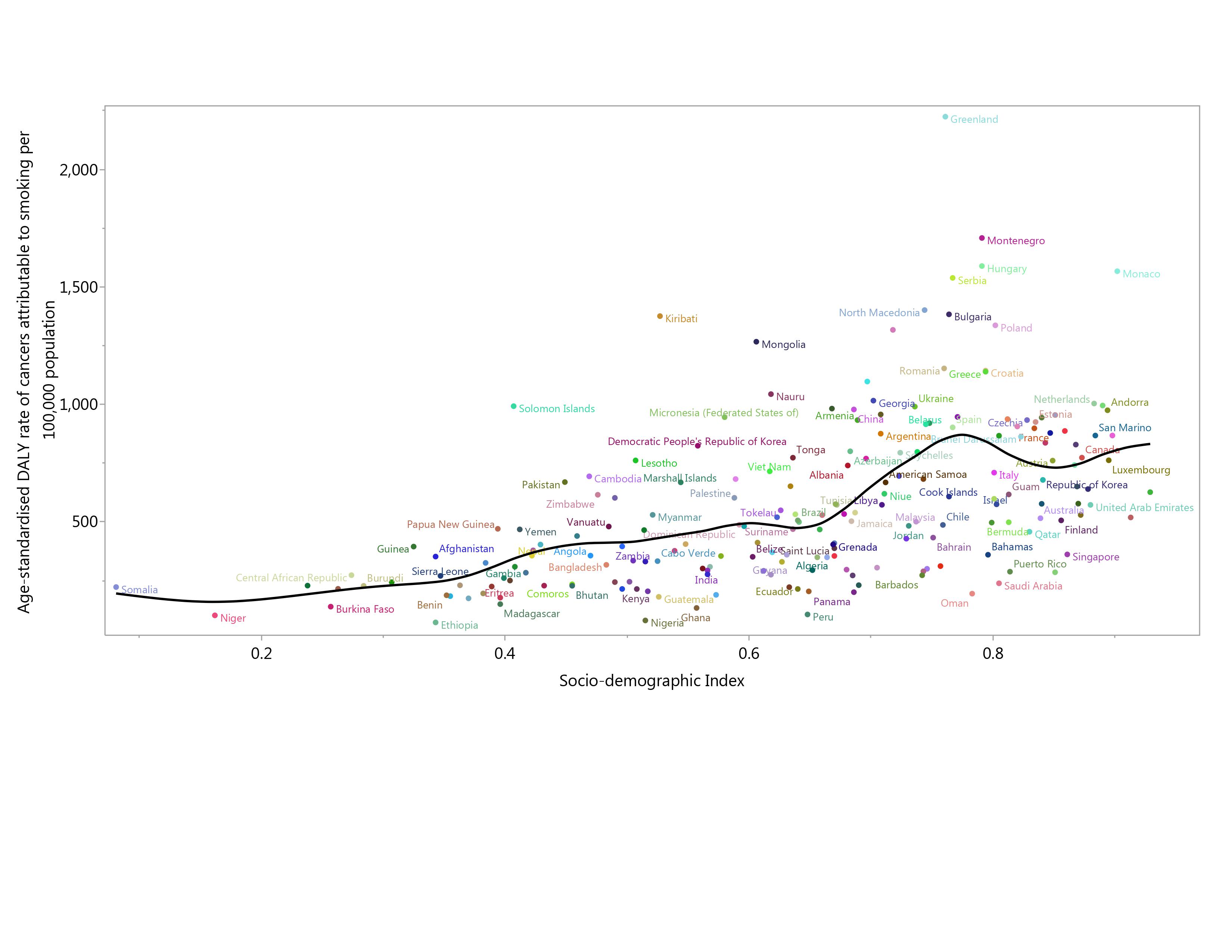

Supplement: Supplementary file 10 — Figure S10 [file CAM4-11-2662-s001.jpg]

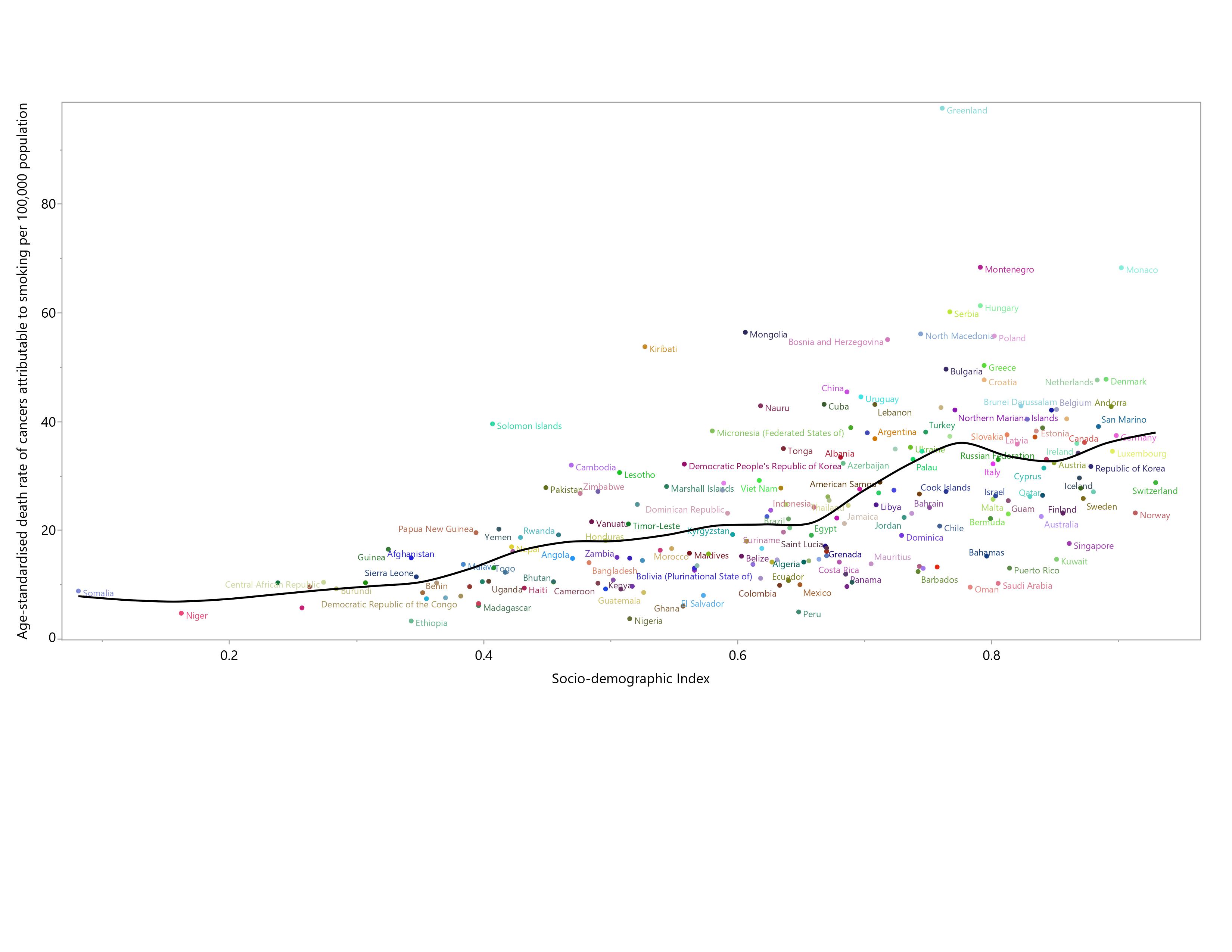

Supplement: Supplementary file 11 — Figure S11 [file CAM4-11-2662-s010.jpg]
