# Supplementary material for: Global, regional, and national burden of cancers attributable to tobacco smoking in 204 countries and territories, 1990–2019
Source: Cancer Med. 2022 May 27;11(13):2662–78. doi: 10.1002/cam4.4647 (PMC9249976; doi:10.1002/cam4.4647)
Supplement: Supplementary file 12 — Table S1 [file CAM4-11-2662-s006.doc]

| **Table S1: Number, proportion and age-standardised rates of cancer deaths attributable to smoking (per 100,000) in 2019, by sex and location (Generated from data available from http://ghdx.healthdata.org/gbd-results-tool)** | | | | | | |
| --- | --- | --- | --- | --- | --- | --- |
|  | **Male** | | | **Female** | | |
|  | **No**  **(95% UI)** | **PAF**  **(95% UI)** | **ASRs per 100,000 (95% UI)** | **No**  **(95% UI)** | **PAF**  **(95% UI)** | **ASRs per 100,000 (95% UI)** |
| **Global** | **2031296 (1835038 , 2239441)** | **35.5 (34 , 37.2)** | **54.6 (49.5 , 60.1)** | **461730 (414095 , 503790)** | **10.6 (9.9 , 11.3)** | **10.5 (9.4 , 11.5)** |
| **High-income North America** | **152088 (142144 , 161167)** | **32.8 (30.9 , 34.6)** | **52.4 (49.1 , 55.6)** | **104027 (94666 , 112679)** | **25.8 (24.2 , 27.2)** | **29.9 (27.4 , 32.3)** |
| **Canada** | **15123 (13861 , 16390)** | **30.3 (28.2 , 32.5)** | **46.5 (42.6 , 50.3)** | **10427 (9248 , 11502)** | **24.1 (22.3 , 25.9)** | **27.6 (24.9 , 30.2)** |
| **Greenland** | **42 (34 , 50)** | **44.9 (41.9 , 48.2)** | **115.2 (93.8 , 135.1)** | **25 (20 , 30)** | **39 (35.4 , 42.5)** | **78.6 (63.8 , 95.5)** |
| **United States of America** | **136920 (128037 , 145112)** | **33.1 (31.2 , 35)** | **53.2 (49.7 , 56.4)** | **93574 (85158 , 101398)** | **26 (24.4 , 27.4)** | **30.1 (27.6 , 32.6)** |
| **Australasia** | **6973 (6399 , 7538)** | **19.3 (17.9 , 20.7)** | **29.9 (27.5 , 32.3)** | **4550 (4032 , 5015)** | **16.2 (15 , 17.4)** | **17.2 (15.4 , 18.8)** |
| **Australia** | **5756 (5277 , 6251)** | **18.9 (17.4 , 20.4)** | **29.4 (27 , 31.9)** | **3662 (3214 , 4043)** | **15.7 (14.5 , 17)** | **16.4 (14.7 , 18)** |
| **New Zealand** | **1217 (1101 , 1334)** | **21.3 (19.7 , 23.1)** | **32.8 (29.7 , 35.9)** | **887 (793 , 978)** | **18.2 (16.7 , 19.7)** | **21.1 (18.9 , 23.2)** |
| **High-income Asia Pacific** | **112706 (100921 , 121695)** | **34.8 (33 , 36.8)** | **53.9 (48.6 , 58)** | **22528 (18506 , 26147)** | **9.8 (8.8 , 10.8)** | **8.2 (7 , 9.2)** |
| **Brunei Darussalam** | **76 (64 , 90)** | **29.2 (26.9 , 31.6)** | **77.6 (66.3 , 90.1)** | **28 (22 , 35)** | **10.4 (8.4 , 12.7)** | **21.7 (17.7 , 26.9)** |
| **Japan** | **86874 (77172 , 94207)** | **33.9 (32 , 35.9)** | **51.8 (46.8 , 55.8)** | **18587 (14892 , 21868)** | **10 (8.9 , 11.2)** | **8.8 (7.5 , 10)** |
| **Singapore** | **1086 (976 , 1188)** | **28.3 (26.2 , 30.4)** | **30.9 (27.6 , 33.9)** | **253 (207 , 303)** | **8 (6.7 , 9.4)** | **6.2 (5.1 , 7.4)** |
| **Republic of Korea** | **24670 (22239 , 27169)** | **38.9 (36.2 , 41.5)** | **65.6 (58.8 , 72.5)** | **3660 (2946 , 4438)** | **9 (7.6 , 10.6)** | **7.2 (5.9 , 8.7)** |
| **Western Europe** | **231186 (215117 , 245521)** | **32.2 (30.5 , 33.9)** | **56.3 (52.7 , 59.6)** | **101573 (91899 , 109651)** | **17.8 (16.8 , 19)** | **20.9 (19.2 , 22.3)** |
| **Andorra** | **49 (38 , 62)** | **32 (29.5 , 34.6)** | **70.6 (54.7 , 87.8)** | **11 (8 , 15)** | **14.4 (12.3 , 16.7)** | **15.6 (10.6 , 21.7)** |
| **Austria** | **3786 (3511 , 4044)** | **31.4 (29.1 , 33.2)** | **48.1 (44.7 , 51.2)** | **1854 (1670 , 2040)** | **18.2 (16.9 , 19.6)** | **19.9 (18.2 , 21.7)** |
| **Belgium** | **6862 (6373 , 7381)** | **36 (33.5 , 38.1)** | **66.6 (62.2 , 71.6)** | **2696 (2428 , 2952)** | **18.2 (16.8 , 19.6)** | **22.7 (20.7 , 24.6)** |
| **Cyprus** | **499 (436 , 567)** | **38.3 (36 , 40.8)** | **54 (47.2 , 61.3)** | **124 (106 , 144)** | **12.6 (11.2 , 14.1)** | **12.1 (10.3 , 14)** |
| **Denmark** | **3249 (2989 , 3519)** | **33 (30.9 , 35.5)** | **59.3 (54.7 , 64.2)** | **2452 (2171 , 2721)** | **28.4 (26.4 , 30.4)** | **38.5 (34.5 , 42.6)** |
| **Finland** | **1973 (1796 , 2141)** | **25.7 (23.9 , 27.5)** | **35.1 (32 , 38)** | **924 (815 , 1026)** | **13.6 (12.4 , 14.9)** | **13.7 (12.3 , 15.3)** |
| **France** | **36025 (33084 , 38740)** | **31.7 (29.6 , 33.7)** | **61.4 (56.8 , 65.9)** | **12500 (10836 , 13905)** | **14.9 (13.6 , 16.4)** | **17.5 (15.6 , 19.2)** |
| **Germany** | **48093 (44471 , 51734)** | **32.1 (30.2 , 34.2)** | **56.3 (52.2 , 60.3)** | **21994 (19683 , 24041)** | **17.7 (16.5 , 19.1)** | **22.1 (20.1 , 23.8)** |
| **Greece** | **9184 (8438 , 9892)** | **43.9 (41.7 , 46.6)** | **84.8 (78.7 , 90.5)** | **2600 (2313 , 2852)** | **18.2 (16.8 , 19.7)** | **21.2 (19.3 , 23.1)** |
| **Iceland** | **103 (92 , 116)** | **27.1 (25.2 , 29.1)** | **38.9 (34.7 , 43.9)** | **62 (54 , 71)** | **22.9 (21 , 25)** | **21.3 (18.5 , 24.2)** |
| **Ireland** | **1621 (1476 , 1775)** | **29 (27 , 30.9)** | **45.8 (41.7 , 50.1)** | **1124 (996 , 1248)** | **23.4 (21.8 , 25.2)** | **27.8 (24.8 , 30.7)** |
| **Israel** | **2157 (1971 , 2343)** | **28.2 (26.1 , 30.1)** | **40.8 (37.3 , 44.2)** | **920 (809 , 1026)** | **13.2 (12 , 14.5)** | **14.3 (12.7 , 15.9)** |
| **Italy** | **34166 (31304 , 36766)** | **31.7 (30 , 33.6)** | **52.8 (48.7 , 56.5)** | **12388 (10750 , 13730)** | **14.7 (13.6 , 15.9)** | **16 (14.3 , 17.4)** |
| **Luxembourg** | **239 (208 , 274)** | **33.6 (30.8 , 36.4)** | **52 (45.3 , 59.5)** | **106 (88 , 125)** | **18.3 (16.1 , 20.3)** | **19.8 (16.6 , 23.1)** |
| **Malta** | **190 (169 , 213)** | **33 (30.8 , 35.1)** | **43.7 (38.9 , 49)** | **49 (42 , 58)** | **11.5 (10.3 , 12.7)** | **10.4 (8.9 , 12.2)** |
| **Monaco** | **44 (36 , 51)** | **35.6 (32.7 , 38.6)** | **99.9 (82 , 118)** | **20 (15 , 26)** | **20.1 (17.1 , 23.1)** | **41.2 (30.7 , 53.3)** |
| **Netherlands** | **10346 (9535 , 11171)** | **33.6 (31.7 , 35.8)** | **64.4 (59.5 , 69.7)** | **6362 (5724 , 6994)** | **24.7 (23 , 26.5)** | **34.4 (31.3 , 37.6)** |
| **Norway** | **1359 (1235 , 1477)** | **20 (18.4 , 21.6)** | **29.9 (27.2 , 32.4)** | **873 (768 , 986)** | **14.9 (13.4 , 16.5)** | **17.6 (15.6 , 19.6)** |
| **Portugal** | **5126 (4731 , 5531)** | **27.3 (25.4 , 29.2)** | **51.4 (47.6 , 55.2)** | **793 (684 , 905)** | **6.2 (5.4 , 7)** | **7 (6.2 , 8)** |
| **San Marino** | **19 (13 , 26)** | **27.4 (24.7 , 30.1)** | **62.9 (41.2 , 87.9)** | **6 (4 , 9)** | **12.9 (10.9 , 15)** | **18.4 (11.5 , 29.1)** |
| **Spain** | **28244 (25960 , 30332)** | **37.4 (35.1 , 39.5)** | **67.4 (62.2 , 72.1)** | **5874 (5250 , 6489)** | **11.7 (10.6 , 12.9)** | **13.2 (12 , 14.5)** |
| **Sweden** | **3005 (2707 , 3271)** | **21.5 (19.7 , 23.2)** | **28.9 (26.2 , 31.3)** | **2692 (2412 , 2948)** | **21.6 (19.9 , 23.1)** | **23.5 (21.3 , 25.5)** |
| **Switzerland** | **3327 (3041 , 3614)** | **29.6 (27.5 , 31.7)** | **41.4 (38 , 44.8)** | **1730 (1512 , 1915)** | **19.2 (17.7 , 20.7)** | **18.6 (16.6 , 20.3)** |
| **United Kingdom** | **31316 (29056 , 33408)** | **30.1 (28.4 , 32)** | **52.9 (49.3 , 56.4)** | **23329 (20926 , 25465)** | **25.4 (23.9 , 27)** | **33.2 (30.3 , 35.8)** |
| **Southern Latin America** | **17648 (16437 , 18896)** | **26.4 (24.7 , 28)** | **47.9 (44.5 , 51.3)** | **9601 (8639 , 10633)** | **16 (14.5 , 17.5)** | **20.8 (18.7 , 23.1)** |
| **Argentina** | **12929 (11929 , 13911)** | **28.8 (26.7 , 30.6)** | **54.6 (50.5 , 58.8)** | **6977 (6212 , 7787)** | **17.3 (15.6 , 19.2)** | **23.3 (20.8 , 25.9)** |
| **Chile** | **3005 (2708 , 3309)** | **18.3 (16.8 , 19.9)** | **27.7 (24.9 , 30.5)** | **2018 (1763 , 2278)** | **13.3 (11.7 , 14.9)** | **15.3 (13.4 , 17.3)** |
| **Uruguay** | **1713 (1584 , 1834)** | **31.5 (29.6 , 33.5)** | **75.9 (70.3 , 81.1)** | **607 (536 , 688)** | **13.1 (11.7 , 14.8)** | **21 (18.8 , 23.7)** |
| **Eastern Europe** | **103159 (88874 , 117489)** | **43.4 (41.8 , 45.2)** | **77.7 (66.8 , 88.3)** | **12876 (10789 , 15398)** | **6.4 (5.7 , 7.1)** | **6.5 (5.4 , 7.8)** |
| **Belarus** | **5033 (3891 , 6395)** | **44.5 (42 , 46.8)** | **82.7 (64.6 , 103.7)** | **479 (355 , 637)** | **5.7 (4.8 , 6.9)** | **5.4 (4 , 7.2)** |
| **Estonia** | **743 (582 , 929)** | **37.6 (35.2 , 40.9)** | **75 (58.8 , 93.8)** | **243 (186 , 310)** | **13.1 (11.8 , 14.3)** | **15.4 (11.6 , 19.7)** |
| **Latvia** | **1136 (901 , 1428)** | **38.9 (36.5 , 41.8)** | **78.3 (62.1 , 98.2)** | **228 (175 , 301)** | **8.5 (7.5 , 9.6)** | **10 (7.6 , 13.3)** |
| **Lithuania** | **1574 (1285 , 1909)** | **36.8 (34.6 , 39.7)** | **74.2 (60.6 , 89.9)** | **245 (191 , 307)** | **6.8 (5.9 , 7.8)** | **7.9 (6.2 , 10)** |
| **Republic of Moldova** | **1501 (1292 , 1727)** | **41.1 (38.8 , 43.5)** | **61 (52.5 , 69.8)** | **118 (90 , 151)** | **4.5 (3.7 , 5.6)** | **3.5 (2.7 , 4.5)** |
| **Russian Federation** | **68914 (56377 , 82564)** | **44.3 (42.5 , 46.1)** | **76.6 (62.9 , 91.4)** | **9473 (7669 , 11642)** | **6.7 (6 , 7.4)** | **6.9 (5.6 , 8.6)** |
| **Ukraine** | **24258 (19476 , 29918)** | **42 (39.9 , 44.1)** | **82.6 (66.4 , 101.6)** | **2090 (1574 , 2739)** | **5 (4.3 , 5.9)** | **4.9 (3.7 , 6.5)** |
| **Central Europe** | **79686 (69047 , 91595)** | **40.3 (38.6 , 42)** | **85.7 (74.5 , 98.5)** | **27533 (23720 , 31901)** | **18.6 (17.4 , 19.9)** | **23.5 (20.2 , 27.4)** |
| **Albania** | **1299 (952 , 1735)** | **42.7 (39.6 , 45.5)** | **63.3 (46.7 , 84.3)** | **169 (125 , 222)** | **10.1 (8.6 , 11.9)** | **7.3 (5.4 , 9.5)** |
| **Bosnia and Herzegovina** | **2559 (2000 , 3229)** | **46.4 (44.1 , 49)** | **95.4 (75.2 , 119.4)** | **781 (614 , 993)** | **19.7 (18 , 21.5)** | **23.7 (18.6 , 30.3)** |
| **Bulgaria** | **5386 (4286 , 6663)** | **41.9 (39.6 , 44.1)** | **87.8 (69.7 , 109.7)** | **1340 (1046 , 1717)** | **14.6 (13.2 , 16.3)** | **19.3 (15 , 25)** |
| **Croatia** | **3150 (2507 , 3970)** | **38.4 (36.1 , 41)** | **84.1 (67 , 105.9)** | **993 (789 , 1258)** | **17 (15.6 , 18.6)** | **20.5 (16.3 , 26.3)** |
| **Czechia** | **6161 (5034 , 7557)** | **35.8 (34 , 37.9)** | **65.5 (53.5 , 80.1)** | **2479 (2022 , 3071)** | **18.3 (16.9 , 19.9)** | **20.8 (16.9 , 25.7)** |
| **Hungary** | **7692 (6290 , 9317)** | **41.6 (39.5 , 43.8)** | **98.1 (80.1 , 118.5)** | **3709 (3042 , 4539)** | **23.5 (21.8 , 25.2)** | **35.3 (28.8 , 43.5)** |
| **Montenegro** | **512 (419 , 623)** | **50.9 (48.2 , 53.4)** | **113.4 (93.4 , 137.4)** | **175 (147 , 210)** | **27.4 (24.5 , 30.3)** | **32.4 (27.2 , 38.9)** |
| **North Macedonia** | **1475 (1150 , 1872)** | **44.6 (42.1 , 47.1)** | **93.7 (73.8 , 118)** | **375 (290 , 475)** | **17.7 (15.9 , 19.6)** | **22.4 (17.4 , 28.4)** |
| **Poland** | **28172 (22225 , 35240)** | **40.7 (38.9 , 42.6)** | **94.4 (74.6 , 118.1)** | **11113 (8713 , 13825)** | **21 (19.5 , 22.6)** | **28 (21.8 , 35)** |
| **Romania** | **12251 (10089 , 14769)** | **39.3 (37.3 , 41.3)** | **77.9 (64.1 , 94)** | **2721 (2119 , 3395)** | **12.7 (11.2 , 14.3)** | **14.3 (11.1 , 18.1)** |
| **Serbia** | **6969 (5525 , 8659)** | **42.9 (40.6 , 45.5)** | **94.7 (75 , 117.8)** | **2600 (2052 , 3300)** | **21.3 (19.6 , 23.3)** | **31.2 (24.4 , 39.7)** |
| **Slovakia** | **2857 (2230 , 3645)** | **36 (33.5 , 38.6)** | **72.3 (56.3 , 91.5)** | **649 (498 , 836)** | **10.9 (9.6 , 12.2)** | **12.7 (9.7 , 16.4)** |
| **Slovenia** | **1203 (937 , 1569)** | **32.4 (30.3 , 35.3)** | **63.4 (49.4 , 82.5)** | **428 (326 , 553)** | **16.3 (14.7 , 17.8)** | **18.9 (14.4 , 24.6)** |
| **Central Asia** | **17044 (15255 , 19051)** | **35.8 (34.1 , 37.6)** | **56.1 (50.5 , 62.3)** | **1217 (1024 , 1451)** | **3 (2.6 , 3.4)** | **2.9 (2.4 , 3.4)** |
| **Armenia** | **1583 (1323 , 1859)** | **48.2 (46 , 51)** | **89.1 (74.5 , 104.2)** | **63 (48 , 82)** | **2.5 (2 , 3.1)** | **2.6 (2 , 3.4)** |
| **Azerbaijan** | **2995 (2295 , 3840)** | **43.2 (39.6 , 46.1)** | **72.7 (56.8 , 92.1)** | **67 (46 , 95)** | **1.3 (1 , 1.8)** | **1.2 (0.9 , 1.7)** |
| **Georgia** | **2069 (1728 , 2430)** | **43.2 (41.1 , 45.9)** | **85 (71.1 , 99.6)** | **143 (111 , 182)** | **4 (3.4 , 4.8)** | **4.3 (3.3 , 5.5)** |
| **Kazakhstan** | **4457 (3830 , 5145)** | **39.8 (37.7 , 41.9)** | **64 (55 , 73.6)** | **410 (322 , 517)** | **4.2 (3.4 , 5)** | **3.9 (3.1 , 5)** |
| **Kyrgyzstan** | **791 (688 , 906)** | **36.2 (34.1 , 38.2)** | **41.5 (36.3 , 47.3)** | **84 (65 , 110)** | **4.3 (3.4 , 5.4)** | **3.2 (2.4 , 4.1)** |
| **Mongolia** | **1052 (791 , 1374)** | **30.8 (25.8 , 35.4)** | **123.1 (95.5 , 155.6)** | **128 (87 , 181)** | **5.1 (3.8 , 6.5)** | **11.6 (8 , 16.2)** |
| **Tajikistan** | **688 (542 , 915)** | **24.3 (22.2 , 27.5)** | **33.1 (26.7 , 43)** | **51 (37 , 69)** | **2 (1.6 , 2.6)** | **1.9 (1.4 , 2.6)** |
| **Turkmenistan** | **562 (444 , 709)** | **28.9 (26.8 , 30.8)** | **32.1 (25.5 , 40)** | **62 (45 , 84)** | **3.7 (2.9 , 4.5)** | **2.6 (1.9 , 3.5)** |
| **Uzbekistan** | **2847 (2327 , 3392)** | **25.9 (24 , 27.9)** | **32.2 (26.8 , 37.8)** | **208 (151 , 285)** | **1.8 (1.4 , 2.3)** | **1.6 (1.2 , 2.1)** |
| **Central Latin America** | **18349 (15278 , 21948)** | **15.9 (14.6 , 17.2)** | **17.6 (14.7 , 21)** | **7140 (5784 , 9051)** | **6.1 (5.3 , 7.2)** | **5.7 (4.6 , 7.2)** |
| **Colombia** | **3285 (2474 , 4313)** | **13.6 (12.1 , 15.1)** | **13.8 (10.4 , 18.1)** | **1916 (1469 , 2568)** | **7.5 (6.4 , 8.9)** | **6.7 (5.1 , 9)** |
| **Costa Rica** | **547 (423 , 698)** | **16.6 (15.2 , 18.2)** | **23.7 (18.4 , 30.2)** | **172 (130 , 227)** | **6.3 (5.4 , 7.3)** | **6.2 (4.7 , 8.2)** |
| **El Salvador** | **318 (231 , 420)** | **11.9 (10.4 , 13.4)** | **12.7 (9.2 , 16.9)** | **156 (106 , 225)** | **4.8 (3.7 , 6.1)** | **4.6 (3.1 , 6.7)** |
| **Guatemala** | **665 (497 , 862)** | **11.5 (9.9 , 13.2)** | **14.4 (10.9 , 18.5)** | **240 (159 , 347)** | **3.5 (2.6 , 4.7)** | **4.1 (2.7 , 5.8)** |
| **Honduras** | **740 (540 , 949)** | **21.5 (17.8 , 25.1)** | **27.6 (20.3 , 35.7)** | **301 (201 , 432)** | **7 (5 , 9.4)** | **9.7 (6.5 , 13.8)** |
| **Mexico** | **8553 (6828 , 10583)** | **16 (14.6 , 17.5)** | **16.7 (13.4 , 20.6)** | **2644 (2025 , 3409)** | **5 (4.2 , 5.9)** | **4.3 (3.3 , 5.6)** |
| **Nicaragua** | **323 (255 , 395)** | **14.7 (13.1 , 16.2)** | **18.7 (14.9 , 22.7)** | **73 (50 , 103)** | **3.3 (2.4 , 4.4)** | **2.9 (2.1 , 4.1)** |
| **Panama** | **303 (230 , 389)** | **14.2 (12.9 , 15.5)** | **15.4 (11.7 , 19.7)** | **96 (70 , 134)** | **5.2 (4.2 , 6.5)** | **4.4 (3.2 , 6.2)** |
| **Venezuela (Bolivarian Republic of)** | **3614 (2697 , 4724)** | **19.7 (17.8 , 21.9)** | **27.6 (20.6 , 35.7)** | **1543 (1089 , 2165)** | **9.4 (7.5 , 11.6)** | **10.1 (7.1 , 14)** |
| **Andean Latin America** | **3240 (2553 , 3968)** | **10.4 (9.2 , 11.7)** | **12.6 (9.9 , 15.4)** | **988 (739 , 1313)** | **2.9 (2.3 , 3.7)** | **3.4 (2.6 , 4.5)** |
| **Bolivia (Plurinational State of)** | **867 (598 , 1168)** | **13.3 (11 , 15.4)** | **22.2 (15.5 , 29.7)** | **232 (147 , 350)** | **3.1 (2.2 , 4.3)** | **5.1 (3.3 , 7.6)** |
| **Ecuador** | **1148 (896 , 1475)** | **13.4 (11.9 , 14.9)** | **17.1 (13.4 , 21.9)** | **399 (292 , 531)** | **4.4 (3.6 , 5.6)** | **5.2 (3.8 , 7)** |
| **Peru** | **1225 (861 , 1685)** | **7.6 (6.4 , 8.7)** | **8.1 (5.7 , 11.2)** | **357 (239 , 512)** | **2.1 (1.6 , 2.7)** | **2.1 (1.4 , 3.1)** |
| **Caribbean** | **9973 (8425 , 11636)** | **26 (24 , 28.2)** | **41.5 (35.1 , 48.5)** | **3832 (3135 , 4574)** | **12.3 (10.7 , 13.9)** | **13.8 (11.3 , 16.6)** |
| **Antigua and Barbuda** | **10 (8 , 11)** | **12.9 (11.3 , 14.8)** | **21.3 (17.5 , 25.7)** | **4 (3 , 5)** | **5.6 (4.4 , 7.1)** | **6.7 (5.2 , 8.8)** |
| **Barbados** | **53 (42 , 64)** | **12.1 (10.7 , 13.7)** | **24 (19.3 , 29.3)** | **9 (7 , 12)** | **2.3 (1.8 , 2.9)** | **3.4 (2.5 , 4.6)** |
| **Belize** | **33 (28 , 39)** | **19.6 (17.9 , 21.5)** | **25.1 (21.3 , 29.2)** | **7 (5 , 9)** | **5.3 (4 , 7)** | **5.1 (3.8 , 6.9)** |
| **Bermuda** | **24 (20 , 28)** | **21.8 (19.6 , 24.1)** | **40.9 (34.3 , 48.9)** | **7 (5 , 9)** | **9.9 (8.4 , 11.7)** | **9.1 (7.1 , 11.8)** |
| **Bahamas** | **48 (38 , 59)** | **15 (13.1 , 16.8)** | **28.1 (22.5 , 34.6)** | **11 (8 , 15)** | **4.2 (3.2 , 5.4)** | **5.2 (3.8 , 6.9)** |
| **Cuba** | **6032 (4889 , 7346)** | **37.4 (35.2 , 41)** | **67.2 (54.5 , 81.9)** | **2244 (1755 , 2765)** | **19.3 (17.1 , 21.9)** | **22.3 (17.4 , 27.4)** |
| **Dominica** | **14 (11 , 18)** | **12.5 (10.8 , 14.4)** | **32.6 (26 , 40.5)** | **3 (2 , 4)** | **4.6 (3.7 , 5.8)** | **6.7 (5 , 9)** |
| **Dominican Republic** | **1330 (983 , 1764)** | **21.8 (19.5 , 23.9)** | **31.6 (23.6 , 41.7)** | **731 (532 , 983)** | **15.6 (12.9 , 18.4)** | **15.6 (11.4 , 20.9)** |
| **Grenada** | **15 (13 , 17)** | **14.7 (13 , 16.8)** | **29.9 (25.8 , 34)** | **4 (3 , 5)** | **4.6 (3.7 , 5.9)** | **6.6 (5.2 , 8.6)** |
| **Guyana** | **51 (39 , 67)** | **13.6 (11.9 , 15.2)** | **18 (13.7 , 23.4)** | **18 (12 , 26)** | **4.5 (3.4 , 5.9)** | **5.4 (3.8 , 7.6)** |
| **Haiti** | **468 (297 , 686)** | **9.5 (7.9 , 11.5)** | **15 (9.7 , 21.9)** | **166 (93 , 261)** | **2.9 (2.1 , 4.2)** | **4.4 (2.5 , 6.8)** |
| **Jamaica** | **501 (394 , 632)** | **20.6 (18.4 , 24)** | **35.5 (27.9 , 44.8)** | **127 (92 , 169)** | **6.7 (5.3 , 8.3)** | **8.2 (5.9 , 10.9)** |
| **Puerto Rico** | **689 (523 , 885)** | **18.1 (16 , 20.2)** | **21.4 (16.1 , 27.6)** | **269 (201 , 351)** | **8.6 (7.3 , 10)** | **6.5 (4.9 , 8.6)** |
| **Saint Kitts and Nevis** | **7 (6 , 8)** | **11.8 (10.4 , 13.3)** | **22.3 (18.5 , 26.3)** | **2 (1 , 2)** | **4 (3 , 5.1)** | **5 (3.7 , 6.7)** |
| **Saint Lucia** | **29 (24 , 35)** | **16 (14.1 , 17.7)** | **29.6 (24.7 , 35.5)** | **7 (5 , 9)** | **5.5 (4.3 , 6.8)** | **5.8 (4.4 , 7.6)** |
| **Saint Vincent and the Grenadines** | **16 (13 , 19)** | **12.9 (11.2 , 14.8)** | **22.8 (19.2 , 27.2)** | **3 (3 , 5)** | **4 (3 , 5.4)** | **5.3 (3.9 , 7.3)** |
| **Suriname** | **87 (71 , 107)** | **23.2 (20.9 , 25.8)** | **32.5 (26.5 , 39.7)** | **30 (22 , 39)** | **8.9 (7.1 , 11.1)** | **9.3 (6.9 , 12.2)** |
| **Trinidad and Tobago** | **198 (145 , 263)** | **18 (16.1 , 20.3)** | **22.4 (16.5 , 29.6)** | **50 (35 , 72)** | **5.5 (4.4 , 7)** | **5.2 (3.6 , 7.5)** |
| **United States Virgin Islands** | **31 (26 , 37)** | **15.2 (13.2 , 17.3)** | **36.8 (30.4 , 43.5)** | **11 (9 , 14)** | **9.1 (7.5 , 10.8)** | **10.9 (8.4 , 13.7)** |
| **Tropical Latin America** | **35135 (32301 , 37758)** | **24.1 (22.6 , 25.6)** | **33.1 (30.3 , 35.6)** | **18158 (16049 , 20343)** | **14.4 (13 , 15.9)** | **13.6 (12 , 15.3)** |
| **Brazil** | **34144 (31386 , 36687)** | **24 (22.4 , 25.4)** | **32.9 (30.2 , 35.4)** | **17829 (15783 , 19966)** | **14.4 (13.1 , 15.9)** | **13.7 (12.1 , 15.3)** |
| **Paraguay** | **991 (760 , 1283)** | **29.6 (27.1 , 32.3)** | **39.9 (30.9 , 51.3)** | **329 (240 , 451)** | **11.7 (9.5 , 14.6)** | **11.5 (8.4 , 15.7)** |
| **East Asia** | **831218 (666328 , 1018022)** | **46 (44.2 , 47.7)** | **88.4 (71.6 , 107.1)** | **93255 (74497 , 115446)** | **9.3 (8.3 , 10.3)** | **8.9 (7.1 , 10.9)** |
| **China** | **809120 (643524 , 995553)** | **46.3 (44.4 , 48)** | **89.3 (71.9 , 108.8)** | **91242 (72424 , 113479)** | **9.4 (8.5 , 10.4)** | **9 (7.2 , 11.2)** |
| **Democratic People's Republic of Korea** | **9321 (7430 , 11431)** | **41.2 (37.9 , 44.6)** | **72.2 (58 , 87.3)** | **1146 (812 , 1545)** | **6.3 (4.8 , 8)** | **6 (4.3 , 8.1)** |
| **Taiwan (Province of China)** | **12777 (9963 , 16634)** | **36.6 (34.8 , 38.7)** | **68.8 (53.7 , 89.4)** | **868 (621 , 1182)** | **3.9 (3.1 , 4.9)** | **4.2 (3 , 5.7)** |
| **Southeast Asia** | **124705 (108070 , 143332)** | **35.6 (33.1 , 38)** | **48.2 (42.1 , 55.3)** | **16151 (12900 , 20024)** | **5.1 (4.5 , 5.9)** | **5.4 (4.3 , 6.6)** |
| **Cambodia** | **3061 (2385 , 3628)** | **41 (38.1 , 44.1)** | **70.6 (56.1 , 83.8)** | **436 (326 , 558)** | **6.2 (5.1 , 7.4)** | **6.9 (5.2 , 8.7)** |
| **Indonesia** | **43425 (33951 , 54623)** | **37.3 (33.7 , 40.4)** | **47.7 (38.2 , 58.7)** | **4415 (2742 , 6416)** | **3.9 (2.9 , 4.9)** | **4.5 (2.8 , 6.4)** |
| **Lao People's Democratic Republic** | **950 (717 , 1189)** | **37.3 (34.5 , 40.3)** | **51 (39.3 , 62.6)** | **128 (89 , 182)** | **5.3 (4.2 , 6.6)** | **6.2 (4.4 , 8.6)** |
| **Malaysia** | **5344 (4216 , 6676)** | **32.2 (29.8 , 34.6)** | **42.8 (34.1 , 53.3)** | **437 (320 , 590)** | **3 (2.4 , 3.7)** | **3.8 (2.8 , 5.1)** |
| **Maldives** | **37 (30 , 44)** | **30.6 (28.4 , 32.8)** | **26.4 (21.3 , 31.4)** | **4 (3 , 6)** | **4.7 (3.7 , 5.9)** | **3.7 (2.7 , 5)** |
| **Mauritius** | **217 (176 , 267)** | **28.7 (26.8 , 30.6)** | **28.7 (23.6 , 35.1)** | **22 (17 , 29)** | **2.9 (2.3 , 3.6)** | **2.4 (1.7 , 3.1)** |
| **Myanmar** | **7936 (6063 , 10645)** | **30.4 (26.5 , 34.8)** | **43.5 (33.5 , 57.4)** | **2681 (2086 , 3674)** | **10.1 (8.3 , 11.8)** | **11.2 (8.8 , 15.1)** |
| **Philippines** | **13405 (10147 , 17449)** | **32.8 (30.5 , 35)** | **39.6 (30.3 , 51.4)** | **3237 (2491 , 4214)** | **8 (7.1 , 9)** | **8.7 (6.8 , 11.2)** |
| **Sri Lanka** | **2422 (1777 , 3280)** | **25.8 (23.6 , 27.9)** | **22.3 (16.5 , 29.8)** | **218 (154 , 303)** | **2.3 (1.8 , 2.9)** | **1.6 (1.1 , 2.2)** |
| **Seychelles** | **34 (29 , 40)** | **31.2 (29.1 , 33.9)** | **71.7 (61.8 , 83.3)** | **3 (2 , 4)** | **4.5 (3.5 , 5.8)** | **5.9 (4.3 , 7.9)** |
| **Thailand** | **22126 (15832 , 29349)** | **33.9 (30.2 , 37.3)** | **48.6 (35.1 , 63.9)** | **2866 (2107 , 3799)** | **5.7 (4.8 , 6.8)** | **5.1 (3.8 , 6.8)** |
| **Timor-Leste** | **150 (101 , 191)** | **34.6 (30.6 , 38.8)** | **39.3 (27 , 49.2)** | **13 (9 , 20)** | **3.4 (2.3 , 5)** | **3.5 (2.3 , 5.1)** |
| **Viet Nam** | **25436 (20138 , 31092)** | **39.9 (37.2 , 43.1)** | **65.8 (53 , 79)** | **1670 (1191 , 2284)** | **3.4 (2.7 , 4.3)** | **3.4 (2.4 , 4.5)** |
| **Oceania** | **1042 (789 , 1408)** | **24.8 (21.8 , 28.2)** | **32 (25 , 42.3)** | **372 (266 , 501)** | **8.1 (6.4 , 10.2)** | **10.7 (8 , 14.2)** |
| **American Samoa** | **10 (8 , 11)** | **27.3 (24.6 , 30)** | **45.6 (39.3 , 51.8)** | **4 (3 , 5)** | **10.8 (9 , 12.9)** | **14.2 (10.9 , 18.4)** |
| **Cook Islands** | **6 (5 , 7)** | **27.9 (25 , 31.1)** | **46.8 (39.5 , 55.5)** | **1 (1 , 1)** | **9.8 (7.8 , 11.9)** | **8.9 (6.4 , 11.7)** |
| **Micronesia (Federated States of)** | **18 (11 , 26)** | **32.3 (27.6 , 37.5)** | **59.1 (40.1 , 84.6)** | **8 (5 , 12)** | **14.5 (11.2 , 18.2)** | **22.1 (14.5 , 30.7)** |
| **Fiji** | **75 (58 , 96)** | **21.9 (19.3 , 24.5)** | **24.6 (19.6 , 30.9)** | **30 (19 , 44)** | **6.4 (4.6 , 8.4)** | **7.4 (4.9 , 10.6)** |
| **Guam** | **36 (29 , 44)** | **29 (26.3 , 32)** | **40.1 (32.7 , 48.5)** | **12 (9 , 15)** | **14.6 (12.5 , 17.1)** | **12.4 (9.8 , 15.7)** |
| **Kiribati** | **19 (14 , 24)** | **34.6 (32 , 37.1)** | **71 (55.1 , 88.2)** | **17 (11 , 24)** | **23 (17 , 29.4)** | **43 (30 , 60.5)** |
| **Marshall Islands** | **7 (5 , 11)** | **26.4 (21 , 31.9)** | **44.3 (30.6 , 64.6)** | **2 (1 , 3)** | **6.7 (4.9 , 8.9)** | **10.5 (6.8 , 15)** |
| **Nauru** | **1 (1 , 1)** | **25.7 (21.8 , 29.4)** | **61.7 (43.9 , 81.1)** | **1 (0 , 1)** | **17.1 (13.5 , 20.7)** | **26.7 (17.5 , 38.2)** |
| **Niue** | **0 (0 , 1)** | **28.2 (25 , 31.9)** | **45.7 (38.4 , 53.7)** | **0 (0 , 0)** | **10.9 (8.8 , 13.3)** | **12.9 (9 , 17.6)** |
| **Northern Mariana Islands** | **17 (14 , 19)** | **34.9 (31.7 , 38.3)** | **70.8 (61.6 , 79.7)** | **4 (3 , 5)** | **11.8 (9 , 14.7)** | **14.4 (10.6 , 19.1)** |
| **Palau** | **5 (4 , 6)** | **27 (24 , 30.3)** | **46.8 (38 , 58.7)** | **2 (2 , 3)** | **12 (9.4 , 15.3)** | **20.5 (14.6 , 28.9)** |
| **Papua New Guinea** | **635 (443 , 925)** | **23.4 (19.5 , 27.8)** | **28.3 (20.1 , 40.4)** | **228 (147 , 329)** | **7.8 (5.7 , 9.9)** | **10.1 (6.8 , 14.3)** |
| **Samoa** | **20 (17 , 25)** | **28.3 (25.5 , 30.7)** | **30.3 (25 , 36.5)** | **9 (6 , 12)** | **10.4 (8.6 , 12.7)** | **11.7 (8.6 , 15.8)** |
| **Solomon Islands** | **87 (56 , 132)** | **30.8 (26.1 , 36.3)** | **60.4 (41.5 , 89.3)** | **29 (19 , 44)** | **7.9 (5.9 , 10.2)** | **17.8 (12.3 , 24.8)** |
| **Tokelau** | **0 (0 , 0)** | **29.9 (26.2 , 33.5)** | **33.6 (25.5 , 44.2)** | **0 (0 , 0)** | **10.7 (8.2 , 13.6)** | **14.1 (9.3 , 20.5)** |
| **Tonga** | **23 (18 , 27)** | **30.7 (27.3 , 33.9)** | **65.6 (53.7 , 78.8)** | **4 (3 , 6)** | **8.3 (6.6 , 10.4)** | **10.5 (7.5 , 14.1)** |
| **Tuvalu** | **2 (2 , 3)** | **30.4 (26.8 , 34.6)** | **45.1 (34.5 , 59.8)** | **1 (1 , 1)** | **11.9 (9.6 , 14.7)** | **15.1 (10.5 , 20.9)** |
| **Vanuatu** | **32 (22 , 44)** | **24.4 (21.1 , 28.6)** | **37.7 (27.2 , 52)** | **3 (2 , 4)** | **2.9 (2.2 , 3.9)** | **3.7 (2.5 , 5.4)** |
| **North Africa and Middle East** | **83744 (73854 , 95266)** | **34.2 (32.4 , 36.2)** | **40.4 (35.6 , 45.9)** | **8234 (7030 , 9643)** | **4.7 (4.2 , 5.2)** | **4.1 (3.5 , 4.7)** |
| **Afghanistan** | **1527 (1085 , 2152)** | **17 (14.3 , 20.1)** | **28.5 (20.7 , 39)** | **193 (125 , 285)** | **1.6 (1.1 , 2.1)** | **2.8 (1.9 , 4)** |
| **Algeria** | **4053 (3197 , 5106)** | **31.4 (29.2 , 33.8)** | **25.7 (20.4 , 32.3)** | **229 (169 , 310)** | **2.1 (1.6 , 2.7)** | **1.6 (1.2 , 2.2)** |
| **Bahrain** | **137 (105 , 176)** | **28.6 (25.7 , 31.8)** | **40.3 (31.8 , 50)** | **17 (13 , 23)** | **5.1 (4 , 6.4)** | **6.5 (4.9 , 8.7)** |
| **Egypt** | **11602 (8305 , 15846)** | **31.8 (28.1 , 35.4)** | **33.6 (24.4 , 45.4)** | **241 (150 , 360)** | **1.1 (0.8 , 1.5)** | **1 (0.7 , 1.5)** |
| **Iran (Islamic Republic of)** | **9971 (9103 , 11030)** | **25.6 (23.7 , 27.9)** | **29.1 (26.5 , 32.2)** | **1140 (938 , 1344)** | **4.1 (3.4 , 4.8)** | **3.2 (2.6 , 3.8)** |
| **Iraq** | **4794 (3669 , 5742)** | **36.7 (34.5 , 38.9)** | **49 (38.5 , 57.9)** | **611 (456 , 791)** | **5.4 (4.5 , 6.3)** | **5.6 (4.2 , 7.2)** |
| **Jordan** | **1138 (880 , 1415)** | **36.4 (34.1 , 38.5)** | **37.2 (29 , 46.1)** | **168 (132 , 216)** | **6.7 (5.7 , 7.7)** | **6.2 (4.9 , 7.9)** |
| **Kuwait** | **284 (221 , 351)** | **27.5 (25.5 , 29.6)** | **22.8 (18.1 , 28)** | **20 (14 , 26)** | **3.2 (2.5 , 3.9)** | **2.3 (1.6 , 3.1)** |
| **Lebanon** | **1565 (1284 , 2051)** | **39.5 (36 , 43.5)** | **66.9 (54.9 , 87.4)** | **679 (543 , 915)** | **19.2 (16.9 , 21.2)** | **23.8 (19 , 32)** |
| **Libya** | **1169 (891 , 1475)** | **37.8 (35 , 40.3)** | **47.9 (36.7 , 59.9)** | **18 (12 , 26)** | **0.8 (0.6 , 1)** | **0.8 (0.5 , 1.1)** |
| **Morocco** | **5054 (3645 , 6352)** | **34.7 (32.4 , 37.2)** | **32.8 (23.8 , 40.8)** | **153 (105 , 216)** | **1.1 (0.9 , 1.5)** | **1 (0.7 , 1.3)** |
| **Palestine** | **547 (464 , 638)** | **35.4 (32.9 , 37.8)** | **56.6 (48.4 , 65.3)** | **38 (28 , 49)** | **2.8 (2.2 , 3.4)** | **3.5 (2.6 , 4.5)** |
| **Oman** | **119 (95 , 153)** | **15.3 (13.7 , 17)** | **16.9 (14.1 , 20.3)** | **10 (7 , 13)** | **1.7 (1.3 , 2.2)** | **1.7 (1.3 , 2.3)** |
| **Qatar** | **131 (94 , 176)** | **23.8 (21.1 , 26.5)** | **34.1 (26 , 43)** | **4 (2 , 5)** | **1.3 (1 , 1.8)** | **2.4 (1.7 , 3.4)** |
| **Saudi Arabia** | **1573 (1205 , 1969)** | **21.4 (19 , 23.7)** | **16 (12.9 , 19.7)** | **102 (71 , 144)** | **1.8 (1.3 , 2.3)** | **1.7 (1.2 , 2.3)** |
| **Sudan** | **2378 (1573 , 3297)** | **23.6 (20.5 , 27.6)** | **25.7 (17.2 , 35.1)** | **152 (100 , 214)** | **2.2 (1.6 , 2.9)** | **1.9 (1.2 , 2.6)** |
| **Syrian Arab Republic** | **1720 (1274 , 2292)** | **34.8 (31.1 , 38.1)** | **28.4 (21.2 , 37.2)** | **194 (129 , 274)** | **5 (3.8 , 6.6)** | **3.5 (2.5 , 4.8)** |
| **Tunisia** | **3041 (2206 , 4202)** | **49.5 (46.8 , 52.1)** | **51.4 (37.6 , 70.3)** | **107 (75 , 154)** | **2.7 (2.1 , 3.4)** | **1.7 (1.2 , 2.4)** |
| **Turkey** | **29958 (23839 , 37112)** | **45.4 (43.2 , 47.7)** | **72.8 (58.2 , 89.8)** | **3704 (2877 , 4657)** | **9.5 (8.5 , 10.7)** | **7.9 (6.1 , 9.9)** |
| **United Arab Emirates** | **791 (560 , 1084)** | **19.7 (16.8 , 22.5)** | **36.2 (28.5 , 44.9)** | **53 (36 , 73)** | **3.7 (2.8 , 4.7)** | **6.1 (4.3 , 8.4)** |
| **Yemen** | **2103 (1553 , 2833)** | **30 (26.9 , 34)** | **35.1 (26.2 , 47)** | **393 (285 , 532)** | **6.9 (5.7 , 8.3)** | **6 (4.4 , 8)** |
| **South Asia** | **168176 (141799 , 197359)** | **26.6 (24.9 , 28.3)** | **25.5 (21.6 , 29.9)** | **21326 (16608 , 26973)** | **3.5 (3 , 4.1)** | **3.2 (2.5 , 4)** |
| **Bangladesh** | **16713 (12137 , 22999)** | **27.5 (23.5 , 32.3)** | **25.3 (18.6 , 34.7)** | **981 (665 , 1427)** | **2.2 (1.7 , 2.8)** | **1.7 (1.2 , 2.4)** |
| **Bhutan** | **48 (35 , 61)** | **18.6 (15.7 , 21.8)** | **17.9 (13.3 , 22.8)** | **8 (6 , 11)** | **3.7 (2.7 , 4.8)** | **3 (2.1 , 4.2)** |
| **India** | **122450 (98581 , 149636)** | **26 (24.3 , 27.7)** | **23.2 (18.9 , 28.2)** | **16531 (12365 , 22002)** | **3.6 (3 , 4.3)** | **3 (2.3 , 4)** |
| **Nepal** | **2382 (1845 , 2892)** | **20.8 (18.3 , 23.4)** | **24.3 (18.8 , 29.3)** | **1098 (800 , 1431)** | **10.8 (8.8 , 13)** | **10.4 (7.5 , 13.5)** |
| **Pakistan** | **26584 (20269 , 34972)** | **30.1 (27.9 , 32.2)** | **49.3 (37.7 , 64)** | **2708 (1994 , 3700)** | **3 (2.4 , 3.7)** | **5.2 (3.8 , 7.1)** |
| **Southern Sub-Saharan Africa** | **9265 (8292 , 10312)** | **24.8 (23.1 , 26.5)** | **41.3 (37.1 , 45.8)** | **3198 (2690 , 3855)** | **8.7 (7.4 , 10.2)** | **10.2 (8.6 , 12.3)** |
| **Botswana** | **277 (207 , 347)** | **26.9 (24.3 , 29.5)** | **52.7 (40.9 , 64.4)** | **79 (50 , 120)** | **7.5 (5.8 , 9.7)** | **11.1 (7.3 , 16.4)** |
| **Lesotho** | **319 (248 , 411)** | **27.7 (24.8 , 31.2)** | **64.7 (51.6 , 81.8)** | **56 (30 , 101)** | **5.3 (3.4 , 8.1)** | **8.8 (4.7 , 15.3)** |
| **Namibia** | **105 (84 , 129)** | **13.6 (11.7 , 15.5)** | **19.9 (16.2 , 24.2)** | **72 (50 , 101)** | **8.7 (6.8 , 11.1)** | **9.6 (6.7 , 13.3)** |
| **South Africa** | **7192 (6332 , 8196)** | **25.1 (23.3 , 26.9)** | **40.1 (35.3 , 45.3)** | **2561 (2130 , 3067)** | **9.8 (8.3 , 11.5)** | **10.2 (8.5 , 12.2)** |
| **Eswatini** | **61 (43 , 83)** | **11.1 (9 , 13.4)** | **29.2 (20.6 , 38.4)** | **21 (12 , 33)** | **5 (3.5 , 7.2)** | **7.3 (4.2 , 11.4)** |
| **Zimbabwe** | **1311 (1067 , 1542)** | **25 (22.3 , 28)** | **48.8 (40.8 , 57.2)** | **408 (270 , 586)** | **5.7 (4.3 , 7.5)** | **11.7 (7.8 , 16.8)** |
| **Western Sub-Saharan Africa** | **10188 (8420 , 12214)** | **10.3 (9 , 11.8)** | **12.5 (10.4 , 14.9)** | **1407 (1002 , 1986)** | **1.5 (1.1 , 2)** | **1.5 (1.1 , 2)** |
| **Benin** | **334 (254 , 434)** | **12.3 (10.7 , 14)** | **16.9 (12.9 , 21.6)** | **44 (29 , 65)** | **1.8 (1.2 , 2.4)** | **1.7 (1.1 , 2.5)** |
| **Burkina Faso** | **469 (356 , 606)** | **9.6 (8.1 , 11.2)** | **12 (9.3 , 15.4)** | **36 (21 , 56)** | **0.7 (0.4 , 1)** | **0.7 (0.4 , 1.1)** |
| **Cameroon** | **1073 (782 , 1451)** | **14.6 (12.5 , 16.9)** | **19.8 (14.7 , 26.2)** | **79 (48 , 123)** | **1.1 (0.8 , 1.6)** | **1.5 (0.9 , 2.3)** |
| **Cabo Verde** | **51 (41 , 60)** | **11.8 (9.9 , 13.8)** | **29.8 (23.7 , 35.5)** | **8 (6 , 11)** | **2.9 (2.2 , 3.6)** | **3.5 (2.6 , 4.7)** |
| **Chad** | **470 (346 , 629)** | **13.4 (11.2 , 15.9)** | **17.1 (12.7 , 23)** | **55 (32 , 88)** | **2 (1.3 , 3)** | **2.3 (1.4 , 3.5)** |
| **Côte d’Ivoire** | **1113 (832 , 1435)** | **16.8 (14.9 , 18.8)** | **21.7 (16.6 , 27.5)** | **184 (120 , 282)** | **3.7 (2.6 , 5.2)** | **4.1 (2.7 , 6.1)** |
| **Gambia** | **93 (63 , 128)** | **15.3 (11.2 , 19.5)** | **20.8 (14.6 , 28)** | **5 (3 , 8)** | **1.1 (0.7 , 1.6)** | **1.1 (0.7 , 1.6)** |
| **Ghana** | **720 (570 , 899)** | **8.3 (7.1 , 9.6)** | **11.9 (9.5 , 14.6)** | **159 (103 , 239)** | **1.6 (1.1 , 2.3)** | **1.9 (1.3 , 2.8)** |
| **Guinea** | **797 (570 , 1054)** | **16.7 (13.2 , 20.5)** | **29.9 (21.7 , 39.2)** | **76 (46 , 120)** | **2.1 (1.3 , 3.2)** | **2.8 (1.8 , 4.4)** |
| **Guinea-Bissau** | **46 (33 , 62)** | **9.7 (8.1 , 11.5)** | **15.1 (11 , 20.1)** | **6 (3 , 9)** | **1.1 (0.7 , 1.7)** | **1.4 (0.8 , 2.3)** |
| **Liberia** | **122 (86 , 166)** | **11.7 (10 , 13.3)** | **13.1 (9.4 , 17.6)** | **19 (12 , 30)** | **1.9 (1.3 , 2.7)** | **1.9 (1.2 , 2.9)** |
| **Mali** | **682 (489 , 905)** | **13.2 (10.7 , 15.6)** | **17 (12.1 , 22.2)** | **73 (44 , 113)** | **1.8 (1.1 , 2.6)** | **1.7 (1.1 , 2.6)** |
| **Mauritania** | **157 (110 , 210)** | **15.3 (13 , 17.6)** | **15.7 (11.2 , 20.7)** | **28 (17 , 42)** | **2.6 (1.9 , 3.6)** | **2.5 (1.6 , 3.7)** |
| **Niger** | **287 (194 , 399)** | **8.2 (6.8 , 9.8)** | **9.1 (6.3 , 12.4)** | **31 (17 , 53)** | **0.9 (0.5 , 1.4)** | **0.7 (0.4 , 1.2)** |
| **Nigeria** | **2442 (1743 , 3432)** | **6.1 (4.9 , 7.8)** | **6.7 (4.9 , 9.5)** | **426 (258 , 671)** | **1 (0.7 , 1.5)** | **1 (0.6 , 1.5)** |
| **Sao Tome and Principe** | **9 (7 , 11)** | **12.1 (10.5 , 13.9)** | **20 (16.1 , 24.3)** | **1 (1 , 2)** | **2 (1.3 , 3)** | **2.7 (1.7 , 4.1)** |
| **Senegal** | **655 (493 , 853)** | **15.3 (13.5 , 17.4)** | **19 (14.6 , 24.5)** | **39 (24 , 62)** | **1 (0.7 , 1.4)** | **1 (0.6 , 1.5)** |
| **Sierra Leone** | **330 (238 , 441)** | **17 (15.1 , 19)** | **20.1 (14.7 , 26.3)** | **59 (35 , 96)** | **3.2 (2 , 4.7)** | **3.1 (1.9 , 4.9)** |
| **Togo** | **337 (251 , 445)** | **18.4 (16.2 , 20.7)** | **25.2 (19.4 , 32.4)** | **77 (51 , 115)** | **3.8 (2.7 , 5.4)** | **3.9 (2.6 , 5.8)** |
| **Eastern Sub-Saharan Africa** | **11312 (9324 , 13965)** | **12.3 (11 , 13.8)** | **16 (13.3 , 19.5)** | **3167 (2217 , 4460)** | **3.2 (2.4 , 4.3)** | **4 (2.9 , 5.4)** |
| **Burundi** | **345 (245 , 471)** | **12.7 (11 , 14.5)** | **15.5 (11.3 , 20.7)** | **65 (35 , 109)** | **2.5 (1.6 , 3.9)** | **2.7 (1.6 , 4.5)** |
| **Comoros** | **41 (31 , 54)** | **17.1 (14.8 , 19.6)** | **20.3 (15.4 , 25.8)** | **7 (4 , 11)** | **2.3 (1.6 , 3.4)** | **2.8 (1.7 , 4.3)** |
| **Djibouti** | **92 (62 , 138)** | **22.4 (18.8 , 26.4)** | **32 (23.1 , 46.3)** | **12 (7 , 20)** | **3.5 (2.4 , 5.1)** | **4.9 (3.1 , 7.4)** |
| **Eritrea** | **176 (123 , 232)** | **11.6 (9.7 , 13.7)** | **15.2 (10.9 , 19.8)** | **10 (5 , 17)** | **0.5 (0.3 , 0.7)** | **0.6 (0.4 , 1.1)** |
| **Ethiopia** | **1145 (793 , 1601)** | **5.9 (4.6 , 7.3)** | **6 (4.2 , 8.4)** | **107 (56 , 183)** | **0.5 (0.3 , 0.8)** | **0.5 (0.3 , 0.8)** |
| **Kenya** | **1608 (1266 , 1964)** | **14.3 (12.8 , 15.9)** | **17 (13.6 , 20.4)** | **280 (188 , 411)** | **2.3 (1.8 , 3.1)** | **2.7 (1.9 , 4)** |
| **Madagascar** | **538 (385 , 731)** | **11.2 (9.6 , 12.8)** | **10.9 (7.9 , 14.6)** | **105 (60 , 179)** | **1.7 (1 , 2.6)** | **1.9 (1.1 , 3)** |
| **Malawi** | **793 (594 , 1026)** | **16 (13.3 , 19)** | **28.1 (21.8 , 35)** | **150 (97 , 229)** | **2.9 (2 , 4.2)** | **3.8 (2.5 , 5.6)** |
| **Mozambique** | **835 (651 , 1062)** | **13.7 (11.9 , 15.6)** | **19.9 (16 , 24.6)** | **222 (122 , 381)** | **3 (1.8 , 4.5)** | **3.7 (2.1 , 6.1)** |
| **Rwanda** | **597 (440 , 808)** | **18.7 (16 , 21.7)** | **27.6 (20.7 , 36.5)** | **397 (287 , 547)** | **10 (7.7 , 12.9)** | **12.9 (9.6 , 17.3)** |
| **Somalia** | **457 (286 , 643)** | **12.6 (10 , 15.3)** | **17.7 (11.3 , 24.7)** | **109 (52 , 196)** | **2.5 (1.4 , 3.9)** | **3 (1.5 , 5.1)** |
| **South Sudan** | **314 (216 , 440)** | **14.1 (11.4 , 16.7)** | **17.2 (12 , 23.4)** | **41 (24 , 69)** | **2.2 (1.4 , 3.2)** | **2.5 (1.5 , 3.9)** |
| **United Republic of Tanzania** | **2642 (1928 , 3658)** | **16.9 (14.6 , 19.4)** | **24.4 (18.3 , 33.3)** | **1097 (724 , 1626)** | **6.9 (4.9 , 9.8)** | **8.8 (6.1 , 12.5)** |
| **Uganda** | **1037 (808 , 1293)** | **9.4 (7.9 , 10.9)** | **18.1 (14.4 , 22.1)** | **359 (235 , 518)** | **3.4 (2.4 , 4.7)** | **5 (3.4 , 7.1)** |
| **Zambia** | **685 (519 , 865)** | **13.9 (11.9 , 16)** | **24.1 (18.7 , 29.8)** | **203 (138 , 292)** | **4.2 (3 , 5.5)** | **7.1 (5 , 10)** |
| **Central Sub-Saharan Africa** | **4458 (3187 , 6491)** | **15.9 (13.4 , 19.8)** | **20.5 (14.8 , 28.8)** | **597 (390 , 914)** | **2 (1.5 , 2.9)** | **2 (1.3 , 3)** |
| **Angola** | **1354 (1094 , 1699)** | **20.6 (18.7 , 22.7)** | **29.8 (24.5 , 36.6)** | **212 (138 , 332)** | **3.4 (2.4 , 4.8)** | **3.4 (2.3 , 5)** |
| **Central African Republic** | **203 (133 , 312)** | **15.1 (12 , 18.8)** | **21.8 (14.9 , 32)** | **26 (14 , 44)** | **2 (1.2 , 3)** | **2.1 (1.2 , 3.5)** |
| **Congo** | **292 (231 , 369)** | **18.3 (16.1 , 21)** | **26.9 (21.8 , 33)** | **29 (18 , 48)** | **1.6 (1.1 , 2.4)** | **2.1 (1.4 , 3.3)** |
| **Democratic Republic of the Congo** | **2426 (1481 , 4053)** | **13.8 (10.6 , 19.4)** | **16.5 (10 , 27.3)** | **312 (182 , 508)** | **1.6 (1.1 , 2.4)** | **1.6 (0.9 , 2.5)** |
| **Equatorial Guinea** | **47 (32 , 67)** | **16.6 (13.5 , 20.2)** | **26.7 (18.8 , 36.9)** | **5 (3 , 8)** | **1.6 (1 , 2.3)** | **1.9 (1.2 , 3.1)** |
| **Gabon** | **134 (100 , 175)** | **16.8 (14.5 , 19.4)** | **29.4 (22.7 , 37.5)** | **13 (8 , 19)** | **2 (1.4 , 2.7)** | **2.3 (1.5 , 3.4)** |
