# Supplementary material for: Global, regional, and national burden of cancers attributable to tobacco smoking in 204 countries and territories, 1990–2019
Source: Cancer Med. 2022 May 27;11(13):2662–78. doi: 10.1002/cam4.4647 (PMC9249976; doi:10.1002/cam4.4647)
Supplement: Supplementary file 13 — Table S2 [file CAM4-11-2662-s013.doc]

| **Table S2: Number, proportion and age-standardised rates of disability-adjusted-life-years (DALYs) due to cancers attributable to smoking (per 100,000) in 2019, by sex and location (Generated from data available from http://ghdx.healthdata.org/gbd-results-tool)** | | | | | | |
| --- | --- | --- | --- | --- | --- | --- |
|  | **Male** | | | **Female** | | |
|  | **No**  **(95% UI)** | **PAF**  **(95% UI)** | **ASRs per 100,000 (95% UI)** | **No**  **(95% UI)** | **PAF**  **(95% UI)** | **ASRs per 100,000 (95% UI)** |
| **Global** | **46695313 (42076550 , 51715585)** | **33 (31.5 , 34.6)** | **1184.6 (1067.6 , 1310.8)** | **9751606 (8835797 , 10652179)** | **8.9 (8.2 , 9.5)** | **222.9 (202.1 , 243.5)** |
| **High-income North America** | **3217723 (3050533 , 3381293)** | **32.2 (30.4 , 33.8)** | **1121.6 (1063.8 , 1177.9)** | **2130096 (1975420 , 2285686)** | **24.8 (23.4 , 26.1)** | **660.3 (615.3 , 706.9)** |
| **Canada** | **306486 (284093 , 329910)** | **29.5 (27.4 , 31.3)** | **959.5 (890.1 , 1030.6)** | **212120 (190435 , 231240)** | **23.5 (21.7 , 25.3)** | **609.4 (551.2 , 663.7)** |
| **Greenland** | **1043 (823 , 1263)** | **43.5 (40.4 , 46.9)** | **2570.8 (2064.5 , 3078.5)** | **616 (496 , 760)** | **37.1 (33.4 , 40.9)** | **1818.5 (1461.3 , 2226.9)** |
| **United States of America** | **2910143 (2760082 , 3059309)** | **32.5 (30.7 , 34.2)** | **1141.2 (1082.8 , 1199.9)** | **1917326 (1777847 , 2061310)** | **24.9 (23.5 , 26.3)** | **666.3 (620.4 , 713.4)** |
| **Australasia** | **149217 (138277 , 161397)** | **20.3 (18.8 , 21.8)** | **666.6 (619.2 , 719.5)** | **95425 (86893 , 103791)** | **16.2 (15 , 17.4)** | **395.5 (361.5 , 430.3)** |
| **Australia** | **124584 (114813 , 135372)** | **20.2 (18.7 , 21.7)** | **663 (611 , 718)** | **76745 (69506 , 83988)** | **15.9 (14.7 , 17.2)** | **379.4 (344.3 , 415.8)** |
| **New Zealand** | **24633 (22650 , 26830)** | **21 (19.4 , 22.6)** | **686.9 (631.8 , 746.8)** | **18680 (16841 , 20419)** | **17.6 (16.1 , 19.1)** | **479.9 (432 , 525.5)** |
| **High-income Asia Pacific** | **2049120 (1872696 , 2195931)** | **34.4 (32.5 , 36.2)** | **1060.8 (975.8 , 1133.5)** | **380428 (328155 , 427881)** | **9.8 (8.9 , 10.6)** | **172.8 (152.5 , 192.1)** |
| **Brunei Darussalam** | **1934 (1608 , 2296)** | **25.2 (23.1 , 27.5)** | **1434.3 (1215 , 1683.8)** | **663 (508 , 857)** | **8 (6.3 , 10)** | **431.2 (344.5 , 540.7)** |
| **Japan** | **1504774 (1366575 , 1610462)** | **33.8 (32 , 35.7)** | **1017.9 (936.1 , 1083.8)** | **312865 (266536 , 354620)** | **10.5 (9.5 , 11.6)** | **195.1 (170.9 , 217.4)** |
| **Singapore** | **23351 (21104 , 25549)** | **26.6 (24.4 , 28.7)** | **605.4 (544.6 , 661.8)** | **5510 (4610 , 6494)** | **7.4 (6.3 , 8.7)** | **135.6 (113.5 , 159.1)** |
| **Republic of Korea** | **519061 (465923 , 577043)** | **36.5 (33.7 , 39.3)** | **1257.3 (1132.3 , 1390.4)** | **61391 (50977 , 73096)** | **7.3 (6.2 , 8.6)** | **125.8 (104.7 , 149.4)** |
| **Western Europe** | **4702249 (4451464 , 4957157)** | **33.3 (31.8 , 35)** | **1218.7 (1156.3 , 1282.2)** | **2083445 (1927890 , 2223076)** | **19.3 (18.2 , 20.4)** | **497.1 (464.3 , 530.5)** |
| **Andorra** | **1093 (828 , 1378)** | **32.8 (30.3 , 35.4)** | **1533.8 (1163.8 , 1934.8)** | **265 (182 , 369)** | **15.8 (13.5 , 18.2)** | **393.5 (269.7 , 549.4)** |
| **Austria** | **81354 (76154 , 86467)** | **33 (30.8 , 34.8)** | **1075 (1009.7 , 1140.8)** | **40648 (37372 , 44189)** | **20.4 (19 , 21.8)** | **489 (449.2 , 530.3)** |
| **Belgium** | **138860 (130511 , 148019)** | **37.1 (34.6 , 39)** | **1412.8 (1329.2 , 1505.9)** | **57701 (52849 , 62774)** | **20.4 (19 , 21.9)** | **555.6 (511.4 , 605.3)** |
| **Cyprus** | **10457 (9227 , 11746)** | **37.9 (35.8 , 40.2)** | **1111.8 (981.6 , 1250.1)** | **2824 (2437 , 3253)** | **13.1 (11.7 , 14.5)** | **283.9 (245.5 , 327.1)** |
| **Denmark** | **63278 (58513 , 68591)** | **32.5 (30.7 , 34.9)** | **1190.9 (1102.6 , 1289.1)** | **46971 (42118 , 51891)** | **27.7 (25.6 , 29.6)** | **823.5 (742 , 901.4)** |
| **Finland** | **39292 (35998 , 42680)** | **26 (24.3 , 27.7)** | **731 (670 , 793.2)** | **18445 (16520 , 20532)** | **14.4 (13.3 , 15.7)** | **313.1 (282.3 , 347)** |
| **France** | **774586 (722051 , 830555)** | **34 (32 , 36)** | **1415.1 (1317.4 , 1517.4)** | **267435 (240283 , 293619)** | **17.3 (16 , 18.7)** | **448.7 (405.3 , 491.4)** |
| **Germany** | **998090 (929575 , 1066170)** | **33.5 (31.7 , 35.6)** | **1244.6 (1164.2 , 1328.7)** | **459617 (420341 , 497292)** | **19.6 (18.4 , 21)** | **533 (488.4 , 576.6)** |
| **Greece** | **178979 (166898 , 190998)** | **45.5 (43.4 , 47.6)** | **1844.8 (1726.3 , 1961.5)** | **53703 (48758 , 58400)** | **20.2 (18.8 , 21.8)** | **520.8 (476.6 , 567)** |
| **Iceland** | **2152 (1929 , 2415)** | **27.2 (25.5 , 29)** | **833.2 (747.1 , 933.2)** | **1271 (1120 , 1432)** | **22.4 (20.5 , 24.2)** | **477.8 (420.5 , 537.6)** |
| **Ireland** | **32284 (29514 , 35094)** | **28.2 (26.3 , 30)** | **910.3 (833.7 , 991.7)** | **22393 (20285 , 24760)** | **22 (20.5 , 23.7)** | **590.1 (535.1 , 652.3)** |
| **Israel** | **45151 (41610 , 48873)** | **28.1 (26.3 , 29.8)** | **870 (802.2 , 941.7)** | **18742 (16754 , 20845)** | **13 (11.9 , 14.3)** | **316.9 (283.8 , 352.9)** |
| **Italy** | **655451 (608076 , 697627)** | **32 (30.4 , 33.8)** | **1097.5 (1025.6 , 1163.6)** | **246499 (222156 , 269242)** | **15.7 (14.6 , 16.9)** | **380 (345.9 , 413)** |
| **Luxembourg** | **5103 (4419 , 5873)** | **33.5 (30.8 , 36.4)** | **1102.4 (955.1 , 1268.2)** | **2193 (1842 , 2577)** | **18.4 (16.3 , 20.4)** | **446.9 (375.5 , 525.7)** |
| **Malta** | **4015 (3583 , 4461)** | **33.7 (31.7 , 35.6)** | **962.5 (862 , 1067.1)** | **1092 (940 , 1281)** | **12.5 (11.3 , 13.7)** | **265.8 (230.2 , 312.1)** |
| **Monaco** | **889 (721 , 1063)** | **36.8 (33.8 , 39.7)** | **2209.2 (1773.3 , 2682.1)** | **416 (310 , 536)** | **21.6 (18.8 , 24.6)** | **980.6 (729.3 , 1286.2)** |
| **Netherlands** | **200881 (186811 , 216191)** | **33.1 (31.2 , 35.2)** | **1269.7 (1180.8 , 1366.1)** | **128402 (117391 , 140442)** | **25 (23.3 , 26.8)** | **774.2 (709.3 , 845.7)** |
| **Norway** | **28424 (26027 , 30711)** | **21.2 (19.6 , 22.8)** | **639.7 (586.2 , 691.1)** | **18561 (16496 , 20625)** | **16 (14.6 , 17.6)** | **408 (365.4 , 451)** |
| **Portugal** | **115525 (107253 , 124016)** | **30.2 (28.4 , 32.1)** | **1249.8 (1162.9 , 1338.8)** | **20264 (17768 , 22987)** | **8.2 (7.3 , 9.3)** | **203.9 (178.3 , 232.4)** |
| **San Marino** | **374 (238 , 538)** | **28.4 (25.8 , 31)** | **1320.8 (829.9 , 1917.9)** | **129 (81 , 204)** | **14.2 (12.2 , 16.3)** | **447.5 (275.7 , 714.8)** |
| **Spain** | **596280 (553739 , 638638)** | **39.5 (37.5 , 41.6)** | **1505.8 (1399.4 , 1607.9)** | **152578 (138469 , 167398)** | **15.9 (14.5 , 17.2)** | **380.3 (346 , 416.9)** |
| **Sweden** | **55243 (50278 , 59612)** | **21.2 (19.5 , 22.9)** | **566.2 (519.1 , 610)** | **50230 (45506 , 54495)** | **21.3 (19.8 , 22.8)** | **499.5 (456.7 , 541.9)** |
| **Switzerland** | **65645 (60633 , 70919)** | **29.8 (27.7 , 31.7)** | **854.2 (789.2 , 922.4)** | **34935 (31312 , 38081)** | **19.9 (18.4 , 21.4)** | **425.3 (382.9 , 463)** |
| **United Kingdom** | **604744 (569436 , 640636)** | **30.4 (28.9 , 32.1)** | **1074 (1013.9 , 1136.2)** | **436316 (402654 , 469658)** | **24.8 (23.4 , 26.3)** | **704.7 (655.9 , 755.1)** |
| **Southern Latin America** | **405324 (376275 , 433508)** | **26.8 (25.1 , 28.3)** | **1085.2 (1007.8 , 1161.1)** | **225921 (202317 , 251505)** | **16.6 (14.8 , 18.3)** | **516.7 (462.1 , 577.3)** |
| **Argentina** | **297436 (274265 , 319774)** | **28.6 (26.6 , 30.3)** | **1240.1 (1143.9 , 1332.8)** | **163261 (145104 , 182859)** | **17.4 (15.5 , 19.4)** | **578 (510.9 , 650.2)** |
| **Chile** | **68820 (62321 , 75784)** | **19.3 (17.7 , 20.8)** | **619.4 (560.7 , 680.8)** | **48307 (42277 , 54436)** | **14.5 (12.8 , 16.1)** | **378.1 (330.4 , 429.1)** |
| **Uruguay** | **39047 (36205 , 41780)** | **33.4 (31.6 , 35.3)** | **1776.2 (1646.2 , 1900.9)** | **14341 (12828 , 16197)** | **15.2 (13.7 , 17)** | **554.4 (493.3 , 630.2)** |
| **Eastern Europe** | **2654410 (2274070 , 3032206)** | **42.4 (40.8 , 44)** | **1926.3 (1651.1 , 2197.2)** | **353261 (294966 , 429030)** | **7.1 (6.3 , 8)** | **192.5 (159.1 , 237.4)** |
| **Belarus** | **129188 (99112 , 165117)** | **43.3 (40.9 , 45.6)** | **2018 (1554.7 , 2578.1)** | **13794 (10090 , 18519)** | **6.8 (5.7 , 8.1)** | **168.5 (121 , 228.1)** |
| **Estonia** | **16640 (12938 , 21104)** | **38.2 (35.9 , 40.8)** | **1702.9 (1323 , 2156.1)** | **5169 (3891 , 6616)** | **14 (12.6 , 15.3)** | **380.4 (286.1 , 491)** |
| **Latvia** | **26458 (20712 , 33753)** | **39.6 (37.3 , 42)** | **1847.3 (1444.4 , 2351.8)** | **5280 (3966 , 6984)** | **9.5 (8.4 , 10.7)** | **263 (192.6 , 354.1)** |
| **Lithuania** | **36469 (29463 , 44893)** | **36.9 (34.8 , 39.5)** | **1735.7 (1402.5 , 2134)** | **6096 (4729 , 7771)** | **8 (7 , 9.2)** | **222.4 (169.7 , 285.7)** |
| **Republic of Moldova** | **41234 (35471 , 47531)** | **41.1 (38.8 , 43.4)** | **1648.6 (1422.4 , 1893.9)** | **3151 (2415 , 4072)** | **4.6 (3.7 , 5.7)** | **98.2 (75 , 127.2)** |
| **Russian Federation** | **1752125 (1429713 , 2116612)** | **43.3 (41.7 , 45)** | **1855.7 (1514.9 , 2235.3)** | **259704 (208913 , 325835)** | **7.6 (6.8 , 8.5)** | **205.3 (162.8 , 261.2)** |
| **Ukraine** | **652295 (521721 , 802453)** | **40.8 (38.7 , 42.6)** | **2193.3 (1758.4 , 2699.4)** | **60066 (44962 , 79367)** | **5.5 (4.6 , 6.4)** | **153.2 (113.7 , 205.1)** |
| **Central Europe** | **1889006 (1626945 , 2174156)** | **41.1 (39.7 , 42.8)** | **2046.7 (1764.1 , 2358.1)** | **652458 (559397 , 760809)** | **20.1 (18.7 , 21.6)** | **610.6 (522.8 , 719.9)** |
| **Albania** | **28407 (20543 , 38013)** | **40.1 (37.2 , 42.7)** | **1376.7 (996 , 1839)** | **3428 (2527 , 4585)** | **8.4 (7.1 , 9.8)** | **153.4 (112.9 , 205.7)** |
| **Bosnia and Herzegovina** | **59960 (46336 , 76226)** | **46.6 (44.3 , 49)** | **2186.2 (1697.2 , 2777.7)** | **18434 (14379 , 23794)** | **20.2 (18.5 , 22)** | **593.8 (460 , 771)** |
| **Bulgaria** | **136420 (107228 , 170407)** | **43.8 (41.7 , 45.9)** | **2311.9 (1817.1 , 2906.9)** | **36750 (28460 , 47478)** | **17 (15.2 , 18.8)** | **595 (454.9 , 783.7)** |
| **Croatia** | **70513 (55179 , 90083)** | **39.6 (37.6 , 41.8)** | **1912.9 (1498.8 , 2445.8)** | **21914 (17469 , 28167)** | **18.7 (17.2 , 20.3)** | **510.6 (400.9 , 659.1)** |
| **Czechia** | **135135 (109688 , 167338)** | **36.2 (34.3 , 38.2)** | **1459 (1180.6 , 1804.4)** | **52405 (42792 , 65051)** | **18.9 (17.4 , 20.4)** | **488 (391 , 607.4)** |
| **Hungary** | **186820 (152453 , 228276)** | **43.1 (41.1 , 45.2)** | **2417.5 (1970 , 2950.1)** | **89922 (73168 , 110836)** | **26.3 (24.6 , 28.1)** | **946 (764.3 , 1176.1)** |
| **Montenegro** | **12403 (10069 , 15236)** | **51 (48.5 , 53.5)** | **2697.9 (2191.5 , 3306.9)** | **4416 (3707 , 5285)** | **27.8 (25.1 , 30.5)** | **863.8 (727 , 1037.6)** |
| **North Macedonia** | **37087 (28611 , 47839)** | **44.8 (42.4 , 47.4)** | **2273.3 (1757.7 , 2904.8)** | **9629 (7373 , 12473)** | **17.8 (16.1 , 19.7)** | **579.3 (443 , 751.9)** |
| **Poland** | **648240 (506692 , 814281)** | **41.2 (39.4 , 42.9)** | **2144.8 (1676.9 , 2695.5)** | **255408 (199950 , 320202)** | **22.7 (21.2 , 24.4)** | **697.1 (544.3 , 882)** |
| **Romania** | **310889 (253654 , 379084)** | **40.4 (38.6 , 42.4)** | **2023.6 (1648.4 , 2475.5)** | **69863 (53610 , 88288)** | **13.9 (12.2 , 15.8)** | **410.1 (311.7 , 526.9)** |
| **Serbia** | **166972 (130861 , 210633)** | **44 (41.9 , 46.6)** | **2330.2 (1818.2 , 2942.5)** | **64372 (50041 , 82839)** | **22.9 (21 , 25)** | **842 (647.2 , 1090.8)** |
| **Slovakia** | **68823 (53188 , 88364)** | **36 (33.7 , 38.4)** | **1684.1 (1296.5 , 2146.7)** | **16397 (12427 , 21400)** | **12.2 (10.7 , 13.7)** | **344.1 (258.6 , 453.4)** |
| **Slovenia** | **27335 (21020 , 35871)** | **35.1 (33 , 37.5)** | **1467.2 (1127.6 , 1924.5)** | **9518 (7210 , 12402)** | **18.6 (17.1 , 20.2)** | **475.3 (357.1 , 623.5)** |
| **Central Asia** | **461251 (410938 , 517353)** | **32.1 (30.4 , 33.8)** | **1311.1 (1173.2 , 1463.8)** | **34520 (28573 , 41784)** | **2.7 (2.3 , 3.3)** | **75.8 (63.2 , 90.9)** |
| **Armenia** | **38109 (31584 , 45093)** | **46.5 (44.5 , 48.9)** | **2067.7 (1716.8 , 2439.6)** | **1592 (1195 , 2093)** | **2.5 (2 , 3.1)** | **66 (50 , 86.5)** |
| **Azerbaijan** | **83900 (63664 , 107733)** | **39.7 (36.1 , 43)** | **1739 (1338.8 , 2219)** | **2000 (1359 , 2870)** | **1.3 (1 , 1.7)** | **33.7 (23.3 , 47.6)** |
| **Georgia** | **53283 (43857 , 62989)** | **42.7 (40.5 , 45.3)** | **2182.8 (1797.4 , 2577.6)** | **3629 (2756 , 4675)** | **4.1 (3.4 , 4.9)** | **118.1 (88.7 , 153.6)** |
| **Kazakhstan** | **118816 (101482 , 137719)** | **37.4 (35.2 , 39.6)** | **1528.6 (1312.7 , 1764.5)** | **11753 (9069 , 14834)** | **4.2 (3.4 , 5)** | **110.2 (85.8 , 138.6)** |
| **Kyrgyzstan** | **21403 (18545 , 24672)** | **32.3 (30.3 , 34.3)** | **993.9 (865 , 1141.2)** | **2504 (1855 , 3345)** | **4.2 (3.3 , 5.5)** | **88.3 (66.7 , 116.1)** |
| **Mongolia** | **28028 (20689 , 37658)** | **27.9 (22.6 , 32.8)** | **2646.8 (2008.7 , 3425.3)** | **3025 (2006 , 4298)** | **4.3 (3.2 , 5.6)** | **241.3 (163.4 , 341.3)** |
| **Tajikistan** | **18881 (14742 , 25209)** | **20.1 (18.1 , 23.2)** | **737.2 (584.3 , 969.2)** | **1505 (1096 , 2055)** | **1.7 (1.3 , 2.3)** | **49.1 (35.6 , 66.4)** |
| **Turkmenistan** | **16254 (12781 , 20584)** | **25.2 (23.3 , 26.9)** | **814.1 (642.8 , 1027.1)** | **1965 (1399 , 2674)** | **3.4 (2.7 , 4.3)** | **78.8 (56.2 , 106.6)** |
| **Uzbekistan** | **82575 (67223 , 98976)** | **21.8 (20.1 , 23.7)** | **759.7 (622.3 , 898.4)** | **6547 (4668 , 9116)** | **1.7 (1.2 , 2.1)** | **44.9 (32.6 , 61.1)** |
| **Central Latin America** | **399117 (326872 , 486033)** | **13.7 (12.5 , 14.8)** | **366.8 (301.2 , 444)** | **167429 (129537 , 217385)** | **5.3 (4.4 , 6.3)** | **130.4 (101.2 , 169.1)** |
| **Colombia** | **72334 (53239 , 96328)** | **12.5 (11 , 13.8)** | **301.8 (222.7 , 400.4)** | **44335 (33464 , 60338)** | **6.8 (5.7 , 8)** | **155.9 (117.6 , 211.9)** |
| **Costa Rica** | **11565 (8754 , 15045)** | **15 (13.6 , 16.4)** | **486.1 (369 , 630.5)** | **3729 (2786 , 5048)** | **5.5 (4.6 , 6.6)** | **134.5 (101.1 , 181.4)** |
| **El Salvador** | **7200 (5077 , 9678)** | **11.3 (9.8 , 12.7)** | **292.5 (206 , 393.4)** | **3781 (2511 , 5680)** | **4.6 (3.4 , 6)** | **113.4 (75.4 , 170.9)** |
| **Guatemala** | **14049 (10187 , 18468)** | **9 (7.6 , 10.6)** | **283.4 (207.2 , 371.6)** | **6149 (3859 , 9299)** | **3.1 (2.2 , 4.2)** | **98.2 (62.9 , 148.1)** |
| **Honduras** | **16937 (12292 , 22049)** | **19.3 (15.6 , 22.6)** | **591.5 (429.4 , 764.4)** | **7300 (4859 , 10471)** | **5.8 (4.1 , 7.9)** | **221.6 (148.8 , 316.8)** |
| **Mexico** | **178863 (141063 , 224281)** | **12.9 (11.6 , 14.2)** | **332.2 (263 , 415.1)** | **59820 (44184 , 79464)** | **4 (3.3 , 5)** | **94.3 (70.6 , 123.6)** |
| **Nicaragua** | **7040 (5508 , 8665)** | **12.4 (10.9 , 13.9)** | **365.4 (286.9 , 448.4)** | **2092 (1370 , 3050)** | **3.3 (2.3 , 4.6)** | **78 (51.7 , 112.3)** |
| **Panama** | **6308 (4680 , 8199)** | **12.5 (11.3 , 13.8)** | **314.4 (233.8 , 408.8)** | **1970 (1392 , 2869)** | **4 (3.2 , 5.2)** | **92.9 (65.5 , 135.2)** |
| **Venezuela (Bolivarian Republic of)** | **84821 (61972 , 112790)** | **18.5 (16.7 , 20.8)** | **605.3 (446.6 , 801.4)** | **38252 (25873 , 55229)** | **8.4 (6.5 , 10.6)** | **242.9 (165.1 , 349)** |
| **Andean Latin America** | **67222 (51698 , 83585)** | **8.9 (7.7 , 10.1)** | **252.5 (195.1 , 313.2)** | **23625 (16778 , 32900)** | **2.7 (2 , 3.5)** | **80.1 (57.5 , 110.9)** |
| **Bolivia (Plurinational State of)** | **18723 (12866 , 25349)** | **11.6 (9.3 , 13.6)** | **447.3 (308.6 , 603.2)** | **6016 (3619 , 9476)** | **2.9 (2 , 4.2)** | **124.9 (76.3 , 195.3)** |
| **Ecuador** | **23420 (17954 , 30487)** | **11.1 (9.8 , 12.6)** | **327.4 (251.3 , 424.4)** | **8936 (6418 , 12154)** | **3.8 (3 , 4.9)** | **112.4 (81 , 152.4)** |
| **Peru** | **25079 (17254 , 35227)** | **6.5 (5.4 , 7.5)** | **164.6 (113.5 , 230.8)** | **8673 (5602 , 13095)** | **1.9 (1.4 , 2.6)** | **51.7 (33.5 , 77.8)** |
| **Caribbean** | **222545 (187494 , 261711)** | **24.9 (22.8 , 26.9)** | **904.9 (762.8 , 1062.4)** | **87312 (70877 , 106437)** | **10.6 (9 , 12.2)** | **319.9 (259.6 , 391.3)** |
| **Antigua and Barbuda** | **216 (177 , 262)** | **13.3 (11.8 , 15.1)** | **440.9 (361.3 , 534.2)** | **87 (65 , 116)** | **5.3 (4.2 , 6.9)** | **155.8 (116.8 , 208.3)** |
| **Barbados** | **1086 (861 , 1350)** | **12.1 (10.7 , 13.6)** | **489.7 (389.3 , 607)** | **235 (171 , 323)** | **2.6 (2.1 , 3.4)** | **94.6 (68.3 , 131.8)** |
| **Belize** | **816 (687 , 955)** | **18 (16.4 , 19.8)** | **567.1 (478.9 , 660.4)** | **189 (133 , 269)** | **4.6 (3.4 , 6.4)** | **126.9 (93.1 , 174.9)** |
| **Bermuda** | **499 (412 , 604)** | **22.5 (20.2 , 24.7)** | **863.2 (713.4 , 1044.9)** | **133 (103 , 172)** | **9.6 (8.2 , 11.3)** | **192.1 (148.6 , 248.2)** |
| **Bahamas** | **1209 (956 , 1509)** | **15 (13.2 , 16.7)** | **641.6 (509.1 , 801)** | **281 (200 , 381)** | **3.7 (2.8 , 4.8)** | **125.6 (89.7 , 169.2)** |
| **Cuba** | **133020 (107355 , 164683)** | **38.1 (36 , 41.5)** | **1490.1 (1198.4 , 1847.4)** | **50961 (39673 , 63349)** | **19.1 (17 , 21.7)** | **525.8 (410 , 656.5)** |
| **Dominica** | **316 (250 , 394)** | **13.2 (11.7 , 14.9)** | **702.2 (556.1 , 875)** | **68 (50 , 93)** | **4.3 (3.4 , 5.4)** | **156.6 (112.5 , 214.8)** |
| **Dominican Republic** | **29494 (21069 , 39857)** | **19.4 (17.4 , 21.1)** | **660.7 (476.9 , 888.8)** | **15908 (11107 , 22002)** | **12.1 (9.8 , 14.7)** | **328.6 (229.7 , 451.7)** |
| **Grenada** | **382 (330 , 436)** | **14.8 (13.1 , 16.7)** | **666.2 (578.4 , 755.9)** | **95 (71 , 128)** | **4.3 (3.3 , 5.6)** | **160.1 (120.5 , 216.5)** |
| **Guyana** | **1376 (1026 , 1830)** | **13.5 (11.9 , 15)** | **429.7 (322.8 , 565.2)** | **494 (317 , 742)** | **3.9 (2.8 , 5.4)** | **138.2 (90.7 , 203.7)** |
| **Haiti** | **12185 (7550 , 17941)** | **8.4 (7 , 10.3)** | **354.1 (222.2 , 520.2)** | **5046 (2674 , 8304)** | **2.5 (1.7 , 3.7)** | **116.5 (64.5 , 185.5)** |
| **Jamaica** | **11739 (9112 , 15044)** | **21.4 (19.3 , 24.6)** | **813.8 (632.8 , 1041.5)** | **3104 (2229 , 4274)** | **6.1 (4.7 , 7.8)** | **205.3 (147.7 , 283.1)** |
| **Puerto Rico** | **13847 (10270 , 18058)** | **18.3 (16.2 , 20.4)** | **462.1 (341.6 , 606.5)** | **5228 (3903 , 6909)** | **8 (6.8 , 9.4)** | **145.8 (108.4 , 194.3)** |
| **Saint Kitts and Nevis** | **177 (140 , 214)** | **12.1 (10.7 , 13.6)** | **502.2 (406 , 598.8)** | **39 (27 , 56)** | **3.6 (2.6 , 4.8)** | **110.3 (77.1 , 152.8)** |
| **Saint Lucia** | **696 (571 , 843)** | **16.7 (15.1 , 18.3)** | **660.2 (543.8 , 798.5)** | **158 (116 , 214)** | **5 (3.9 , 6.3)** | **138.6 (102.1 , 188.5)** |
| **Saint Vincent and the Grenadines** | **374 (315 , 452)** | **13.6 (12 , 15.5)** | **519.8 (439.2 , 624.8)** | **84 (60 , 118)** | **3.6 (2.6 , 4.9)** | **126.8 (90.9 , 177.6)** |
| **Suriname** | **2155 (1735 , 2663)** | **22.7 (20.5 , 25.2)** | **740.7 (598.7 , 915.6)** | **780 (576 , 1039)** | **8.1 (6.4 , 10.3)** | **235.7 (174.1 , 312.9)** |
| **Trinidad and Tobago** | **4718 (3394 , 6361)** | **18.5 (16.6 , 20.6)** | **508.4 (367.4 , 682.1)** | **1222 (823 , 1788)** | **5.1 (4 , 6.6)** | **127.9 (86.8 , 187.9)** |
| **United States Virgin Islands** | **702 (566 , 843)** | **15.3 (13.3 , 17.5)** | **818.3 (655.4 , 989.4)** | **241 (185 , 313)** | **8.5 (6.9 , 10.2)** | **234.2 (177.9 , 307)** |
| **Tropical Latin America** | **822064 (758184 , 882645)** | **22.4 (21.1 , 23.7)** | **731.2 (674.3 , 785.1)** | **433000 (382623 , 489706)** | **13 (11.7 , 14.6)** | **322.2 (284.6 , 364.6)** |
| **Brazil** | **799589 (737526 , 858379)** | **22.3 (20.9 , 23.6)** | **728.5 (672 , 781.9)** | **425795 (377071 , 482013)** | **13.1 (11.7 , 14.6)** | **323.8 (286.9 , 366.6)** |
| **Paraguay** | **22475 (16774 , 29525)** | **26.3 (24 , 28.6)** | **842.4 (635.8 , 1099.9)** | **7205 (4960 , 10501)** | **9.2 (7 , 11.9)** | **246.6 (172.1 , 356.7)** |
| **East Asia** | **19202193 (15279576 , 23731755)** | **42.6 (40.8 , 44.2)** | **1851.3 (1480.6 , 2268.7)** | **1799259 (1434790 , 2259128)** | **7.2 (6.4 , 8.1)** | **164.4 (131 , 205.8)** |
| **China** | **18650350 (14730476 , 23199537)** | **42.8 (41 , 44.5)** | **1859.4 (1480.9 , 2286.4)** | **1750559 (1386024 , 2215270)** | **7.3 (6.5 , 8.2)** | **166.3 (131.2 , 209.4)** |
| **Democratic People's Republic of Korea** | **248069 (192795 , 309267)** | **38 (34.7 , 41.4)** | **1718.1 (1359.1 , 2115.4)** | **26771 (18640 , 36407)** | **5.4 (4.1 , 6.9)** | **144.4 (101 , 198.8)** |
| **Taiwan (Province of China)** | **303774 (234537 , 399059)** | **36.5 (34.5 , 38.5)** | **1626.2 (1257.8 , 2134.8)** | **21929 (15681 , 30026)** | **4.4 (3.5 , 5.4)** | **107.8 (77.6 , 147.9)** |
| **Southeast Asia** | **3122754 (2683134 , 3613470)** | **31.9 (29.5 , 34.2)** | **1061.2 (916.2 , 1223.1)** | **346832 (271554 , 440044)** | **3.8 (3.2 , 4.4)** | **106.7 (84.2 , 133.9)** |
| **Cambodia** | **74227 (57406 , 88916)** | **34.4 (31.6 , 37.4)** | **1488.6 (1161.5 , 1758.3)** | **9852 (7119 , 13223)** | **4.5 (3.6 , 5.7)** | **142.7 (104.9 , 188.1)** |
| **Indonesia** | **1085843 (840168 , 1392688)** | **33.3 (29.8 , 36.4)** | **1017.3 (798.1 , 1281.1)** | **100685 (61794 , 148236)** | **2.8 (2.1 , 3.7)** | **89.9 (55.4 , 131.6)** |
| **Lao People's Democratic Republic** | **23744 (17398 , 30271)** | **31.3 (28.5 , 34.2)** | **1105.4 (830.9 , 1388.9)** | **3201 (2178 , 4643)** | **4 (3.1 , 5.1)** | **137.6 (95.2 , 196.8)** |
| **Malaysia** | **128608 (99438 , 162657)** | **29 (26.6 , 31.4)** | **933.7 (730.2 , 1178.2)** | **8980 (6476 , 12256)** | **2.2 (1.7 , 2.8)** | **69.4 (50.4 , 93.3)** |
| **Maldives** | **816 (662 , 987)** | **24.1 (21.9 , 26.1)** | **497.7 (402.1 , 598.3)** | **93 (69 , 123)** | **3.6 (2.8 , 4.6)** | **71.5 (52.8 , 93.9)** |
| **Mauritius** | **5020 (4016 , 6242)** | **26.6 (24.6 , 28.3)** | **605.9 (486.1 , 748.8)** | **510 (372 , 684)** | **2.5 (2 , 3.1)** | **53.3 (38.7 , 71)** |
| **Myanmar** | **191476 (144127 , 258026)** | **26 (22.4 , 30)** | **931.3 (707.2 , 1252.1)** | **57413 (44106 , 79740)** | **7.2 (5.9 , 8.7)** | **223.2 (173.5 , 309.9)** |
| **Philippines** | **353666 (264616 , 465246)** | **27.9 (25.7 , 29.8)** | **918.6 (694.9 , 1197.2)** | **72068 (54210 , 95702)** | **5.6 (4.8 , 6.4)** | **174.1 (132.7 , 229.1)** |
| **Sri Lanka** | **56922 (40910 , 77857)** | **23.1 (21 , 25.5)** | **478.7 (346 , 652)** | **4482 (3102 , 6256)** | **1.9 (1.5 , 2.4)** | **31.2 (21.9 , 43.4)** |
| **Seychelles** | **839 (719 , 989)** | **29.4 (27.4 , 31.5)** | **1527.5 (1311.6 , 1786.7)** | **75 (55 , 106)** | **3.8 (2.9 , 5)** | **129 (94.6 , 179.6)** |
| **Thailand** | **509332 (359586 , 689190)** | **30.4 (26.7 , 33.9)** | **1058.1 (749.9 , 1419.5)** | **53257 (38268 , 71486)** | **4.3 (3.6 , 5.1)** | **95.4 (68.8 , 127.6)** |
| **Timor-Leste** | **3572 (2329 , 4627)** | **29.6 (25.5 , 34.9)** | **859.3 (569.2 , 1101.3)** | **311 (198 , 466)** | **2.6 (1.8 , 4)** | **75.2 (48.7 , 111.9)** |
| **Viet Nam** | **684597 (531928 , 853854)** | **37.6 (34.8 , 40.8)** | **1540 (1210.9 , 1886.5)** | **35449 (24581 , 49581)** | **2.6 (2 , 3.3)** | **68 (47.4 , 94.3)** |
| **Oceania** | **28216 (20797 , 38267)** | **21.1 (18.3 , 24.2)** | **730.3 (553.5 , 982.6)** | **11237 (7687 , 15664)** | **6.7 (5 , 8.6)** | **282.3 (200.9 , 382.5)** |
| **American Samoa** | **238 (200 , 276)** | **25.9 (23.3 , 28.6)** | **997.6 (844.6 , 1148.5)** | **96 (74 , 126)** | **10.5 (8.7 , 12.8)** | **364.6 (279.9 , 476.3)** |
| **Cook Islands** | **126 (105 , 151)** | **28.3 (25.3 , 31.5)** | **1019 (837.7 , 1222.8)** | **27 (19 , 35)** | **9.5 (7.7 , 11.5)** | **212.5 (151.9 , 281.5)** |
| **Micronesia (Federated States of)** | **501 (303 , 744)** | **28.9 (24 , 34.5)** | **1367.3 (873.8 , 1987.9)** | **244 (151 , 359)** | **13.5 (10 , 17.4)** | **586.3 (372.4 , 847)** |
| **Fiji** | **1934 (1464 , 2501)** | **18.9 (16.5 , 21.1)** | **527.5 (409.4 , 668.7)** | **881 (533 , 1352)** | **6 (4 , 8.2)** | **203.2 (125.3 , 307.9)** |
| **Guam** | **896 (711 , 1088)** | **27.6 (24.7 , 30.6)** | **931.7 (744.7 , 1129.4)** | **303 (237 , 387)** | **14 (11.8 , 16.5)** | **312.7 (243.9 , 398.1)** |
| **Kiribati** | **566 (419 , 740)** | **28.7 (25.9 , 31.3)** | **1691.6 (1275.1 , 2160.9)** | **521 (335 , 763)** | **20.7 (14.4 , 27.2)** | **1163.8 (770.3 , 1672.2)** |
| **Marshall Islands** | **201 (132 , 298)** | **23.5 (18.7 , 28.7)** | **1037.8 (695.8 , 1532.8)** | **56 (34 , 85)** | **5.9 (4.2 , 8)** | **275.6 (173.9 , 409.8)** |
| **Nauru** | **30 (19 , 42)** | **21.2 (17.6 , 24.7)** | **1425.2 (971.6 , 1910.5)** | **23 (14 , 34)** | **15 (11.5 , 18.5)** | **742.9 (475.2 , 1080.7)** |
| **Niue** | **10 (8 , 12)** | **26.7 (23.7 , 30.6)** | **984.5 (803.8 , 1178.9)** | **4 (3 , 5)** | **10.6 (8.6 , 13)** | **320.6 (220.5 , 447.2)** |
| **Northern Mariana Islands** | **442 (368 , 513)** | **33 (29.8 , 36.5)** | **1527.2 (1303.3 , 1734.1)** | **103 (74 , 141)** | **11.2 (8.6 , 14.2)** | **355.4 (256 , 479.9)** |
| **Palau** | **131 (102 , 171)** | **25.2 (22.1 , 28.5)** | **1095 (871.4 , 1412.4)** | **59 (40 , 84)** | **11.4 (8.7 , 14.9)** | **504 (345.4 , 719.7)** |
| **Papua New Guinea** | **17458 (11853 , 25658)** | **19.7 (16.3 , 23.5)** | **653.1 (454.6 , 946.8)** | **6977 (4319 , 10356)** | **6.2 (4.4 , 8.3)** | **263.6 (172.2 , 381.1)** |
| **Samoa** | **518 (416 , 654)** | **24.9 (21.9 , 27.7)** | **696.3 (563.9 , 864)** | **243 (172 , 340)** | **9.6 (7.8 , 11.9)** | **313.5 (224.3 , 435.8)** |
| **Solomon Islands** | **2490 (1555 , 3836)** | **25.5 (21.1 , 30.5)** | **1456.9 (952.8 , 2208.1)** | **971 (579 , 1485)** | **6.6 (4.7 , 9.1)** | **508.5 (327.5 , 749.5)** |
| **Tokelau** | **5 (4 , 7)** | **28.4 (24.7 , 31.8)** | **730.2 (539.8 , 992.9)** | **3 (2 , 4)** | **10.1 (7.6 , 13)** | **365.8 (235 , 540.7)** |
| **Tonga** | **507 (398 , 630)** | **26.8 (23.3 , 29.9)** | **1381.1 (1091.8 , 1703.4)** | **103 (72 , 142)** | **7.2 (5.5 , 9.3)** | **244 (172.7 , 336.6)** |
| **Tuvalu** | **51 (38 , 69)** | **27.5 (24.1 , 31.4)** | **1017.2 (764.9 , 1366.1)** | **22 (15 , 32)** | **11 (8.7 , 13.8)** | **394.3 (269.3 , 563.1)** |
| **Vanuatu** | **780 (540 , 1102)** | **20.6 (17.6 , 24.4)** | **839.7 (586.5 , 1183)** | **71 (46 , 110)** | **2.2 (1.6 , 3.1)** | **83.4 (54.7 , 125.7)** |
| **North Africa and Middle East** | **2125564 (1869073 , 2427209)** | **31 (29.1 , 33.1)** | **920.4 (810.5 , 1049.6)** | **210904 (179540 , 249200)** | **3.9 (3.4 , 4.4)** | **94.5 (80.9 , 111.4)** |
| **Afghanistan** | **42759 (28955 , 61830)** | **13.4 (10.8 , 16)** | **659.5 (460 , 930.1)** | **6247 (3743 , 9440)** | **1.3 (0.9 , 1.8)** | **76.9 (49.7 , 113.9)** |
| **Algeria** | **94983 (73522 , 119659)** | **27.9 (25.8 , 30.1)** | **544.8 (423.8 , 686.7)** | **5362 (3847 , 7423)** | **1.6 (1.2 , 2.1)** | **32.2 (23.6 , 43.6)** |
| **Bahrain** | **3440 (2570 , 4460)** | **24.3 (21.4 , 27.4)** | **683.2 (528.9 , 861.7)** | **409 (293 , 563)** | **3.9 (3 , 4.9)** | **119.2 (88.1 , 161.4)** |
| **Egypt** | **323842 (231028 , 445643)** | **28.9 (25.3 , 32.5)** | **848.9 (608.5 , 1161.3)** | **5912 (3565 , 8986)** | **0.8 (0.6 , 1.1)** | **20.7 (12.9 , 30.6)** |
| **Iran (Islamic Republic of)** | **236771 (215963 , 260346)** | **23.7 (21.8 , 26.2)** | **638.2 (584.2 , 702.6)** | **28366 (23275 , 33578)** | **3.5 (2.9 , 4.2)** | **75 (61.9 , 88.5)** |
| **Iraq** | **119566 (89582 , 146562)** | **30.9 (28.6 , 33)** | **1046.2 (794.6 , 1259.2)** | **15237 (11163 , 20207)** | **3.9 (3.2 , 4.6)** | **128.8 (95.4 , 169.2)** |
| **Jordan** | **29085 (22297 , 36576)** | **31.8 (29.7 , 33.8)** | **802 (620 , 995.5)** | **4261 (3302 , 5463)** | **5.3 (4.5 , 6.1)** | **131.5 (102.6 , 168.6)** |
| **Kuwait** | **6717 (5170 , 8362)** | **24.4 (22.6 , 26.3)** | **445.9 (344.8 , 552.1)** | **481 (353 , 633)** | **2.4 (1.9 , 3)** | **45.4 (32.8 , 60)** |
| **Lebanon** | **34278 (27549 , 44282)** | **37.5 (34.4 , 41)** | **1462 (1174.1 , 1889.2)** | **15347 (12018 , 20019)** | **16.5 (14.5 , 18.3)** | **541.4 (422.4 , 704.3)** |
| **Libya** | **30215 (22807 , 38472)** | **35 (31.8 , 37.3)** | **1106.2 (833.4 , 1401.9)** | **469 (311 , 686)** | **0.6 (0.5 , 0.8)** | **17.9 (12 , 26.5)** |
| **Morocco** | **133248 (96242 , 171146)** | **34.7 (32.2 , 37.2)** | **790.4 (572.6 , 999.1)** | **4219 (2828 , 6156)** | **1 (0.7 , 1.3)** | **24.4 (16.6 , 34.7)** |
| **Palestine** | **13907 (11722 , 16398)** | **30.4 (27.9 , 32.7)** | **1184.3 (1005.3 , 1379)** | **879 (638 , 1138)** | **2.1 (1.6 , 2.6)** | **72.5 (52.9 , 93.8)** |
| **Oman** | **3173 (2459 , 4198)** | **12.2 (10.8 , 13.7)** | **327.6 (267 , 407.8)** | **241 (171 , 319)** | **1.3 (1 , 1.6)** | **35 (25.1 , 45.8)** |
| **Qatar** | **3740 (2658 , 5027)** | **19.9 (17.4 , 22.5)** | **597.4 (443.5 , 779.7)** | **109 (72 , 158)** | **1.1 (0.8 , 1.5)** | **45.9 (31.7 , 66.3)** |
| **Saudi Arabia** | **47397 (35416 , 60791)** | **19.1 (16.9 , 21.3)** | **369.5 (288.5 , 455.3)** | **2959 (2014 , 4252)** | **1.4 (1 , 1.8)** | **38 (26.7 , 53.7)** |
| **Sudan** | **58926 (38106 , 83661)** | **19.1 (16.1 , 22.8)** | **571.1 (370.9 , 800.3)** | **4139 (2666 , 6147)** | **1.6 (1.2 , 2.2)** | **45 (29.2 , 64.1)** |
| **Syrian Arab Republic** | **43906 (32088 , 59807)** | **31.4 (27.8 , 34.7)** | **648.3 (478 , 867.5)** | **5015 (3277 , 7263)** | **4.2 (3.1 , 5.5)** | **77.7 (51.6 , 111.1)** |
| **Tunisia** | **71943 (51366 , 99539)** | **47.9 (45.3 , 50.5)** | **1133.4 (814.2 , 1569.8)** | **2476 (1703 , 3557)** | **2.2 (1.7 , 2.8)** | **37.5 (25.8 , 54)** |
| **Turkey** | **744298 (585780 , 930988)** | **44.2 (42 , 46.7)** | **1713.7 (1348 , 2141.3)** | **95689 (74015 , 121190)** | **9.8 (8.6 , 11)** | **200.8 (155.2 , 253.5)** |
| **United Arab Emirates** | **26925 (18485 , 37850)** | **17.5 (14.9 , 19.9)** | **746.4 (572.9 , 953.8)** | **1733 (1186 , 2448)** | **3.1 (2.3 , 4)** | **138.8 (96.9 , 193)** |
| **Yemen** | **54285 (39598 , 74385)** | **25.1 (21.8 , 28.9)** | **795 (584.4 , 1080.2)** | **11141 (7930 , 15571)** | **5.5 (4.5 , 6.7)** | **149.6 (108.1 , 204.4)** |
| **South Asia** | **4260258 (3570703 , 5031652)** | **23.1 (21.3 , 24.6)** | **590.2 (498.3 , 695.7)** | **498768 (386991 , 641288)** | **2.7 (2.2 , 3.2)** | **69.1 (54 , 88.7)** |
| **Bangladesh** | **401544 (287837 , 554645)** | **23.9 (20.1 , 28.3)** | **581.9 (418.3 , 801.6)** | **21091 (14103 , 31045)** | **1.5 (1.1 , 2)** | **33.8 (22.8 , 49)** |
| **Bhutan** | **1093 (780 , 1427)** | **15.8 (13.1 , 18.8)** | **377.9 (272.4 , 490.8)** | **201 (134 , 297)** | **3.2 (2.3 , 4.3)** | **71 (47.8 , 103.3)** |
| **India** | **3084784 (2471627 , 3794069)** | **23 (21.2 , 24.7)** | **532.6 (428.7 , 653.9)** | **378126 (278988 , 509096)** | **2.7 (2.3 , 3.3)** | **64.2 (47.7 , 86.2)** |
| **Nepal** | **54462 (41932 , 66920)** | **17.1 (14.9 , 19.5)** | **507.9 (392.5 , 620.5)** | **25464 (18447 , 33646)** | **8.2 (6.5 , 10)** | **217.4 (157.4 , 285.2)** |
| **Pakistan** | **718376 (538844 , 960718)** | **23.5 (21 , 26)** | **1172.4 (894 , 1541.5)** | **73886 (53390 , 100880)** | **2.3 (1.8 , 2.9)** | **129.2 (94.9 , 176.3)** |
| **Southern Sub-Saharan Africa** | **244791 (217765 , 275162)** | **23.7 (21.9 , 25.5)** | **968.6 (865.4 , 1082.1)** | **76383 (62772 , 94065)** | **7.5 (6.2 , 9)** | **230 (190.7 , 282.2)** |
| **Botswana** | **7575 (5478 , 9791)** | **24.8 (22.3 , 27.2)** | **1196 (897.3 , 1495.7)** | **1990 (1196 , 3122)** | **6.2 (4.6 , 8.1)** | **246.7 (155.5 , 380)** |
| **Lesotho** | **9107 (6972 , 11913)** | **26.2 (23.4 , 29.6)** | **1595.7 (1239.9 , 2052.3)** | **1210 (597 , 2276)** | **3.8 (2.4 , 6.1)** | **167.6 (85.5 , 308.9)** |
| **Namibia** | **2538 (1992 , 3234)** | **11.6 (9.9 , 13.4)** | **425.3 (338.1 , 533.6)** | **1545 (998 , 2262)** | **6.3 (4.7 , 8.2)** | **194.2 (127.7 , 279.3)** |
| **South Africa** | **188532 (164450 , 217252)** | **24.4 (22.4 , 26.4)** | **937.5 (825.7 , 1072.9)** | **61991 (50445 , 75471)** | **8.9 (7.4 , 10.7)** | **234.5 (192.1 , 284.1)** |
| **Eswatini** | **1686 (1166 , 2335)** | **9.7 (7.7 , 11.8)** | **682.5 (480 , 922)** | **419 (231 , 699)** | **3.3 (2.3 , 4.9)** | **128.7 (72.2 , 210.7)** |
| **Zimbabwe** | **35353 (27954 , 42528)** | **22.7 (20.1 , 25.6)** | **1142.5 (925.2 , 1343.9)** | **9229 (5992 , 13441)** | **4.2 (3.1 , 5.6)** | **235 (153.6 , 337.5)** |
| **Western Sub-Saharan Africa** | **255625 (209692 , 311415)** | **8.6 (7.6 , 9.8)** | **278.9 (229.4 , 336.5)** | **38771 (25825 , 57026)** | **1.2 (0.9 , 1.7)** | **36 (25.3 , 50.9)** |
| **Benin** | **7860 (5839 , 10501)** | **9.1 (7.7 , 10.5)** | **352.6 (266.1 , 461.7)** | **1253 (761 , 1944)** | **1.5 (1 , 2.2)** | **45 (28.2 , 68.1)** |
| **Burkina Faso** | **12488 (9274 , 16266)** | **7.8 (6.2 , 9.4)** | **284.8 (215.1 , 369.4)** | **1061 (563 , 1757)** | **0.6 (0.3 , 0.9)** | **18.4 (10.5 , 29.1)** |
| **Cameroon** | **28417 (20486 , 38894)** | **12.7 (10.8 , 14.8)** | **467.1 (339.6 , 633)** | **1969 (1088 , 3270)** | **0.9 (0.6 , 1.3)** | **30.7 (18.2 , 49)** |
| **Cabo Verde** | **1241 (1007 , 1484)** | **11.9 (10.2 , 13.6)** | **662.8 (532.3 , 787)** | **181 (131 , 245)** | **2.7 (2.1 , 3.5)** | **77.8 (56.4 , 105)** |
| **Chad** | **11467 (8311 , 15536)** | **10.3 (8.5 , 12.4)** | **374.4 (274.3 , 505.1)** | **1471 (797 , 2459)** | **1.5 (0.9 , 2.3)** | **52.9 (30.1 , 85)** |
| **Côte d’Ivoire** | **30532 (22429 , 39961)** | **14.7 (13 , 16.5)** | **505.7 (376.7 , 652.6)** | **4787 (2992 , 7392)** | **2.9 (1.9 , 4.2)** | **91.2 (58.4 , 141.2)** |
| **Gambia** | **2483 (1587 , 3593)** | **12.7 (8.6 , 16.8)** | **502 (334.5 , 705.5)** | **129 (75 , 208)** | **0.9 (0.5 , 1.3)** | **25.6 (15.3 , 40.3)** |
| **Ghana** | **17752 (13782 , 22359)** | **6.8 (5.8 , 8)** | **251.1 (198.3 , 311.2)** | **4158 (2602 , 6433)** | **1.3 (0.9 , 1.9)** | **43.4 (27.7 , 66.1)** |
| **Guinea** | **20358 (14092 , 27562)** | **14.5 (11.4 , 17.9)** | **705.6 (497.2 , 941.4)** | **2136 (1226 , 3538)** | **1.7 (1 , 2.8)** | **72 (42.2 , 118.1)** |
| **Guinea-Bissau** | **1249 (877 , 1705)** | **8.2 (6.9 , 9.8)** | **358.1 (256.4 , 483.2)** | **174 (96 , 299)** | **1 (0.6 , 1.6)** | **37.9 (21.6 , 63.8)** |
| **Liberia** | **3109 (2109 , 4320)** | **10.2 (8.7 , 11.8)** | **293.2 (203.6 , 399.7)** | **546 (307 , 907)** | **1.6 (1 , 2.5)** | **49.2 (29.4 , 78.9)** |
| **Mali** | **16755 (11966 , 22753)** | **10.3 (8.2 , 12.5)** | **371.6 (265.4 , 495.6)** | **2151 (1182 , 3420)** | **1.5 (0.9 , 2.4)** | **46.5 (27.1 , 72.6)** |
| **Mauritania** | **3782 (2562 , 5262)** | **14.9 (12.8 , 17.3)** | **354.5 (241.7 , 484.9)** | **860 (507 , 1356)** | **2.8 (1.9 , 3.9)** | **71.9 (43.8 , 111.2)** |
| **Niger** | **7165 (4739 , 10133)** | **6.1 (4.9 , 7.5)** | **188.6 (126.4 , 261.8)** | **991 (490 , 1724)** | **0.8 (0.4 , 1.3)** | **20.6 (10.9 , 34.6)** |
| **Nigeria** | **57433 (40112 , 81850)** | **5 (3.9 , 6.2)** | **143.8 (102.3 , 203.8)** | **11857 (6897 , 19296)** | **0.9 (0.6 , 1.3)** | **23.3 (14.2 , 36.9)** |
| **Sao Tome and Principe** | **220 (170 , 275)** | **10.1 (8.5 , 12)** | **438 (344.9 , 536.5)** | **38 (21 , 63)** | **1.7 (1 , 2.6)** | **67.3 (38.6 , 107.2)** |
| **Senegal** | **16286 (12004 , 21386)** | **13.9 (12.1 , 15.9)** | **437.1 (327.3 , 570.1)** | **1114 (648 , 1842)** | **0.9 (0.6 , 1.4)** | **26.3 (15.5 , 42.9)** |
| **Sierra Leone** | **8179 (5801 , 11116)** | **13.5 (11.4 , 15.8)** | **447.6 (320.2 , 604.4)** | **1889 (1033 , 3272)** | **3 (1.7 , 4.7)** | **90.9 (52.7 , 150.3)** |
| **Togo** | **8847 (6463 , 11820)** | **15.5 (13.3 , 17.6)** | **550.3 (411.1 , 723.9)** | **2005 (1275 , 3144)** | **3.1 (2.1 , 4.5)** | **92.7 (60.3 , 141.5)** |
| **Eastern Sub-Saharan Africa** | **294468 (237413 , 367778)** | **9.3 (8.1 , 10.6)** | **364.7 (298.9 , 451)** | **84534 (54023 , 125545)** | **2.4 (1.6 , 3.4)** | **93.1 (64 , 133.1)** |
| **Burundi** | **9321 (6510 , 13014)** | **10 (8.3 , 11.7)** | **364 (259 , 500.4)** | **2102 (1075 , 3624)** | **2.2 (1.3 , 3.5)** | **77.8 (42.3 , 132.8)** |
| **Comoros** | **979 (687 , 1315)** | **14.6 (12.5 , 16.8)** | **436.1 (314.3 , 577)** | **181 (107 , 299)** | **2 (1.3 , 3)** | **66.6 (39.7 , 108.3)** |
| **Djibouti** | **2501 (1622 , 3853)** | **18.7 (15.4 , 22.6)** | **715.2 (488.6 , 1069.4)** | **331 (183 , 591)** | **2.7 (1.7 , 4.1)** | **111 (66.3 , 183.4)** |
| **Eritrea** | **5440 (3776 , 7368)** | **9.7 (7.8 , 11.7)** | **389.8 (274.8 , 509.1)** | **297 (152 , 542)** | **0.4 (0.2 , 0.7)** | **17 (9.2 , 29.9)** |
| **Ethiopia** | **26403 (18313 , 36750)** | **3.9 (3 , 5.1)** | **126.9 (87.9 , 176.2)** | **3289 (1593 , 5908)** | **0.4 (0.2 , 0.7)** | **14 (7.2 , 24.1)** |
| **Kenya** | **43015 (33289 , 53572)** | **12.3 (10.8 , 13.8)** | **386.6 (303.9 , 471.6)** | **6842 (4510 , 10319)** | **1.7 (1.3 , 2.3)** | **58.7 (39.1 , 87.5)** |
| **Madagascar** | **15149 (10622 , 21132)** | **9.3 (7.6 , 11)** | **258.3 (185.4 , 349.7)** | **3266 (1702 , 5789)** | **1.4 (0.8 , 2.3)** | **47.8 (27.1 , 81.7)** |
| **Malawi** | **20407 (14636 , 27386)** | **10.8 (8.5 , 13.5)** | **620.3 (463.1 , 805.7)** | **4043 (2413 , 6494)** | **2.2 (1.4 , 3.3)** | **96 (59 , 151.2)** |
| **Mozambique** | **21479 (16360 , 27776)** | **9.5 (7.5 , 11.4)** | **436.8 (340.9 , 554.4)** | **6389 (3100 , 11786)** | **2.4 (1.3 , 3.8)** | **94.5 (50.6 , 166.6)** |
| **Rwanda** | **15103 (10841 , 20764)** | **14.3 (12 , 17)** | **589.1 (434.2 , 796)** | **9449 (6439 , 13703)** | **7.2 (5.3 , 9.7)** | **272.4 (194.1 , 380.4)** |
| **Somalia** | **13023 (8137 , 18619)** | **9.1 (6.8 , 11.3)** | **424.8 (267 , 596.3)** | **3213 (1434 , 6024)** | **1.9 (1 , 3.2)** | **76.3 (35.7 , 139.8)** |
| **South Sudan** | **7914 (5326 , 11400)** | **10.8 (8.5 , 13.3)** | **381.4 (261.8 , 534.7)** | **1124 (590 , 2010)** | **1.6 (0.9 , 2.6)** | **56.9 (32.1 , 95.6)** |
| **United Republic of Tanzania** | **67317 (47367 , 96299)** | **12.8 (10.5 , 15.4)** | **554.2 (399 , 775.8)** | **30389 (18029 , 48143)** | **5.5 (3.5 , 8.2)** | **216 (136.7 , 328.9)** |
| **Uganda** | **28155 (21330 , 35772)** | **7.5 (6.1 , 8.8)** | **425.2 (330.6 , 531.3)** | **8934 (5444 , 13762)** | **2.5 (1.6 , 3.6)** | **111.3 (70.2 , 164.6)** |
| **Zambia** | **18026 (13183 , 23150)** | **10.6 (8.9 , 12.3)** | **548.3 (412.3 , 693.1)** | **4618 (2900 , 7011)** | **2.6 (1.8 , 3.6)** | **139.3 (92.5 , 205.8)** |
| **Central Sub-Saharan Africa** | **122198 (86697 , 177945)** | **13.5 (11.2 , 17)** | **474.1 (338.9 , 686.8)** | **17998 (11410 , 28428)** | **1.8 (1.2 , 2.6)** | **53.8 (34.8 , 82.7)** |
| **Angola** | **37094 (29404 , 47267)** | **17.1 (15.3 , 18.9)** | **686.6 (556.2 , 856)** | **6501 (3996 , 10531)** | **2.9 (2 , 4.4)** | **89 (57.5 , 138.4)** |
| **Central African Republic** | **6024 (3881 , 9381)** | **12.8 (9.9 , 16.2)** | **538.7 (359.9 , 815.4)** | **818 (419 , 1428)** | **1.7 (1 , 2.8)** | **58.1 (31.8 , 99.5)** |
| **Congo** | **7722 (5857 , 10075)** | **16.3 (14.1 , 19.1)** | **586.2 (464.3 , 737.4)** | **863 (509 , 1466)** | **1.5 (1 , 2.3)** | **55.7 (33.7 , 91.6)** |
| **Democratic Republic of the Congo** | **66566 (40406 , 111211)** | **11.8 (8.7 , 16.8)** | **385.7 (236.6 , 643.4)** | **9339 (5209 , 15801)** | **1.4 (0.9 , 2.3)** | **42 (24.1 , 69.5)** |
| **Equatorial Guinea** | **1210 (801 , 1792)** | **14.1 (11.4 , 17.5)** | **593.3 (403.1 , 852.2)** | **138 (73 , 241)** | **1.4 (0.9 , 2.1)** | **45.5 (25.3 , 77.7)** |
| **Gabon** | **3582 (2582 , 4693)** | **15.7 (13.5 , 18.3)** | **676 (504.1 , 879.5)** | **339 (205 , 537)** | **1.8 (1.3 , 2.6)** | **56.6 (35 , 87.1)** |
