# Supplementary material for: Global, regional, and national burden of cancers attributable to tobacco smoking in 204 countries and territories, 1990–2019
Source: Cancer Med. 2022 May 27;11(13):2662–78. doi: 10.1002/cam4.4647 (PMC9249976; doi:10.1002/cam4.4647)
Supplement: Supplementary file 14 — Table S3 [file CAM4-11-2662-s009.doc]

| **Table S3: Number, proportion and age-standardized rates of cancer deaths attributable to smoking (per 100,000) in 1990 and 2019, by location (Generated from data available from http://ghdx.healthdata.org/gbd-results-tool)** | | | | | | | |
| --- | --- | --- | --- | --- | --- | --- | --- |
|  | **1990** | | | **2019** | | | **% change in ASRs per 100,000**  **1990-2019** |
|  | **No**  **(95% UI)** | **PAF**  **(95% UI)** | **ASRs per 100,000 (95% UI)** | **No**  **(95% UI)** | **PAF**  **(95% UI)** | **ASRs per 100,000 (95% UI)** |
| **Global** | **1543971 (1453808 , 1636637)** | **26.8 (25.6 , 28)** | **39.8 (37.3 , 42.1)** | **2493026 (2275657 , 2716575)** | **24.7 (23.5 , 26.1)** | **30.6 (28 , 33.3)** | **-23 (-29.5 , -15.8)** |
| **High-income North America** | **210146 (199291 , 219932)** | **35.4 (34 , 37)** | **60 (57.1 , 62.6)** | **256115 (237465 , 272442)** | **29.5 (28.1 , 31)** | **40 (37.3 , 42.5)** | **-33.2 (-35.8 , -30.3)** |
| **Canada** | **19578 (18575 , 20518)** | **36.3 (34.8 , 38)** | **60.2 (57.1 , 63.1)** | **25550 (23192 , 27549)** | **27.5 (25.9 , 29.1)** | **36.2 (33.1 , 38.9)** | **-39.9 (-43.2 , -36.8)** |
| **Greenland** | **41 (36 , 46)** | **41.7 (39 , 44.6)** | **120.5 (108.5 , 134)** | **67 (54 , 80)** | **42.5 (39.9 , 45.2)** | **97.6 (80.1 , 115.4)** | **-19 (-33.8 , -3.2)** |
| **United States of America** | **190523 (180387 , 199554)** | **35.3 (33.8 , 37)** | **60 (57.1 , 62.7)** | **230494 (213740 , 245396)** | **29.8 (28.3 , 31.3)** | **40.5 (37.8 , 43)** | **-32.5 (-35.1 , -29.5)** |
| **Australasia** | **10623 (10060 , 11179)** | **27.8 (26.4 , 29.2)** | **44.7 (42.3 , 47.1)** | **11523 (10483 , 12420)** | **17.9 (16.9 , 19)** | **23.2 (21.1 , 24.8)** | **-48.2 (-51.1 , -45.6)** |
| **Australia** | **8703 (8249 , 9166)** | **27.7 (26.3 , 29.1)** | **44 (41.6 , 46.3)** | **9418 (8550 , 10178)** | **17.5 (16.4 , 18.7)** | **22.6 (20.6 , 24.3)** | **-48.7 (-51.9 , -45.7)** |
| **New Zealand** | **1920 (1792 , 2052)** | **28.4 (26.8 , 30.1)** | **48.4 (45.2 , 51.7)** | **2104 (1918 , 2296)** | **19.9 (18.6 , 21.4)** | **26.4 (24.2 , 28.7)** | **-45.3 (-48.4 , -41.9)** |
| **High-income Asia Pacific** | **90007 (84083 , 95241)** | **30.5 (28.7 , 32.2)** | **45.1 (42 , 47.7)** | **135234 (119923 , 146880)** | **24.4 (22.9 , 26.1)** | **28.3 (25.5 , 30.4)** | **-37.2 (-40.8 , -34.1)** |
| **Brunei Darussalam** | **55 (46 , 63)** | **25.8 (23.7 , 28)** | **65.6 (55 , 75.3)** | **104 (89 , 121)** | **19.6 (17.6 , 21.7)** | **42.9 (37.3 , 49.5)** | **-34.6 (-45.9 , -20.6)** |
| **Japan** | **75783 (70594 , 80543)** | **31.2 (29.3 , 33)** | **44.5 (41.4 , 47.3)** | **105461 (92982 , 115119)** | **23.9 (22.3 , 25.5)** | **27.8 (24.9 , 29.9)** | **-37.6 (-41.5 , -34.5)** |
| **Singapore** | **925 (859 , 994)** | **27.3 (25.5 , 29.2)** | **44.1 (40.9 , 47.5)** | **1340 (1206 , 1467)** | **19.1 (17.5 , 20.6)** | **17.6 (15.8 , 19.3)** | **-60.1 (-63.6 , -56.6)** |
| **Republic of Korea** | **13244 (12365 , 14346)** | **27.4 (25.5 , 29.3)** | **44.4 (41.4 , 48.5)** | **28330 (25483 , 31303)** | **27.2 (25.3 , 29.3)** | **31.8 (28.5 , 35.1)** | **-28.5 (-35.6 , -20)** |
| **Western Europe** | **301576 (288565 , 315180)** | **30.8 (29.6 , 32.2)** | **52.2 (50 , 54.4)** | **332759 (307650 , 354004)** | **25.8 (24.6 , 27.2)** | **36.8 (34.5 , 38.8)** | **-29.5 (-31.7 , -27.3)** |
| **Andorra** | **33 (26 , 44)** | **32.5 (29.9 , 35.2)** | **59.9 (47.2 , 79.4)** | **60 (46 , 76)** | **26.3 (24.2 , 28.3)** | **42.8 (32.6 , 54.2)** | **-28.6 (-49.6 , -2.8)** |
| **Austria** | **4840 (4553 , 5163)** | **23.4 (22.1 , 24.9)** | **41.3 (38.9 , 43.8)** | **5640 (5234 , 6052)** | **25.3 (24 , 26.7)** | **32.4 (30.3 , 34.6)** | **-21.4 (-25.3 , -17.4)** |
| **Belgium** | **10044 (9484 , 10572)** | **33.1 (31.5 , 34.7)** | **64.9 (61.5 , 68.3)** | **9558 (8829 , 10276)** | **28.2 (26.6 , 29.7)** | **42.2 (39.4 , 45.1)** | **-35 (-38.2 , -31.6)** |
| **Cyprus** | **232 (202 , 257)** | **24.6 (22.4 , 26.7)** | **28.9 (25.7 , 31.9)** | **624 (549 , 709)** | **27.2 (25.5 , 28.9)** | **31.5 (27.7 , 35.7)** | **8.8 (-7 , 27.9)** |
| **Denmark** | **5830 (5506 , 6147)** | **38.5 (36.6 , 40.3)** | **72.3 (68.4 , 76)** | **5701 (5212 , 6201)** | **30.8 (29.1 , 32.8)** | **47.8 (43.8 , 51.7)** | **-33.9 (-38 , -29.3)** |
| **Finland** | **2644 (2492 , 2809)** | **25.6 (24.1 , 27.1)** | **36.7 (34.7 , 39)** | **2896 (2651 , 3138)** | **20 (18.8 , 21.3)** | **23.2 (21.4 , 25)** | **-36.9 (-41.5 , -32.5)** |
| **France** | **41509 (39153 , 43813)** | **28.1 (26.7 , 29.7)** | **51.7 (48.8 , 54.6)** | **48526 (44527 , 52237)** | **24.6 (23 , 26.2)** | **37.2 (34.5 , 39.8)** | **-28.1 (-32.1 , -23.8)** |
| **Germany** | **56878 (53366 , 60491)** | **28.1 (26.4 , 29.7)** | **45.2 (42.6 , 47.9)** | **70086 (64738 , 75075)** | **25.6 (24.2 , 27.2)** | **37.5 (34.8 , 39.8)** | **-17.2 (-21.9 , -12.5)** |
| **Greece** | **8442 (8015 , 8849)** | **35.6 (34 , 37.1)** | **54.7 (51.9 , 57.3)** | **11784 (10838 , 12651)** | **33.5 (31.8 , 35.4)** | **50.3 (46.9 , 53.6)** | **-8 (-12.9 , -2.6)** |
| **Iceland** | **131 (121 , 140)** | **29.8 (28 , 31.5)** | **45.7 (42.3 , 49)** | **166 (147 , 186)** | **25.3 (23.8 , 27)** | **29.6 (26.5 , 33.1)** | **-35.1 (-41.9 , -27.7)** |
| **Ireland** | **2473 (2340 , 2618)** | **33.7 (32 , 35.4)** | **59.3 (56.1 , 62.9)** | **2745 (2519 , 2977)** | **26.4 (25 , 27.9)** | **36 (33.1 , 39)** | **-39.4 (-43.3 , -35.3)** |
| **Israel** | **1772 (1634 , 1906)** | **25 (23.4 , 26.8)** | **36.7 (33.8 , 39.4)** | **3077 (2806 , 3337)** | **21 (19.7 , 22.4)** | **26.3 (24.1 , 28.5)** | **-28.2 (-33 , -22.8)** |
| **Italy** | **47200 (44840 , 49357)** | **31.4 (29.9 , 32.9)** | **52.6 (50 , 55.1)** | **46555 (42233 , 49938)** | **24.2 (22.9 , 25.7)** | **32.2 (29.7 , 34.4)** | **-38.7 (-42.3 , -35.7)** |
| **Luxembourg** | **317 (293 , 344)** | **30.5 (28.5 , 32.9)** | **58 (53.6 , 62.9)** | **346 (299 , 395)** | **26.7 (24.6 , 28.8)** | **34.5 (29.9 , 39.5)** | **-40.4 (-47.9 , -31.8)** |
| **Malta** | **171 (159 , 183)** | **27.8 (26.2 , 29.4)** | **39.5 (36.8 , 42.3)** | **239 (212 , 268)** | **23.9 (22.3 , 25.4)** | **25.7 (22.9 , 28.7)** | **-35 (-42.3 , -26.8)** |
| **Monaco** | **41 (33 , 49)** | **29.3 (27 , 31.8)** | **59.9 (48.1 , 72.6)** | **64 (53 , 77)** | **28.6 (26.1 , 31)** | **68.3 (55.6 , 82.3)** | **14.1 (-11.7 , 50.3)** |
| **Netherlands** | **13907 (13195 , 14602)** | **38.2 (36.4 , 40)** | **69.3 (65.8 , 72.7)** | **16708 (15361 , 18061)** | **29.6 (28 , 31.3)** | **47.6 (44 , 51.2)** | **-31.3 (-35.1 , -27.2)** |
| **Norway** | **2440 (2271 , 2620)** | **23.5 (22 , 25.2)** | **36.1 (33.9 , 38.5)** | **2232 (2037 , 2410)** | **17.6 (16.4 , 18.9)** | **23.2 (21.3 , 25)** | **-35.7 (-40.1 , -31.5)** |
| **Portugal** | **4638 (4308 , 4968)** | **21.4 (19.9 , 23)** | **32.9 (30.7 , 35.2)** | **5920 (5444 , 6372)** | **18.7 (17.4 , 20)** | **26.7 (24.8 , 28.6)** | **-19 (-24.8 , -13.7)** |
| **San Marino** | **17 (15 , 20)** | **27.7 (25.3 , 30.2)** | **51.1 (43.5 , 59.3)** | **25 (17 , 36)** | **21.6 (19.5 , 23.5)** | **39.1 (25.3 , 56.7)** | **-23.5 (-50.6 , 13.9)** |
| **Spain** | **26808 (25407 , 28159)** | **32.3 (30.8 , 33.8)** | **49.4 (46.8 , 51.8)** | **34118 (31483 , 36512)** | **27.1 (25.5 , 28.8)** | **37.3 (34.7 , 39.7)** | **-24.4 (-28.5 , -20.4)** |
| **Sweden** | **4901 (4510 , 5272)** | **22.6 (21.2 , 24.2)** | **32.3 (29.9 , 34.5)** | **5697 (5183 , 6168)** | **21.5 (20.1 , 22.9)** | **25.9 (23.6 , 27.9)** | **-19.8 (-24.4 , -15.4)** |
| **Switzerland** | **4720 (4459 , 4996)** | **30.6 (29.1 , 32.3)** | **46.1 (43.6 , 48.7)** | **5057 (4571 , 5471)** | **25 (23.4 , 26.5)** | **28.8 (26.4 , 30.9)** | **-37.5 (-41.3 , -33.3)** |
| **United Kingdom** | **61339 (58210 , 64329)** | **36.1 (34.5 , 37.8)** | **66.5 (63.2 , 69.7)** | **54645 (50591 , 58570)** | **27.9 (26.4 , 29.5)** | **42.1 (39.3 , 45)** | **-36.7 (-39.4 , -33.8)** |
| **Southern Latin America** | **21459 (20285 , 22589)** | **26.4 (25 , 27.8)** | **46.1 (43.4 , 48.5)** | **27249 (25382 , 29197)** | **21.5 (20.1 , 22.8)** | **32.6 (30.4 , 34.9)** | **-29.2 (-32.7 , -25.8)** |
| **Argentina** | **15978 (15062 , 16895)** | **28.3 (26.7 , 29.9)** | **49 (46.1 , 51.8)** | **19906 (18489 , 21356)** | **23.4 (21.8 , 24.9)** | **36.9 (34.3 , 39.6)** | **-24.7 (-29.4 , -20)** |
| **Chile** | **3142 (2833 , 3435)** | **18.9 (17.2 , 20.7)** | **31.3 (28.2 , 34.1)** | **5022 (4544 , 5485)** | **15.9 (14.6 , 17.2)** | **20.8 (18.8 , 22.7)** | **-33.5 (-39.1 , -27.7)** |
| **Uruguay** | **2339 (2197 , 2482)** | **28.4 (26.9 , 30)** | **59.8 (56.2 , 63.4)** | **2320 (2149 , 2492)** | **23 (21.6 , 24.5)** | **44.6 (41.5 , 47.8)** | **-25.5 (-30.3 , -20.8)** |
| **Eastern Europe** | **126626 (121301 , 132159)** | **29.2 (28 , 30.4)** | **43.8 (42 , 45.7)** | **116035 (100998 , 131039)** | **26.4 (24.3 , 28.5)** | **33.6 (29.2 , 37.9)** | **-23.5 (-32.7 , -13.5)** |
| **Belarus** | **6233 (5871 , 6603)** | **31.5 (29.7 , 33.2)** | **47 (44.2 , 49.8)** | **5512 (4295 , 7002)** | **28 (26.2 , 29.7)** | **34.6 (26.9 , 44.1)** | **-26.5 (-42.6 , -6.2)** |
| **Estonia** | **1076 (1014 , 1143)** | **30.9 (29.2 , 32.7)** | **51.9 (48.9 , 55.1)** | **985 (778 , 1239)** | **25.7 (24.1 , 27.4)** | **38.3 (30 , 48.2)** | **-26.3 (-42.2 , -7.7)** |
| **Latvia** | **1729 (1633 , 1830)** | **30.7 (29.2 , 32.4)** | **47.9 (45.2 , 50.7)** | **1364 (1119 , 1666)** | **24.4 (21.4 , 27.2)** | **35.9 (29.2 , 43.9)** | **-25.1 (-39 , -7.4)** |
| **Lithuania** | **2005 (1886 , 2129)** | **28.6 (27 , 30.1)** | **44 (41.4 , 46.7)** | **1819 (1487 , 2211)** | **23.1 (21.6 , 24.7)** | **33.1 (26.9 , 40.4)** | **-24.9 (-38.6 , -8.3)** |
| **Republic of Moldova** | **1727 (1588 , 1869)** | **27.5 (25.4 , 29.7)** | **37 (34.1 , 40)** | **1619 (1393 , 1867)** | **25.9 (24.2 , 27.5)** | **27.6 (23.7 , 31.7)** | **-25.5 (-36 , -14.7)** |
| **Russian Federation** | **78128 (74326 , 81949)** | **28.3 (26.9 , 29.7)** | **41.6 (39.5 , 43.6)** | **78387 (65498 , 92062)** | **26.4 (23.7 , 29)** | **33 (27.6 , 38.8)** | **-20.6 (-32.8 , -6.7)** |
| **Ukraine** | **35728 (33829 , 37497)** | **30.9 (29.2 , 32.3)** | **49 (46.3 , 51.4)** | **26348 (21408 , 31888)** | **26.5 (23.7 , 29.4)** | **35.3 (28.6 , 42.6)** | **-28 (-41.7 , -12.4)** |
| **Central Europe** | **83595 (80231 , 87060)** | **33.1 (31.8 , 34.3)** | **55.7 (53.4 , 58)** | **107219 (94339 , 122440)** | **31 (29.6 , 32.6)** | **50.4 (44.4 , 57.6)** | **-9.5 (-20.5 , 1.9)** |
| **Albania** | **766 (707 , 829)** | **29.3 (27.2 , 31.3)** | **38.3 (35.3 , 41.5)** | **1467 (1085 , 1951)** | **31.2 (29.2 , 33.3)** | **33.4 (24.8 , 44.4)** | **-12.6 (-34.6 , 15.4)** |
| **Bosnia and Herzegovina** | **1979 (1857 , 2097)** | **34.2 (32.4 , 36)** | **47.3 (44.1 , 50.3)** | **3340 (2633 , 4221)** | **35.2 (33.5 , 37.1)** | **55.1 (43.4 , 69.6)** | **16.6 (-8.9 , 46.8)** |
| **Bulgaria** | **5698 (5278 , 6121)** | **33.3 (31.4 , 35.2)** | **44.4 (41.3 , 47.6)** | **6726 (5350 , 8398)** | **30.5 (28.8 , 32.2)** | **49.6 (39.2 , 62.8)** | **11.8 (-12.3 , 40.3)** |
| **Croatia** | **4331 (4013 , 4706)** | **37.1 (35.2 , 39.1)** | **66.5 (61.7 , 72.2)** | **4143 (3306 , 5199)** | **29.5 (27.8 , 31.4)** | **47.7 (37.9 , 60.3)** | **-28.3 (-43.7 , -9.3)** |
| **Czechia** | **9051 (8467 , 9592)** | **31.8 (30 , 33.6)** | **65.7 (61.6 , 69.6)** | **8641 (7093 , 10527)** | **28.1 (26.6 , 29.7)** | **40.4 (32.9 , 49.3)** | **-38.5 (-49.6 , -25)** |
| **Hungary** | **10193 (9658 , 10719)** | **32.6 (31 , 34.1)** | **69.4 (65.8 , 73)** | **11401 (9325 , 13883)** | **33.2 (31.6 , 34.9)** | **61.3 (50.1 , 74.6)** | **-11.7 (-27.1 , 6.7)** |
| **Montenegro** | **369 (325 , 411)** | **38.7 (36.1 , 41.3)** | **58.3 (51.5 , 65)** | **687 (575 , 826)** | **41.8 (39.5 , 44.1)** | **68.4 (57.4 , 82.4)** | **17.4 (-4 , 42.9)** |
| **North Macedonia** | **903 (816 , 987)** | **31.4 (29 , 33.6)** | **47.1 (42.5 , 51.3)** | **1851 (1455 , 2350)** | **34.1 (32.1 , 36)** | **56.1 (44.2 , 71.1)** | **19.3 (-6.1 , 51.4)** |
| **Poland** | **29025 (27774 , 30286)** | **34.8 (33.3 , 36.3)** | **65.7 (62.9 , 68.6)** | **39285 (32477 , 46828)** | **32.2 (30.1 , 34.4)** | **55.7 (46.1 , 66.6)** | **-15.1 (-29.3 , 0.5)** |
| **Romania** | **10479 (9842 , 11133)** | **29.8 (28.1 , 31.4)** | **36.1 (33.9 , 38.3)** | **14972 (12287 , 18109)** | **28.5 (27 , 30)** | **42.6 (34.8 , 51.8)** | **18.1 (-3.2 , 45)** |
| **Serbia** | **6208 (5240 , 6720)** | **32.8 (30.7 , 34.8)** | **52 (43.9 , 56.2)** | **9569 (7605 , 11911)** | **33.6 (32 , 35.6)** | **60.2 (47.6 , 75.5)** | **15.8 (-9.6 , 49.4)** |
| **Slovakia** | **3388 (3165 , 3634)** | **32.1 (30.4 , 33.9)** | **56.5 (52.9 , 60.5)** | **3507 (2730 , 4433)** | **25.2 (23.4 , 27)** | **37.6 (29.2 , 47.6)** | **-33.5 (-47.9 , -15.5)** |
| **Slovenia** | **1204 (919 , 1562)** | **29.1 (27.5 , 30.7)** | **48.9 (37.3 , 63.4)** | **1632 (1270 , 2112)** | **25.7 (24.1 , 27.6)** | **38.9 (30.2 , 50.5)** | **-20.5 (-45.8 , 12.3)** |
| **Central Asia** | **17515 (16660 , 18351)** | **25.6 (24.5 , 26.7)** | **36.2 (34.4 , 37.9)** | **18262 (16349 , 20377)** | **20.6 (19.4 , 21.8)** | **24.9 (22.5 , 27.6)** | **-31.3 (-37.7 , -23.7)** |
| **Armenia** | **1329 (1260 , 1399)** | **32.1 (30.7 , 33.6)** | **46.4 (44.1 , 48.9)** | **1646 (1380 , 1928)** | **28.2 (26.8 , 29.7)** | **38.9 (32.6 , 45.4)** | **-16.2 (-29.9 , -0.4)** |
| **Azerbaijan** | **1798 (1610 , 1995)** | **25.9 (23.8 , 27.8)** | **34.2 (30.7 , 37.9)** | **3062 (2369 , 3901)** | **25.7 (22.3 , 29.2)** | **32.3 (25.3 , 40.8)** | **-5.3 (-24.7 , 20.8)** |
| **Georgia** | **2246 (2022 , 2494)** | **27.5 (25.7 , 29.2)** | **34.7 (31.4 , 38.5)** | **2212 (1847 , 2587)** | **26.6 (25.2 , 28.2)** | **37.9 (31.6 , 44.6)** | **9.3 (-10.2 , 31.2)** |
| **Kazakhstan** | **6696 (6196 , 7200)** | **29.2 (27.3 , 31.1)** | **50.9 (47.2 , 54.7)** | **4868 (4157 , 5603)** | **23.1 (21.7 , 24.5)** | **27.4 (23.5 , 31.5)** | **-46.2 (-54.3 , -37.7)** |
| **Kyrgyzstan** | **1127 (1047 , 1210)** | **27.4 (26.1 , 28.8)** | **36.5 (34.1 , 39.2)** | **875 (758 , 1006)** | **21.1 (19.7 , 22.6)** | **19.2 (16.7 , 22)** | **-47.3 (-54.4 , -39.9)** |
| **Mongolia** | **594 (473 , 724)** | **20.4 (17.6 , 23.1)** | **58.7 (47 , 71)** | **1180 (897 , 1542)** | **19.8 (16.6 , 23)** | **56.4 (43.5 , 72)** | **-3.8 (-26.6 , 25.4)** |
| **Tajikistan** | **827 (738 , 922)** | **22.8 (20.8 , 24.8)** | **29.2 (26.1 , 32.7)** | **739 (582 , 972)** | **13.8 (12.5 , 15.8)** | **16.4 (13.2 , 21.1)** | **-44 (-55 , -28.2)** |
| **Turkmenistan** | **675 (636 , 716)** | **26.2 (24.6 , 27.8)** | **33.9 (32 , 36)** | **624 (492 , 788)** | **17.2 (16 , 18.3)** | **15.3 (12.2 , 19.2)** | **-54.8 (-63.9 , -44.1)** |
| **Uzbekistan** | **2224 (1981 , 2484)** | **17.2 (15.3 , 19.1)** | **19.2 (17.1 , 21.4)** | **3055 (2496 , 3639)** | **13.7 (12.6 , 14.9)** | **14.6 (12.1 , 17.2)** | **-23.9 (-36.8 , -8.6)** |
| **Central Latin America** | **16494 (15124 , 17843)** | **17 (15.6 , 18.4)** | **21 (19.3 , 22.8)** | **25489 (21490 , 30272)** | **11 (10 , 11.9)** | **11.1 (9.4 , 13.2)** | **-47.3 (-54.9 , -38.4)** |
| **Colombia** | **3938 (3545 , 4360)** | **16.9 (15.3 , 18.6)** | **23.4 (21 , 25.9)** | **5201 (3959 , 6819)** | **10.5 (9.4 , 11.6)** | **9.9 (7.5 , 13)** | **-57.6 (-67.2 , -44.9)** |
| **Costa Rica** | **410 (368 , 454)** | **17.5 (15.9 , 19.1)** | **24.4 (21.8 , 27)** | **719 (560 , 919)** | **11.9 (10.8 , 13)** | **14.2 (11.1 , 18.1)** | **-41.8 (-54.7 , -25.6)** |
| **El Salvador** | **244 (212 , 281)** | **9.4 (8.3 , 10.7)** | **8.4 (7.3 , 9.6)** | **474 (347 , 629)** | **8 (7 , 9.1)** | **8 (5.9 , 10.7)** | **-4.3 (-28.8 , 26.5)** |
| **Guatemala** | **364 (298 , 438)** | **9.2 (7.7 , 10.9)** | **11.3 (9.3 , 13.4)** | **905 (670 , 1190)** | **7.2 (6.2 , 8.3)** | **8.6 (6.4 , 11.2)** | **-24.1 (-42.8 , 0.3)** |
| **Honduras** | **302 (238 , 366)** | **13.7 (11.8 , 15.6)** | **15.3 (12 , 18.6)** | **1040 (767 , 1350)** | **13.4 (10.7 , 16.1)** | **18.1 (13.4 , 23.3)** | **18.1 (-10.1 , 53.7)** |
| **Mexico** | **8304 (7486 , 9171)** | **17.6 (15.9 , 19.4)** | **21.2 (19.1 , 23.4)** | **11197 (9270 , 13463)** | **10.5 (9.3 , 11.8)** | **10 (8.3 , 12)** | **-52.7 (-60.3 , -43.9)** |
| **Nicaragua** | **171 (150 , 194)** | **11.6 (10.3 , 13)** | **11.8 (10.5 , 13.3)** | **396 (312 , 483)** | **8.9 (7.9 , 10)** | **9.7 (7.7 , 11.8)** | **-17.8 (-34.2 , 0)** |
| **Panama** | **269 (243 , 297)** | **16.6 (15.3 , 18.1)** | **18.6 (16.8 , 20.5)** | **399 (303 , 520)** | **10 (9.1 , 11)** | **9.7 (7.3 , 12.6)** | **-47.9 (-59.8 , -33.2)** |
| **Venezuela (Bolivarian Republic of)** | **2493 (2261 , 2720)** | **19.9 (18.1 , 21.6)** | **26.7 (24.2 , 29.1)** | **5157 (3838 , 6764)** | **14.8 (13.4 , 16.5)** | **18 (13.5 , 23.4)** | **-32.5 (-49.3 , -12.4)** |
| **Andean Latin America** | **2266 (1942 , 2614)** | **8.3 (7.3 , 9.4)** | **11.7 (10 , 13.4)** | **4229 (3342 , 5216)** | **6.5 (5.7 , 7.3)** | **7.8 (6.2 , 9.6)** | **-33.6 (-45.7 , -19.7)** |
| **Bolivia (Plurinational State of)** | **601 (454 , 749)** | **10.9 (9.2 , 12.6)** | **19.8 (15.1 , 24.5)** | **1099 (780 , 1472)** | **7.8 (6.5 , 9.1)** | **13.1 (9.3 , 17.4)** | **-33.9 (-49.4 , -14)** |
| **Ecuador** | **710 (614 , 799)** | **11.4 (10 , 12.8)** | **14.3 (12.4 , 16)** | **1547 (1203 , 1980)** | **8.8 (7.8 , 9.8)** | **10.8 (8.4 , 13.7)** | **-24.6 (-40.5 , -3.2)** |
| **Peru** | **956 (779 , 1176)** | **6.2 (5.3 , 7.1)** | **8.4 (6.9 , 10.4)** | **1582 (1126 , 2167)** | **4.7 (4 , 5.5)** | **5 (3.6 , 6.9)** | **-40.5 (-57.5 , -16.2)** |
| **Caribbean** | **7558 (7050 , 8055)** | **21.3 (19.9 , 22.7)** | **29.6 (27.5 , 31.5)** | **13805 (11718 , 16123)** | **19.8 (18.1 , 21.5)** | **26.6 (22.6 , 31.1)** | **-9.9 (-23 , 4.2)** |
| **Antigua and Barbuda** | **7 (6 , 8)** | **9.5 (8.3 , 10.9)** | **13.4 (11.7 , 15.2)** | **13 (11 , 16)** | **9.5 (8.4 , 10.8)** | **13.4 (11.2 , 16.1)** | **0 (-16.7 , 18.7)** |
| **Barbados** | **42 (37 , 48)** | **9.2 (8.2 , 10.3)** | **14.4 (12.6 , 16.2)** | **62 (50 , 75)** | **7.5 (6.7 , 8.3)** | **12.4 (10.1 , 15.2)** | **-13.6 (-28.6 , 4.3)** |
| **Belize** | **13 (11 , 14)** | **13.5 (11.9 , 14.9)** | **13.8 (12.1 , 15.7)** | **40 (34 , 46)** | **13.4 (12.1 , 14.7)** | **15.3 (12.9 , 17.6)** | **10.3 (-6.7 , 30.5)** |
| **Bermuda** | **23 (19 , 26)** | **18.2 (15.8 , 20.8)** | **36.5 (31.5 , 41.9)** | **31 (25 , 37)** | **17.1 (15.4 , 18.9)** | **23 (19.1 , 28.1)** | **-36.9 (-48.2 , -22.7)** |
| **Bahamas** | **31 (26 , 35)** | **11.7 (10.2 , 13.4)** | **20.4 (17.5 , 23.5)** | **58 (46 , 73)** | **10.1 (8.9 , 11.3)** | **15.3 (12.2 , 19)** | **-25.1 (-40.8 , -6)** |
| **Cuba** | **4623 (4314 , 4887)** | **31.1 (29.3 , 32.9)** | **45 (42.1 , 47.6)** | **8276 (6746 , 9994)** | **29.8 (28 , 32.2)** | **43.2 (35.1 , 52.2)** | **-4 (-21 , 15.9)** |
| **Dominica** | **16 (14 , 18)** | **11.3 (10 , 12.6)** | **22.1 (19.1 , 25.4)** | **17 (14 , 21)** | **9.6 (8.4 , 10.8)** | **19.1 (15.3 , 23.5)** | **-13.7 (-30.4 , 6.8)** |
| **Dominican Republic** | **602 (517 , 698)** | **17.9 (15.5 , 20.2)** | **17.6 (15.1 , 20.4)** | **2061 (1542 , 2714)** | **19.1 (17.2 , 20.9)** | **23.2 (17.5 , 30.3)** | **31.3 (-2.7 , 72.7)** |
| **Grenada** | **14 (12 , 15)** | **10.1 (8.9 , 11.4)** | **18.9 (16.4 , 21.6)** | **19 (17 , 22)** | **10.3 (9.1 , 11.5)** | **17.1 (14.9 , 19.5)** | **-9.5 (-21.9 , 4.7)** |
| **Guyana** | **51 (43 , 61)** | **10 (8.8 , 11.3)** | **13.9 (11.6 , 16.4)** | **69 (53 , 90)** | **8.9 (7.8 , 10)** | **11.2 (8.6 , 14.5)** | **-19.4 (-38.3 , 5.6)** |
| **Haiti** | **492 (302 , 661)** | **8.4 (6.8 , 10.2)** | **14.9 (9.3 , 19.9)** | **634 (409 , 899)** | **6 (5 , 7.3)** | **9.4 (6.1 , 13.1)** | **-36.8 (-53 , -15.2)** |
| **Jamaica** | **347 (313 , 383)** | **17 (15.5 , 18.7)** | **19.7 (17.8 , 21.6)** | **628 (499 , 790)** | **14.5 (13.1 , 16.2)** | **21.3 (16.9 , 26.8)** | **8.1 (-14.6 , 34.3)** |
| **Puerto Rico** | **778 (687 , 873)** | **17.2 (15.3 , 19.3)** | **21.4 (18.9 , 24)** | **958 (732 , 1224)** | **13.8 (12.2 , 15.4)** | **13.1 (9.8 , 16.8)** | **-39 (-52.9 , -20.8)** |
| **Saint Kitts and Nevis** | **6 (5 , 7)** | **7.5 (6.4 , 8.7)** | **15.1 (12.9 , 17.8)** | **8 (7 , 10)** | **8.5 (7.6 , 9.5)** | **13 (10.8 , 15.5)** | **-13.8 (-28.4 , 3.9)** |
| **Saint Lucia** | **19 (17 , 21)** | **13 (11.6 , 14.4)** | **22.2 (19.7 , 24.8)** | **36 (30 , 43)** | **11.8 (10.6 , 13)** | **16.7 (13.9 , 20)** | **-24.4 (-37.3 , -10.3)** |
| **Saint Vincent and the Grenadines** | **10 (9 , 12)** | **9.2 (8.1 , 10.4)** | **14.4 (12.5 , 16.6)** | **19 (16 , 23)** | **9.2 (8 , 10.4)** | **14.2 (12 , 16.8)** | **-1.9 (-17.3 , 15.8)** |
| **Suriname** | **48 (42 , 55)** | **16.3 (14.5 , 18.3)** | **18.9 (16.6 , 21.5)** | **117 (95 , 142)** | **16.4 (14.8 , 18.2)** | **19.7 (16 , 23.7)** | **3.9 (-14.6 , 26.6)** |
| **Trinidad and Tobago** | **169 (153 , 187)** | **14.9 (13.4 , 16.4)** | **20.5 (18.4 , 22.6)** | **248 (184 , 327)** | **12.3 (11 , 13.7)** | **13.3 (9.8 , 17.4)** | **-35.3 (-51.7 , -15.5)** |
| **United States Virgin Islands** | **15 (13 , 19)** | **12.2 (10.3 , 14.2)** | **18.7 (15.4 , 22.6)** | **42 (35 , 50)** | **12.9 (11.2 , 14.6)** | **22.2 (18 , 26.4)** | **18.4 (-5.3 , 49.2)** |
| **Tropical Latin America** | **33621 (31280 , 36035)** | **28.1 (26.1 , 30)** | **38 (35.3 , 40.7)** | **53293 (49144 , 57412)** | **19.6 (18.4 , 20.8)** | **22.2 (20.4 , 23.9)** | **-41.7 (-45 , -37.9)** |
| **Brazil** | **33133 (30838 , 35477)** | **28.2 (26.2 , 30.1)** | **38.4 (35.7 , 41.1)** | **51973 (48064 , 55992)** | **19.5 (18.3 , 20.7)** | **22.1 (20.4 , 23.8)** | **-42.4 (-45.7 , -38.7)** |
| **Paraguay** | **488 (419 , 563)** | **23.9 (21.4 , 26.4)** | **22.9 (19.7 , 26.4)** | **1320 (1006 , 1712)** | **21.5 (19.5 , 23.6)** | **24.8 (19 , 31.9)** | **8.1 (-18.2 , 40.2)** |
| **East Asia** | **396273 (330289 , 463942)** | **26.3 (23.5 , 28.9)** | **46.6 (39.3 , 54.1)** | **924474 (758790 , 1114252)** | **32.8 (30 , 35.5)** | **45 (37.1 , 54)** | **-3.4 (-23.9 , 23.2)** |
| **China** | **385641 (319934 , 452960)** | **26.4 (23.6 , 29)** | **47.1 (39.6 , 54.8)** | **900362 (734338 , 1090262)** | **33.1 (30.2 , 35.9)** | **45.5 (37.3 , 54.8)** | **-3.5 (-24.6 , 23.7)** |
| **Democratic People's Republic of Korea** | **4940 (3794 , 6430)** | **20.8 (18.6 , 23.2)** | **30 (23.5 , 38.1)** | **10467 (8441 , 12715)** | **25.6 (23.1 , 28.3)** | **32.2 (26.1 , 38.9)** | **7.4 (-15.3 , 33.7)** |
| **Taiwan (Province of China)** | **5692 (5313 , 6049)** | **27.6 (25.9 , 29.3)** | **35.5 (33.1 , 37.7)** | **13645 (10672 , 17680)** | **23.9 (22.6 , 25.5)** | **34.2 (26.7 , 44.3)** | **-3.5 (-24.6 , 26.5)** |
| **Southeast Asia** | **63106 (57095 , 69234)** | **21.7 (20.2 , 23.2)** | **26.1 (23.6 , 28.6)** | **140857 (122852 , 162001)** | **21.2 (19.5 , 22.9)** | **24.4 (21.3 , 28.1)** | **-6.5 (-19.6 , 9)** |
| **Cambodia** | **1468 (1206 , 1763)** | **23.7 (20.4 , 27.2)** | **35.4 (29.4 , 42)** | **3497 (2765 , 4116)** | **24 (22 , 26.5)** | **32 (25.6 , 37.6)** | **-9.6 (-29.4 , 12.6)** |
| **Indonesia** | **16142 (13923 , 18442)** | **16.9 (14.8 , 19.7)** | **17.8 (15.3 , 20.3)** | **47840 (37860 , 59222)** | **20.9 (17.5 , 24.3)** | **24.3 (19.5 , 29.6)** | **36.3 (7.4 , 70.4)** |
| **Lao People's Democratic Republic** | **736 (559 , 974)** | **24.1 (20.4 , 27.4)** | **36.4 (27.9 , 47.4)** | **1078 (821 , 1345)** | **21.7 (19.6 , 23.7)** | **27.2 (20.9 , 33.5)** | **-25.3 (-44.8 , -1.8)** |
| **Malaysia** | **2180 (1948 , 2417)** | **19.5 (17.8 , 21.1)** | **25.5 (22.6 , 28.3)** | **5781 (4563 , 7235)** | **18.5 (17 , 20)** | **23.1 (18.4 , 28.8)** | **-9.1 (-29.7 , 15.6)** |
| **Maldives** | **18 (14 , 23)** | **19.5 (16.6 , 22.6)** | **25.6 (20.2 , 31)** | **41 (34 , 49)** | **19.3 (17.7 , 20.8)** | **15.8 (12.9 , 18.8)** | **-38.3 (-53.5 , -16.2)** |
| **Mauritius** | **137 (127 , 148)** | **20.6 (19.2 , 22.1)** | **19.4 (17.9 , 20.8)** | **239 (194 , 295)** | **15.8 (14.7 , 16.8)** | **13.8 (11.3 , 17)** | **-28.6 (-42.1 , -13.2)** |
| **Myanmar** | **8341 (6225 , 11311)** | **24.8 (21.3 , 28.3)** | **37.8 (28.4 , 50.4)** | **10617 (8307 , 13593)** | **20.1 (17.5 , 23.1)** | **24.8 (19.5 , 31.4)** | **-34.5 (-50 , -8.1)** |
| **Philippines** | **8730 (7390 , 9950)** | **23.2 (21 , 25.4)** | **31.3 (26.6 , 35.4)** | **16642 (13112 , 20816)** | **20.5 (17.8 , 23.2)** | **22.5 (17.9 , 27.9)** | **-27.9 (-44 , -6)** |
| **Sri Lanka** | **1351 (1186 , 1517)** | **16 (14.2 , 17.6)** | **13.7 (12.1 , 15.4)** | **2639 (1939 , 3554)** | **14.1 (12.7 , 15.4)** | **10.5 (7.8 , 14)** | **-23.7 (-43.3 , 2.6)** |
| **Seychelles** | **20 (18 , 23)** | **22.9 (20.7 , 25.3)** | **36 (31.2 , 40.3)** | **37 (32 , 43)** | **20.4 (18.6 , 22.4)** | **35 (30.4 , 40.6)** | **-2.8 (-16.3 , 14.5)** |
| **Thailand** | **13215 (11405 , 15168)** | **27.1 (24.6 , 29.5)** | **38.9 (33.7 , 44.6)** | **24991 (18207 , 32851)** | **21.6 (19.5 , 23.7)** | **24.6 (17.9 , 32.2)** | **-36.8 (-53.7 , -15.9)** |
| **Timor-Leste** | **49 (37 , 64)** | **16.7 (13.4 , 21.4)** | **19.8 (15.2 , 25.8)** | **163 (112 , 206)** | **19.8 (16.8 , 22.9)** | **21.2 (14.9 , 26.4)** | **7.2 (-21 , 45.8)** |
| **Viet Nam** | **10635 (8386 , 12999)** | **23.7 (21.9 , 25.5)** | **26.5 (21 , 32.2)** | **27106 (21533 , 33219)** | **24.1 (22.2 , 26.2)** | **29.2 (23.4 , 35.3)** | **10.1 (-16.1 , 46.6)** |
| **Oceania** | **658 (532 , 836)** | **19 (16.5 , 21.7)** | **23.3 (19.1 , 29.4)** | **1413 (1086 , 1863)** | **16.1 (14 , 18.5)** | **21.4 (16.8 , 27.6)** | **-8.2 (-24.6 , 11.3)** |
| **American Samoa** | **7 (6 , 8)** | **22.4 (20.1 , 24.9)** | **33.1 (28.3 , 37.9)** | **13 (11 , 15)** | **19.5 (17.4 , 21.6)** | **28.9 (24.5 , 33.1)** | **-12.7 (-27.6 , 2.3)** |
| **Cook Islands** | **5 (4 , 5)** | **23.9 (20.9 , 27)** | **37.6 (31.9 , 44)** | **7 (6 , 8)** | **21.4 (19.1 , 23.9)** | **27.2 (22.7 , 32.4)** | **-27.8 (-41.2 , -12.2)** |
| **Micronesia (Federated States of)** | **17 (13 , 23)** | **23.5 (20.2 , 27.2)** | **37.9 (28.8 , 48.9)** | **26 (17 , 36)** | **23.3 (19.7 , 27.1)** | **38.3 (26.3 , 52.5)** | **1.1 (-29.8 , 34.8)** |
| **Fiji** | **70 (55 , 86)** | **16.8 (14.6 , 19)** | **20.4 (16.4 , 24.8)** | **105 (80 , 137)** | **13 (11.4 , 14.6)** | **14.7 (11.3 , 18.8)** | **-28 (-45.4 , -2.6)** |
| **Guam** | **23 (19 , 28)** | **26.1 (23 , 29.3)** | **32.4 (26.5 , 38.6)** | **48 (39 , 58)** | **23.3 (21.1 , 25.8)** | **25.5 (20.7 , 30.7)** | **-21.5 (-37.8 , -0.3)** |
| **Kiribati** | **21 (16 , 26)** | **27.5 (23.5 , 31.4)** | **55.7 (43.8 , 67.9)** | **36 (27 , 46)** | **28 (24.4 , 32.1)** | **53.8 (41.4 , 68)** | **-3.5 (-26.2 , 21.4)** |
| **Marshall Islands** | **4 (3 , 6)** | **17.3 (13.5 , 22.2)** | **28.4 (19.9 , 40.2)** | **9 (6 , 13)** | **16.5 (13.5 , 20.5)** | **28.1 (19.7 , 39.6)** | **-1.2 (-23.4 , 27)** |
| **Nauru** | **2 (1 , 2)** | **23.1 (19.8 , 26.3)** | **47 (34.9 , 60.6)** | **2 (1 , 2)** | **21.3 (17.8 , 24.7)** | **42.9 (31 , 55.2)** | **-8.7 (-27.9 , 15.3)** |
| **Niue** | **1 (1 , 1)** | **20.1 (17.5 , 23.1)** | **28 (23 , 34.3)** | **1 (0 , 1)** | **19.9 (17.5 , 22.7)** | **26.9 (22.1 , 32.7)** | **-4.1 (-25.9 , 21.9)** |
| **Northern Mariana Islands** | **8 (7 , 10)** | **26.2 (23.2 , 29.6)** | **54 (46.5 , 63.9)** | **21 (17 , 24)** | **25.8 (23.2 , 28.7)** | **42.1 (36.1 , 48.2)** | **-22 (-34.2 , -9.9)** |
| **Palau** | **4 (3 , 5)** | **20.6 (17.7 , 23.8)** | **38.7 (30.7 , 49.8)** | **7 (5 , 9)** | **19.4 (17 , 22.2)** | **33.1 (26.2 , 42)** | **-14.6 (-35.8 , 11.8)** |
| **Papua New Guinea** | **351 (253 , 476)** | **17.6 (14.4 , 21.2)** | **19.9 (14.3 , 27.1)** | **863 (605 , 1208)** | **15.3 (12.6 , 18.4)** | **19.6 (14.1 , 27.3)** | **-1.4 (-25.9 , 30.3)** |
| **Samoa** | **20 (17 , 24)** | **20 (18 , 22)** | **23.6 (19.4 , 28)** | **29 (24 , 36)** | **18.7 (16.3 , 21)** | **20.5 (17 , 25.1)** | **-13.1 (-29.7 , 9)** |
| **Solomon Islands** | **53 (34 , 75)** | **23.2 (19.5 , 28.3)** | **39.8 (27.4 , 55.8)** | **116 (78 , 167)** | **17.7 (14.6 , 21.5)** | **39.6 (28.2 , 55.7)** | **-0.4 (-24.1 , 29.6)** |
| **Tokelau** | **0 (0 , 0)** | **20.3 (16.9 , 23.8)** | **25.5 (20.2 , 32.2)** | **0 (0 , 0)** | **19.4 (16.6 , 22.1)** | **23.7 (18.7 , 30.4)** | **-7 (-27.2 , 19.6)** |
| **Tonga** | **20 (16 , 25)** | **23.7 (20.8 , 27)** | **38.6 (31.3 , 47.4)** | **27 (22 , 33)** | **21.3 (19 , 23.5)** | **35 (28.9 , 42.4)** | **-9.1 (-27.5 , 15.5)** |
| **Tuvalu** | **2 (2 , 3)** | **21.9 (18.9 , 26)** | **31.4 (24.7 , 42.2)** | **3 (2 , 4)** | **21 (18.5 , 23.8)** | **28.7 (22.1 , 37.5)** | **-8.7 (-31.9 , 20.9)** |
| **Vanuatu** | **14 (10 , 19)** | **18.3 (14.9 , 22.5)** | **23.6 (16.5 , 32.3)** | **34 (25 , 48)** | **15.2 (12.8 , 18.3)** | **21.6 (15.7 , 29.6)** | **-8.3 (-30.7 , 26)** |
| **North Africa and Middle East** | **41059 (35752 , 46531)** | **22.6 (20.9 , 24.3)** | **24.7 (21.5 , 27.8)** | **91978 (81237 , 104314)** | **21.9 (20.6 , 23.2)** | **22.4 (19.8 , 25.4)** | **-9.2 (-22.3 , 8.7)** |
| **Afghanistan** | **836 (560 , 1222)** | **7.2 (5.5 , 9.1)** | **12.2 (8.4 , 17.6)** | **1720 (1228 , 2381)** | **8.1 (6.6 , 9.8)** | **15 (11 , 20.1)** | **22.4 (-9.7 , 65)** |
| **Algeria** | **2022 (1607 , 2509)** | **19.1 (17.4 , 20.6)** | **19.3 (15.7 , 23.5)** | **4282 (3384 , 5374)** | **18 (16.4 , 19.5)** | **14.2 (11.3 , 17.7)** | **-26.5 (-43.7 , -4.2)** |
| **Bahrain** | **70 (59 , 81)** | **27.4 (24.8 , 29.7)** | **52 (44.4 , 59.8)** | **155 (118 , 197)** | **18.9 (16.8 , 21.2)** | **24.2 (19 , 30.1)** | **-53.4 (-64.4 , -38.9)** |
| **Egypt** | **4024 (3444 , 4569)** | **17 (14.7 , 19.2)** | **14 (12.1 , 15.9)** | **11843 (8498 , 16141)** | **20.4 (17.7 , 23.1)** | **19.1 (14 , 25.8)** | **36.5 (1.7 , 84.9)** |
| **Iran (Islamic Republic of)** | **4694 (4003 , 5385)** | **17.6 (15.9 , 19.5)** | **19 (16.1 , 21.6)** | **11111 (10148 , 12200)** | **16.6 (15.4 , 18)** | **16.1 (14.7 , 17.8)** | **-14.9 (-26.6 , 7.1)** |
| **Iraq** | **1861 (1500 , 2272)** | **23.4 (20.5 , 26)** | **25.2 (20.4 , 30.8)** | **5405 (4153 , 6459)** | **22.2 (20.6 , 23.8)** | **26.2 (20.5 , 30.9)** | **3.9 (-20.8 , 33.1)** |
| **Jordan** | **294 (245 , 351)** | **21.2 (19.2 , 23.3)** | **24.1 (20 , 28.7)** | **1307 (1038 , 1589)** | **23.1 (20.2 , 25.8)** | **22.4 (18 , 27.2)** | **-7 (-27.9 , 19.5)** |
| **Kuwait** | **93 (83 , 104)** | **18.5 (16.9 , 20.3)** | **17.2 (15.1 , 19.3)** | **304 (241 , 371)** | **18.4 (16.3 , 20.4)** | **14.7 (11.8 , 17.9)** | **-14.4 (-30 , 5.1)** |
| **Lebanon** | **802 (655 , 976)** | **27.3 (24.7 , 29.8)** | **36.7 (30.1 , 44.2)** | **2244 (1872 , 2938)** | **29.9 (27.6 , 32.7)** | **43.2 (36 , 56.5)** | **17.6 (-8.5 , 65.6)** |
| **Libya** | **494 (383 , 616)** | **24.3 (21.5 , 26.7)** | **27.9 (21.6 , 34.4)** | **1187 (904 , 1493)** | **21.8 (19.5 , 23.7)** | **24.7 (18.9 , 30.9)** | **-11.3 (-34 , 25.1)** |
| **Morocco** | **2368 (1776 , 2851)** | **21.4 (18.7 , 24)** | **17.4 (12.9 , 20.9)** | **5207 (3795 , 6519)** | **18.6 (16.5 , 20.5)** | **16.7 (12.1 , 20.7)** | **-4.1 (-29.2 , 23.6)** |
| **Palestine** | **230 (178 , 290)** | **20.5 (18.2 , 23)** | **28.4 (22 , 35.7)** | **585 (496 , 683)** | **20.1 (18.6 , 21.6)** | **27.4 (23.3 , 31.8)** | **-3.5 (-26.9 , 27.5)** |
| **Oman** | **79 (59 , 101)** | **14 (12.1 , 15.9)** | **12.8 (9.7 , 16.1)** | **129 (104 , 163)** | **9.4 (8.2 , 10.8)** | **9.6 (8 , 11.4)** | **-25.5 (-43.6 , 3.2)** |
| **Qatar** | **22 (17 , 29)** | **16.6 (14.1 , 19.4)** | **26.1 (20.5 , 32.3)** | **135 (97 , 181)** | **16.2 (14.2 , 18.1)** | **26.2 (20.1 , 33)** | **0.4 (-28 , 40.1)** |
| **Saudi Arabia** | **530 (393 , 681)** | **12.5 (10.5 , 14.4)** | **9.6 (7.3 , 12.2)** | **1675 (1298 , 2096)** | **12.8 (11.2 , 14.3)** | **10.3 (8.2 , 12.5)** | **6.7 (-22.3 , 52.6)** |
| **Sudan** | **1274 (823 , 1808)** | **15.1 (12.2 , 18.6)** | **14.3 (9.4 , 20.1)** | **2530 (1692 , 3498)** | **14.8 (12.6 , 17.7)** | **14.9 (10.1 , 20.2)** | **3.9 (-21.4 , 41.5)** |
| **Syrian Arab Republic** | **934 (702 , 1193)** | **20.1 (17.1 , 23.1)** | **18.9 (14.3 , 23.9)** | **1914 (1413 , 2528)** | **21.8 (19.6 , 23.9)** | **16.7 (12.6 , 21.8)** | **-11.5 (-36.5 , 27.8)** |
| **Tunisia** | **1316 (1088 , 1554)** | **32.3 (29.9 , 35.4)** | **27.5 (22.9 , 32.5)** | **3148 (2285 , 4334)** | **31.1 (28.6 , 33.7)** | **25.5 (18.6 , 34.9)** | **-7.2 (-36 , 30.8)** |
| **Turkey** | **18061 (14994 , 21493)** | **33.2 (30.4 , 36.2)** | **50.4 (42.3 , 59.4)** | **33662 (26754 , 41494)** | **32.1 (30.4 , 33.9)** | **38.1 (30.4 , 46.8)** | **-24.4 (-43.4 , -1.1)** |
| **United Arab Emirates** | **96 (69 , 124)** | **14.9 (12.5 , 17.4)** | **28.8 (20.5 , 35.8)** | **844 (601 , 1143)** | **15.5 (13.3 , 17.5)** | **27.1 (21.5 , 33.5)** | **-5.8 (-30.2 , 37.7)** |
| **Yemen** | **933 (677 , 1284)** | **20.5 (17.5 , 23.6)** | **19.5 (14.3 , 26.2)** | **2496 (1853 , 3354)** | **19.7 (17.7 , 22.2)** | **20.2 (15.2 , 27)** | **3.8 (-22.4 , 41.5)** |
| **South Asia** | **95617 (82952 , 106612)** | **18.6 (16.8 , 20.3)** | **18.3 (15.7 , 20.4)** | **189502 (162298 , 220845)** | **15.3 (13.7 , 16.9)** | **14 (12.1 , 16.3)** | **-23.3 (-35.5 , -7)** |
| **Bangladesh** | **10357 (8588 , 12041)** | **19.4 (17 , 21.9)** | **23.3 (19.5 , 27.1)** | **17693 (12893 , 24228)** | **16.6 (14.5 , 18.8)** | **14.1 (10.3 , 19.2)** | **-39.6 (-55 , -19.9)** |
| **Bhutan** | **26 (19 , 34)** | **12.3 (10.2 , 14.8)** | **11 (8 , 14.2)** | **56 (42 , 71)** | **11.8 (10 , 13.8)** | **10.6 (8 , 13.3)** | **-4.1 (-30.9 , 35.1)** |
| **India** | **66359 (56363 , 74943)** | **17.6 (15.7 , 19.5)** | **15.9 (13.5 , 17.9)** | **138980 (114973 , 167711)** | **14.9 (13.1 , 16.8)** | **12.7 (10.6 , 15.2)** | **-20.1 (-35.8 , 0.6)** |
| **Nepal** | **1730 (1330 , 2202)** | **18.2 (15.4 , 21.2)** | **19.7 (15 , 25)** | **3480 (2692 , 4215)** | **16.1 (14.4 , 17.8)** | **17 (13.1 , 20.5)** | **-13.6 (-35.3 , 13)** |
| **Pakistan** | **17144 (14634 , 19601)** | **23.3 (21 , 25.7)** | **31.1 (26.4 , 35.8)** | **29292 (22444 , 37715)** | **16.3 (13.4 , 19.3)** | **27.9 (21.6 , 35.3)** | **-10.4 (-32 , 18.4)** |
| **Southern Sub-Saharan Africa** | **9007 (7728 , 10780)** | **24.6 (22.4 , 27.3)** | **33.5 (28.7 , 40.1)** | **12463 (11186 , 13781)** | **16.8 (15.5 , 18.1)** | **22.8 (20.6 , 25.2)** | **-32 (-41.3 , -22.4)** |
| **Botswana** | **150 (115 , 188)** | **20.3 (17.8 , 23)** | **27.3 (21.3 , 34.2)** | **356 (259 , 456)** | **17.2 (15.1 , 19.2)** | **27.8 (20.9 , 35)** | **1.8 (-24.1 , 34.1)** |
| **Lesotho** | **204 (160 , 258)** | **19.1 (16.5 , 22.4)** | **21.3 (16.7 , 26.6)** | **376 (285 , 495)** | **17 (14.7 , 19.6)** | **30.6 (23.4 , 40)** | **44 (6 , 96.3)** |
| **Namibia** | **95 (75 , 116)** | **15.5 (13.1 , 18.1)** | **13.9 (11 , 16.8)** | **177 (139 , 224)** | **11.1 (9.5 , 12.8)** | **13.8 (11 , 17.3)** | **-1.1 (-21.9 , 26.5)** |
| **South Africa** | **7310 (6161 , 8974)** | **26.3 (23.9 , 29.4)** | **35.5 (30 , 43.9)** | **9753 (8678 , 10977)** | **17.8 (16.4 , 19.1)** | **22.3 (19.9 , 25)** | **-37.3 (-46.9 , -27.1)** |
| **Eswatini** | **50 (38 , 65)** | **12.4 (10.2 , 15.1)** | **18.7 (14.3 , 23.7)** | **82 (58 , 111)** | **8.5 (6.8 , 10.3)** | **15.7 (11.1 , 21)** | **-16.4 (-37.8 , 14.9)** |
| **Zimbabwe** | **1198 (1020 , 1392)** | **20.1 (17.7 , 22.6)** | **31.3 (26.8 , 36.2)** | **1719 (1363 , 2068)** | **14 (12 , 15.9)** | **26.8 (21.5 , 32.3)** | **-14.4 (-31 , 4)** |
| **Western Sub-Saharan Africa** | **5709 (4769 , 6712)** | **7 (6.2 , 7.8)** | **6.8 (5.8 , 8)** | **11595 (9622 , 13931)** | **5.9 (5.2 , 6.7)** | **6.7 (5.7 , 8)** | **-1.6 (-15.9 , 15.5)** |
| **Benin** | **217 (177 , 260)** | **10.2 (8.8 , 11.7)** | **11.4 (9.2 , 13.6)** | **378 (287 , 492)** | **7.2 (6.2 , 8.2)** | **8.5 (6.5 , 11)** | **-24.7 (-41.7 , -3)** |
| **Burkina Faso** | **268 (207 , 348)** | **6.1 (5 , 7.5)** | **6.3 (4.8 , 8)** | **505 (385 , 646)** | **5 (4.2 , 5.8)** | **5.8 (4.5 , 7.4)** | **-8 (-26.9 , 16.6)** |
| **Cameroon** | **441 (346 , 548)** | **9.3 (8.1 , 10.5)** | **10.2 (8.1 , 12.7)** | **1152 (845 , 1549)** | **8.1 (6.9 , 9.1)** | **10.3 (7.6 , 13.6)** | **0.6 (-26.9 , 36.3)** |
| **Cabo Verde** | **25 (21 , 28)** | **9.6 (8.4 , 11)** | **10.7 (9.2 , 12.4)** | **59 (48 , 70)** | **8.2 (7.1 , 9.5)** | **14.5 (11.7 , 17.2)** | **35.8 (11.6 , 64.1)** |
| **Chad** | **244 (186 , 312)** | **9.3 (7.4 , 11.5)** | **8.9 (6.9 , 11.5)** | **524 (390 , 706)** | **8.4 (6.9 , 10.1)** | **10.4 (7.8 , 13.8)** | **15.9 (-10.5 , 52)** |
| **Côte d'Ivoire** | **473 (361 , 597)** | **10.5 (9 , 12.2)** | **12.4 (9.5 , 15.4)** | **1298 (969 , 1680)** | **11.2 (9.9 , 12.5)** | **13.1 (10 , 16.7)** | **6.2 (-19 , 37.3)** |
| **Gambia** | **39 (27 , 54)** | **12.2 (9 , 15.2)** | **11.2 (8 , 15.3)** | **98 (67 , 135)** | **9.2 (6.7 , 11.7)** | **10.6 (7.4 , 14.3)** | **-5.4 (-31.1 , 29.3)** |
| **Ghana** | **403 (315 , 501)** | **5.5 (4.7 , 6.3)** | **7.2 (5.7 , 8.8)** | **879 (689 , 1095)** | **4.8 (4.1 , 5.5)** | **6.1 (4.8 , 7.5)** | **-15.3 (-34.5 , 9.8)** |
| **Guinea** | **425 (324 , 538)** | **9.2 (7.4 , 11.2)** | **13.2 (10.1 , 16.6)** | **874 (631 , 1153)** | **10.4 (8.3 , 12.5)** | **16.5 (12.1 , 21.5)** | **25.5 (-5.3 , 65.1)** |
| **Guinea-Bissau** | **40 (27 , 54)** | **6.9 (5.6 , 8.5)** | **10 (6.8 , 13.3)** | **51 (37 , 69)** | **5.3 (4.4 , 6.3)** | **7.4 (5.4 , 9.9)** | **-25.2 (-45.1 , 2.2)** |
| **Liberia** | **108 (84 , 135)** | **9.3 (7.9 , 10.8)** | **9.8 (7.7 , 12.2)** | **141 (101 , 192)** | **6.9 (5.9 , 7.9)** | **7.6 (5.5 , 10.2)** | **-22.5 (-44 , 4.2)** |
| **Mali** | **281 (218 , 352)** | **6.1 (4.9 , 7.4)** | **7.2 (5.6 , 8.8)** | **755 (543 , 993)** | **8.1 (6.5 , 9.6)** | **9.6 (7 , 12.6)** | **34.7 (1.7 , 75)** |
| **Mauritania** | **110 (88 , 133)** | **9.6 (8.2 , 11)** | **11 (8.9 , 13.2)** | **185 (130 , 244)** | **8.9 (7.6 , 10.4)** | **9.2 (6.7 , 12)** | **-16.1 (-41.2 , 17.8)** |
| **Niger** | **94 (68 , 126)** | **3.8 (2.9 , 4.8)** | **3.7 (2.7 , 4.8)** | **319 (218 , 443)** | **4.6 (3.7 , 5.6)** | **4.8 (3.3 , 6.5)** | **29.8 (0.1 , 67.2)** |
| **Nigeria** | **1742 (1253 , 2263)** | **5.1 (4 , 6.3)** | **4.2 (3.1 , 5.4)** | **2869 (2141 , 3882)** | **3.5 (2.7 , 4.5)** | **3.8 (2.8 , 5)** | **-10.8 (-37 , 29.9)** |
| **Sao Tome and Principe** | **5 (4 , 6)** | **6.1 (5.1 , 7.1)** | **7 (5.5 , 8.7)** | **10 (8 , 13)** | **7.2 (6.2 , 8.2)** | **10.9 (8.8 , 13.2)** | **54.6 (19.1 , 102.9)** |
| **Senegal** | **355 (273 , 440)** | **10.6 (9 , 12.5)** | **11.2 (8.6 , 13.8)** | **694 (527 , 895)** | **8.4 (7.4 , 9.6)** | **9.7 (7.4 , 12.4)** | **-13.5 (-33.9 , 16.7)** |
| **Sierra Leone** | **271 (212 , 339)** | **14.5 (12.5 , 16.5)** | **14.5 (11.4 , 18)** | **389 (283 , 522)** | **10.2 (8.9 , 11.8)** | **11.5 (8.5 , 15.2)** | **-21.1 (-40.8 , 5.4)** |
| **Togo** | **170 (136 , 209)** | **12.6 (10.5 , 14.8)** | **14.9 (12 , 18.2)** | **413 (308 , 547)** | **10.8 (9.3 , 12.3)** | **12.3 (9.4 , 15.9)** | **-17.7 (-37.4 , 10.1)** |
| **Eastern Sub-Saharan Africa** | **8022 (6589 , 9561)** | **8.3 (7 , 9.9)** | **11.4 (9.4 , 13.4)** | **14479 (11831 , 17806)** | **7.6 (6.7 , 8.7)** | **9.6 (8 , 11.7)** | **-15.7 (-26.3 , -3.8)** |
| **Burundi** | **393 (298 , 494)** | **11.3 (9.4 , 13.4)** | **17.1 (13.2 , 21.4)** | **409 (289 , 559)** | **7.8 (6.5 , 9.1)** | **9.3 (6.6 , 12.4)** | **-45.9 (-60.6 , -25.3)** |
| **Comoros** | **29 (17 , 38)** | **11.2 (9.4 , 13)** | **13.8 (8.5 , 17.9)** | **48 (37 , 63)** | **8.9 (7.5 , 10.3)** | **10.5 (8.1 , 13.5)** | **-23.6 (-44.6 , 23)** |
| **Djibouti** | **21 (15 , 30)** | **11.8 (9.7 , 14.2)** | **16.4 (11.7 , 22.6)** | **104 (71 , 156)** | **13.8 (11.3 , 16.9)** | **19.2 (13.7 , 27.8)** | **17 (-12.3 , 59.4)** |
| **Eritrea** | **87 (52 , 116)** | **6.7 (4.4 , 8.8)** | **8 (4.7 , 10.5)** | **185 (130 , 245)** | **5.1 (3.9 , 6.2)** | **6.6 (4.7 , 8.5)** | **-17.9 (-40.4 , 23)** |
| **Ethiopia** | **1106 (736 , 1608)** | **3.9 (2.7 , 5.2)** | **5.7 (3.7 , 8.3)** | **1252 (882 , 1715)** | **3.2 (2.4 , 4)** | **3.3 (2.4 , 4.6)** | **-41.4 (-60.5 , -17.4)** |
| **Kenya** | **650 (511 , 805)** | **9.4 (7.8 , 11)** | **8.4 (6.7 , 10.4)** | **1888 (1527 , 2307)** | **8.1 (6.8 , 9.3)** | **9.2 (7.5 , 11.1)** | **9.1 (-9.8 , 33.5)** |
| **Madagascar** | **559 (422 , 706)** | **9.3 (7.7 , 11.3)** | **11.4 (8.7 , 14.2)** | **643 (465 , 886)** | **5.8 (4.9 , 7)** | **6.2 (4.5 , 8.4)** | **-45.8 (-59.8 , -28.6)** |
| **Malawi** | **475 (381 , 597)** | **7.9 (6.4 , 9.8)** | **13.4 (10.9 , 16.5)** | **943 (714 , 1240)** | **9.3 (7.7 , 11.2)** | **13.8 (10.6 , 17.5)** | **2.7 (-19.8 , 28.3)** |
| **Mozambique** | **490 (382 , 615)** | **8.3 (6.9 , 9.8)** | **8.8 (7 , 10.9)** | **1057 (809 , 1373)** | **7.8 (6.6 , 9.2)** | **10.4 (8.1 , 13.3)** | **17.5 (-10.8 , 57.4)** |
| **Rwanda** | **602 (458 , 758)** | **12.9 (10.5 , 15.6)** | **21.9 (17 , 27.4)** | **994 (753 , 1298)** | **13.9 (11.7 , 16.3)** | **18.7 (14.4 , 23.8)** | **-14.8 (-34.7 , 17.5)** |
| **Somalia** | **294 (198 , 407)** | **8.6 (6.6 , 10.7)** | **12.2 (8.3 , 16.5)** | **566 (355 , 804)** | **7.1 (5.5 , 8.9)** | **8.9 (5.6 , 12.5)** | **-27.3 (-46.6 , 1.4)** |
| **South Sudan** | **309 (218 , 417)** | **10.3 (8.3 , 12.5)** | **13.6 (9.7 , 18)** | **356 (247 , 497)** | **8.6 (6.9 , 10.2)** | **10.4 (7.3 , 14.1)** | **-23.8 (-43.7 , 2)** |
| **United Republic of Tanzania** | **1963 (1499 , 2600)** | **13.7 (11.4 , 16.3)** | **18.7 (14.4 , 24.5)** | **3739 (2753 , 5134)** | **11.9 (10 , 14.2)** | **16.1 (12.1 , 21.7)** | **-13.8 (-30.9 , 4.5)** |
| **Uganda** | **600 (462 , 759)** | **7.4 (6.2 , 8.8)** | **9.7 (7.5 , 12.2)** | **1396 (1086 , 1778)** | **6.5 (5.4 , 7.7)** | **10.6 (8.4 , 13.3)** | **9.5 (-13.1 , 37)** |
| **Zambia** | **438 (347 , 539)** | **9.5 (7.8 , 11.3)** | **17 (13.6 , 20.7)** | **887 (673 , 1120)** | **9.1 (7.6 , 10.4)** | **15 (11.9 , 18.6)** | **-11.5 (-30.2 , 13.7)** |
| **Central Sub-Saharan Africa** | **3031 (2206 , 4701)** | **10.8 (8.7 , 14.9)** | **13.9 (10.1 , 21.1)** | **5054 (3608 , 7121)** | **8.7 (7.3 , 11.1)** | **9.9 (7.1 , 13.7)** | **-28.6 (-45.3 , -8.9)** |
| **Angola** | **675 (492 , 879)** | **13.8 (11.6 , 16.2)** | **17.9 (13.1 , 22.9)** | **1567 (1253 , 1979)** | **12.3 (10.9 , 13.6)** | **14.9 (12 , 18.3)** | **-17.1 (-39 , 13)** |
| **Central African Republic** | **182 (128 , 255)** | **10.9 (8.7 , 14)** | **15.3 (11 , 21.3)** | **229 (154 , 345)** | **8.6 (6.6 , 11.1)** | **10.5 (7.2 , 15.3)** | **-31.5 (-47.8 , -13.9)** |
| **Congo** | **174 (126 , 232)** | **10.5 (8.4 , 12.8)** | **16.9 (12.8 , 22.2)** | **321 (252 , 411)** | **9.6 (8.3 , 11)** | **13.5 (11 , 16.7)** | **-20 (-38.4 , 6.2)** |
| **Democratic Republic of the Congo** | **1892 (1238 , 3323)** | **10.1 (7.5 , 15.1)** | **12.6 (8.2 , 21.7)** | **2738 (1703 , 4359)** | **7.4 (5.6 , 10.7)** | **7.9 (4.9 , 12.6)** | **-37 (-52.7 , -16.8)** |
| **Equatorial Guinea** | **26 (16 , 39)** | **10.8 (7.9 , 14.8)** | **12.8 (8.2 , 19.1)** | **52 (36 , 75)** | **8.7 (7.1 , 10.7)** | **11.9 (8.4 , 16.7)** | **-7 (-44.1 , 54.5)** |
| **Gabon** | **84 (60 , 112)** | **10.3 (8.4 , 12.8)** | **14.7 (10.8 , 19.7)** | **147 (109 , 190)** | **10.2 (8.8 , 11.9)** | **14.4 (10.9 , 18.4)** | **-2.3 (-25.7 , 27)** |
