# Supplementary material for: Global, regional, and national burden of cancers attributable to tobacco smoking in 204 countries and territories, 1990–2019
Source: Cancer Med. 2022 May 27;11(13):2662–78. doi: 10.1002/cam4.4647 (PMC9249976; doi:10.1002/cam4.4647)
Supplement: Supplementary file 15 — Table S4 [file CAM4-11-2662-s011.doc]

| **Table S4: Number, proportion and age-standardised rates of disability-adjusted-life-years (DALYs) due to cancers attributable to smoking (per 100,000) in 1990 and 2019, by location (Generated from data available from http://ghdx.healthdata.org/gbd-results-tool)** | | | | | | | |
| --- | --- | --- | --- | --- | --- | --- | --- |
|  | **1990** | | | **2019** | | | **% change in ASRs per 100,000**  **1990-2019** |
|  | **No**  **(95% UI)** | **PAF**  **(95% UI)** | **ASRs per 100,000 (95% UI)** | **No**  **(95% UI)** | **PAF**  **(95% UI)** | **ASRs per 100,000 (95% UI)** |
| **Global** | **38800909 (36280867 , 41341247)** | **23.6 (22.3 , 24.8)** | **948.9 (888.1 , 1009.1)** | **56446919 (51304882 , 61747452)** | **22.5 (21.2 , 23.8)** | **677.3 (616.4 , 740.3)** | **-28.6 (-35.1 , -21.5)** |
| **High-income North America** | **4860298 (4658056 , 5058581)** | **34.7 (33.4 , 36.2)** | **1455.5 (1397.3 , 1513.7)** | **5347818 (5042527 , 5634478)** | **28.8 (27.5 , 30.1)** | **874.8 (826 , 919.5)** | **-39.9 (-42 , -37.5)** |
| **Canada** | **452167 (432298 , 472605)** | **35.2 (33.8 , 36.7)** | **1413.5 (1353.5 , 1476.6)** | **518606 (481395 , 553924)** | **26.7 (25.2 , 28.3)** | **773.3 (719.4 , 825.2)** | **-45.3 (-48.3 , -42.3)** |
| **Greenland** | **1154 (1013 , 1322)** | **39 (36.2 , 41.9)** | **3002.2 (2681.4 , 3381.4)** | **1658 (1339 , 2009)** | **40.9 (38.1 , 43.8)** | **2224 (1804.5 , 2678.8)** | **-25.9 (-40.8 , -10.2)** |
| **United States of America** | **4406866 (4218114 , 4588217)** | **34.6 (33.3 , 36.2)** | **1460.5 (1399.6 , 1519.2)** | **4827469 (4559641 , 5085976)** | **29 (27.6 , 30.4)** | **886.8 (838.9 , 932.9)** | **-39.3 (-41.4 , -36.8)** |
| **Australasia** | **246851 (234150 , 259146)** | **27 (25.6 , 28.3)** | **1058.3 (1004 , 1111.8)** | **244642 (226166 , 262136)** | **18.5 (17.4 , 19.6)** | **525.2 (487.9 , 563.9)** | **-50.4 (-53 , -48)** |
| **Australia** | **203426 (193488 , 214094)** | **27.1 (25.7 , 28.4)** | **1045.8 (994.5 , 1100.2)** | **201329 (185632 , 216693)** | **18.3 (17.2 , 19.5)** | **515.5 (477.1 , 555.5)** | **-50.7 (-53.6 , -48)** |
| **New Zealand** | **43425 (40762 , 46366)** | **26.6 (25 , 28.4)** | **1120.3 (1051 , 1197.5)** | **43313 (40111 , 46721)** | **19.4 (18.1 , 20.7)** | **576.8 (533.9 , 620.5)** | **-48.5 (-51.2 , -45.5)** |
| **High-income Asia Pacific** | **2078791 (1937127 , 2199522)** | **28.4 (26.6 , 30)** | **1006.4 (937.7 , 1065)** | **2429548 (2208075 , 2606176)** | **24.6 (23.2 , 26.1)** | **585.9 (538.4 , 626.8)** | **-41.8 (-44.7 , -39.1)** |
| **Brunei Darussalam** | **1400 (1188 , 1612)** | **19.8 (18.1 , 21.7)** | **1410.2 (1192.4 , 1619.4)** | **2596 (2220 , 3043)** | **16.3 (14.4 , 18.4)** | **862.4 (746.8 , 1003.3)** | **-38.8 (-49.8 , -25.5)** |
| **Japan** | **1685287 (1565890 , 1789064)** | **29.4 (27.5 , 31.1)** | **973.6 (904.7 , 1033.7)** | **1817639 (1639022 , 1952849)** | **24.5 (23.1 , 25.9)** | **577.6 (529.9 , 616.8)** | **-40.7 (-43.5 , -37.8)** |
| **Singapore** | **22895 (21207 , 24640)** | **24.2 (22.5 , 26.1)** | **997.1 (926 , 1074.8)** | **28861 (26099 , 31568)** | **17.9 (16.3 , 19.3)** | **361.6 (326.2 , 395.1)** | **-63.7 (-67 , -60.6)** |
| **Republic of Korea** | **369208 (343111 , 398458)** | **24.7 (22.9 , 26.5)** | **1112.9 (1038.3 , 1201.3)** | **580452 (523630 , 641478)** | **25.6 (23.7 , 27.6)** | **639.6 (575.5 , 707.3)** | **-42.5 (-47.9 , -35.6)** |
| **Western Europe** | **6849815 (6572709 , 7108951)** | **31.1 (29.8 , 32.3)** | **1248.4 (1198.6 , 1295.8)** | **6785694 (6407173 , 7155155)** | **27.2 (26 , 28.5)** | **834.1 (791.3 , 876.4)** | **-33.2 (-35.2 , -31.1)** |
| **Andorra** | **790 (611 , 1063)** | **30.8 (28.1 , 33.4)** | **1369.7 (1064.3 , 1838.9)** | **1358 (1014 , 1748)** | **27.1 (24.9 , 29.4)** | **974.9 (728.2 , 1259.8)** | **-28.8 (-51 , -1)** |
| **Austria** | **111620 (105507 , 118000)** | **24.4 (23.1 , 25.7)** | **1016.5 (962.4 , 1073.9)** | **122002 (114286 , 129916)** | **27.4 (26 , 28.7)** | **760.6 (714.6 , 808.1)** | **-25.2 (-29.1 , -21.2)** |
| **Belgium** | **222168 (212317 , 232532)** | **33.8 (32.4 , 35.2)** | **1510.3 (1446.4 , 1578)** | **196560 (184167 , 209630)** | **29.9 (28.3 , 31.4)** | **954.8 (896.8 , 1014.7)** | **-36.8 (-40.2 , -33.3)** |
| **Cyprus** | **5321 (4650 , 5889)** | **23.6 (21.7 , 25.7)** | **639.6 (560.7 , 708.5)** | **13281 (11704 , 14964)** | **27 (25.4 , 28.5)** | **678.5 (599 , 762.8)** | **6.1 (-9.2 , 25.8)** |
| **Denmark** | **127554 (121126 , 133918)** | **37.7 (35.9 , 39.4)** | **1695.1 (1613 , 1775.8)** | **110249 (101651 , 119513)** | **30.3 (28.6 , 32.1)** | **995.1 (923.4 , 1075.2)** | **-41.3 (-44.9 , -36.9)** |
| **Finland** | **60560 (57257 , 64005)** | **25.7 (24.3 , 27.2)** | **871.9 (824.8 , 922.1)** | **57736 (53442 , 62339)** | **20.7 (19.5 , 21.9)** | **506.3 (467.2 , 546.8)** | **-41.9 (-46.3 , -37.5)** |
| **France** | **980889 (928603 , 1032441)** | **29.7 (28.2 , 31.2)** | **1301.8 (1235.1 , 1368.9)** | **1042022 (971030 , 1115114)** | **27.2 (25.8 , 28.8)** | **898.1 (839.4 , 962.1)** | **-31 (-34.8 , -26.7)** |
| **Germany** | **1331349 (1257094 , 1403729)** | **28.9 (27.3 , 30.5)** | **1116.6 (1055.5 , 1175.3)** | **1457707 (1356846 , 1553357)** | **27.4 (26 , 29)** | **867.5 (809.4 , 924.3)** | **-22.3 (-26.8 , -17.5)** |
| **Greece** | **187714 (179540 , 196271)** | **35.1 (33.7 , 36.6)** | **1242.5 (1190 , 1298.3)** | **232682 (217136 , 248362)** | **35.3 (33.8 , 36.9)** | **1139.1 (1065.2 , 1213.4)** | **-8.3 (-13.3 , -2.8)** |
| **Iceland** | **2884 (2682 , 3090)** | **28.8 (27.3 , 30.4)** | **1052.7 (981.4 , 1128.1)** | **3423 (3084 , 3817)** | **25.2 (23.8 , 26.7)** | **650.5 (586 , 724.7)** | **-38.2 (-45 , -31)** |
| **Ireland** | **52739 (50000 , 55714)** | **31.5 (30.1 , 33.1)** | **1298.9 (1231.7 , 1371.6)** | **54677 (50668 , 59255)** | **25.3 (23.9 , 26.7)** | **741.8 (687.2 , 803.4)** | **-42.9 (-46.7 , -38.9)** |
| **Israel** | **39173 (36519 , 41870)** | **23.7 (22.2 , 25.3)** | **824.2 (769.6 , 880.4)** | **63893 (58876 , 68877)** | **21 (19.7 , 22.3)** | **574.7 (530.7 , 619)** | **-30.3 (-35.1 , -25)** |
| **Italy** | **1087860 (1036719 , 1136977)** | **31.3 (29.9 , 32.8)** | **1260.4 (1201.1 , 1315.9)** | **901950 (835827 , 958235)** | **24.9 (23.6 , 26.3)** | **709.5 (662.6 , 751.7)** | **-43.7 (-46.4 , -41)** |
| **Luxembourg** | **7439 (6869 , 8100)** | **31 (28.9 , 33.4)** | **1403.4 (1293.3 , 1531.4)** | **7296 (6343 , 8397)** | **26.9 (24.7 , 29)** | **762.1 (660.9 , 876.4)** | **-45.7 (-52.9 , -37.8)** |
| **Malta** | **4030 (3762 , 4292)** | **27.5 (26 , 29)** | **932 (870.5 , 993.6)** | **5106 (4559 , 5694)** | **24.7 (23.3 , 26.1)** | **598 (536.1 , 667.6)** | **-35.8 (-42.8 , -27.7)** |
| **Monaco** | **864 (694 , 1051)** | **30.6 (28.2 , 33.2)** | **1444.3 (1164.3 , 1761.3)** | **1304 (1046 , 1577)** | **30.1 (27.6 , 32.6)** | **1566.5 (1243.1 , 1920.2)** | **8.5 (-19.1 , 47.2)** |
| **Netherlands** | **306660 (292487 , 320844)** | **37.5 (35.9 , 39.1)** | **1587.4 (1515.1 , 1658.3)** | **329282 (305346 , 353229)** | **29.4 (27.8 , 31)** | **1003.6 (934.4 , 1076.5)** | **-36.8 (-40.3 , -32.9)** |
| **Norway** | **52984 (49860 , 56444)** | **23.9 (22.5 , 25.5)** | **856.9 (805.7 , 913)** | **46985 (43249 , 50583)** | **18.8 (17.5 , 20.1)** | **518.9 (479.1 , 557.6)** | **-39.5 (-43.2 , -35.7)** |
| **Portugal** | **112864 (105629 , 120629)** | **21.9 (20.4 , 23.4)** | **826.3 (772.3 , 882.8)** | **135790 (126272 , 146033)** | **21.6 (20.3 , 22.9)** | **682.3 (633.4 , 734.1)** | **-17.4 (-23.6 , -11.3)** |
| **San Marino** | **372 (313 , 434)** | **27.9 (25.5 , 30.3)** | **1141.8 (959.3 , 1336.5)** | **504 (319 , 740)** | **22.6 (20.6 , 24.6)** | **867.4 (539.4 , 1290.9)** | **-24 (-52.2 , 16.6)** |
| **Spain** | **643610 (613008 , 672173)** | **32.9 (31.6 , 34.4)** | **1236.6 (1177.6 , 1289.2)** | **748858 (696546 , 799151)** | **30.3 (28.8 , 32)** | **902.1 (840.5 , 964.2)** | **-27 (-31.2 , -22.7)** |
| **Sweden** | **101169 (94257 , 107973)** | **22.4 (21.1 , 23.8)** | **732.9 (684.6 , 779)** | **105473 (96938 , 113278)** | **21.3 (19.9 , 22.6)** | **529.2 (488.7 , 566.9)** | **-27.8 (-31.7 , -23.6)** |
| **Switzerland** | **106358 (101020 , 111831)** | **31 (29.5 , 32.6)** | **1104.1 (1048 , 1160.8)** | **100580 (92554 , 108144)** | **25.4 (23.9 , 26.9)** | **626.4 (578.8 , 673.6)** | **-43.3 (-46.7 , -39.4)** |
| **United Kingdom** | **1297158 (1239727 , 1355962)** | **35.3 (33.8 , 36.8)** | **1497.9 (1432.4 , 1565.4)** | **1041060 (979584 , 1105873)** | **27.8 (26.4 , 29.3)** | **878.2 (830.1 , 931.7)** | **-41.4 (-43.5 , -38.7)** |
| **Southern Latin America** | **548531 (517539 , 577315)** | **26.2 (24.8 , 27.5)** | **1165.7 (1099.4 , 1227.2)** | **631244 (586495 , 676360)** | **21.9 (20.6 , 23.3)** | **772.3 (717.8 , 827.9)** | **-33.7 (-37.2 , -30.5)** |
| **Argentina** | **406889 (384201 , 429214)** | **27.8 (26.3 , 29.3)** | **1239.2 (1170.7 , 1307.2)** | **460697 (426505 , 495464)** | **23.3 (21.8 , 24.8)** | **875.2 (810 , 941.7)** | **-29.4 (-33.9 , -25.2)** |
| **Chile** | **83481 (75556 , 91466)** | **19.2 (17.4 , 21)** | **798.6 (724.9 , 875.1)** | **117128 (105973 , 128514)** | **17 (15.6 , 18.4)** | **487 (440.6 , 534.4)** | **-39 (-44 , -33.6)** |
| **Uruguay** | **58138 (54831 , 61381)** | **29.4 (27.9 , 31)** | **1537.3 (1449.1 , 1622.8)** | **53388 (49490 , 57571)** | **25.3 (23.8 , 26.8)** | **1097.2 (1018.4 , 1184.1)** | **-28.6 (-33.7 , -23.8)** |
| **Eastern Europe** | **3553196 (3402484 , 3712173)** | **29.1 (27.8 , 30.3)** | **1231.7 (1178.1 , 1286.1)** | **3007670 (2624733 , 3415500)** | **26.8 (24.8 , 28.9)** | **894.4 (780 , 1015.5)** | **-27.4 (-36.2 , -17.8)** |
| **Belarus** | **173911 (163450 , 184046)** | **31.1 (29.4 , 32.8)** | **1317.9 (1238.5 , 1395.9)** | **142982 (110210 , 184943)** | **28.5 (26.7 , 30.2)** | **915.4 (705.6 , 1193.9)** | **-30.5 (-46.4 , -10.3)** |
| **Estonia** | **28702 (27097 , 30389)** | **31.1 (29.4 , 32.8)** | **1397.4 (1319.1 , 1480.1)** | **21809 (16998 , 27564)** | **27.1 (25.6 , 28.6)** | **924.9 (717.3 , 1171.6)** | **-33.8 (-48.6 , -16.1)** |
| **Latvia** | **46701 (43990 , 49514)** | **30.9 (29.2 , 32.6)** | **1306.3 (1231.7 , 1383.9)** | **31738 (25712 , 39062)** | **25.9 (22.8 , 28.9)** | **905.9 (732.2 , 1117.6)** | **-30.7 (-43.8 , -13.8)** |
| **Lithuania** | **53687 (50503 , 57021)** | **28.3 (26.8 , 30)** | **1185.2 (1114.3 , 1258)** | **42565 (34383 , 52235)** | **24.4 (22.8 , 26)** | **836.4 (670.4 , 1031.1)** | **-29.4 (-43.1 , -13.2)** |
| **Republic of Moldova** | **50861 (46536 , 55148)** | **26 (23.9 , 28.1)** | **1077.8 (987.7 , 1168.1)** | **44384 (38116 , 51366)** | **26.3 (24.6 , 28)** | **769.3 (659.9 , 887.9)** | **-28.6 (-39 , -17.5)** |
| **Russian Federation** | **2186117 (2076203 , 2292838)** | **28.3 (27 , 29.7)** | **1159.3 (1101 , 1215.3)** | **2011830 (1682176 , 2370760)** | **26.9 (24.2 , 29.5)** | **866.7 (725 , 1021.8)** | **-25.2 (-37.1 , -11.7)** |
| **Ukraine** | **1013218 (957616 , 1066138)** | **30.7 (29 , 32.2)** | **1410 (1330.4 , 1485)** | **712362 (577546 , 860774)** | **26.4 (23.6 , 29.4)** | **990.4 (800 , 1199.4)** | **-29.8 (-43.3 , -14.2)** |
| **Central Europe** | **2232198 (2145847 , 2321222)** | **33.1 (31.9 , 34.3)** | **1484 (1425.4 , 1542.1)** | **2541463 (2230735 , 2914210)** | **32.4 (31.1 , 33.8)** | **1261.1 (1103.2 , 1447.4)** | **-15 (-25.8 , -3.4)** |
| **Albania** | **19077 (17680 , 20728)** | **24.3 (22.3 , 26)** | **888.1 (822.4 , 962.8)** | **31835 (23230 , 42677)** | **28.5 (26.6 , 30.4)** | **739.9 (539.6 , 993.1)** | **-16.7 (-38.8 , 11.8)** |
| **Bosnia and Herzegovina** | **54223 (50798 , 57529)** | **32.7 (31 , 34.5)** | **1194.5 (1121.2 , 1267.9)** | **78395 (60487 , 100096)** | **35.6 (33.9 , 37.4)** | **1316.9 (1017.1 , 1678.5)** | **10.3 (-15 , 40.7)** |
| **Bulgaria** | **158962 (147611 , 170466)** | **33.7 (31.9 , 35.5)** | **1263.4 (1171.3 , 1353.7)** | **173171 (136473 , 219488)** | **32.8 (31.1 , 34.4)** | **1383.1 (1082.3 , 1756.1)** | **9.5 (-15.2 , 39)** |
| **Croatia** | **113699 (105323 , 123142)** | **37.9 (36 , 39.8)** | **1716.2 (1591.3 , 1856.3)** | **92428 (73181 , 117987)** | **31.3 (29.6 , 33.1)** | **1143.3 (899.9 , 1457.1)** | **-33.4 (-48.3 , -14.2)** |
| **Czechia** | **226533 (213631 , 239643)** | **32.5 (30.7 , 34.2)** | **1683.3 (1586.5 , 1781.4)** | **187540 (152394 , 229940)** | **28.8 (27.4 , 30.4)** | **933.2 (760.3 , 1149.2)** | **-44.6 (-55.3 , -31.8)** |
| **Hungary** | **268420 (255461 , 281730)** | **34.1 (32.6 , 35.6)** | **1876.9 (1786.6 , 1967.4)** | **276742 (225289 , 337327)** | **35.7 (34.2 , 37.3)** | **1588.6 (1290.6 , 1942.8)** | **-15.4 (-30.8 , 2.7)** |
| **Montenegro** | **9812 (8646 , 10931)** | **37.1 (34.5 , 39.6)** | **1505.2 (1326.5 , 1676)** | **16819 (13969 , 20364)** | **41.9 (39.7 , 43.9)** | **1708 (1418.4 , 2070.1)** | **13.5 (-8.1 , 39.1)** |
| **North Macedonia** | **24709 (22419 , 26967)** | **29.7 (27.4 , 32)** | **1228.3 (1114.2 , 1340.5)** | **46716 (36161 , 59899)** | **34.2 (32.3 , 36)** | **1401.2 (1087 , 1798.3)** | **14.1 (-11.4 , 46.4)** |
| **Poland** | **760591 (730230 , 792230)** | **34.8 (33.4 , 36.2)** | **1727.9 (1657.8 , 1798.9)** | **903649 (744327 , 1085591)** | **33.5 (31.5 , 35.6)** | **1336.3 (1097.6 , 1607.2)** | **-22.7 (-36 , -7.9)** |
| **Romania** | **302709 (283707 , 320564)** | **29.2 (27.5 , 30.9)** | **1042.6 (976.4 , 1105.4)** | **380752 (309083 , 464951)** | **29.9 (28.5 , 31.5)** | **1153 (928.3 , 1415.3)** | **10.6 (-9.9 , 37.1)** |
| **Serbia** | **171106 (146236 , 185264)** | **32.5 (30.4 , 34.5)** | **1388.4 (1187.3 , 1499.5)** | **231344 (181450 , 292007)** | **35 (33.3 , 36.9)** | **1538.1 (1195.8 , 1946.4)** | **10.8 (-14.3 , 43.4)** |
| **Slovakia** | **90648 (84801 , 96999)** | **32.6 (30.8 , 34.5)** | **1533 (1434.3 , 1636.9)** | **85220 (65859 , 108684)** | **26.1 (24.4 , 27.9)** | **937.3 (721.8 , 1195.7)** | **-38.9 (-52.4 , -21.5)** |
| **Slovenia** | **31711 (23906 , 41837)** | **30.8 (29.3 , 32.4)** | **1290.3 (973.4 , 1703.2)** | **36854 (28496 , 47914)** | **28.6 (27.1 , 30.3)** | **943.7 (727 , 1228.5)** | **-26.9 (-51.2 , 5.5)** |
| **Central Asia** | **503246 (476938 , 528041)** | **23.3 (22.2 , 24.4)** | **1001.6 (950.8 , 1050)** | **495771 (441353 , 556311)** | **18.4 (17.2 , 19.6)** | **611.1 (545.9 , 682.8)** | **-39 (-45 , -31.8)** |
| **Armenia** | **38249 (36255 , 40287)** | **29.4 (28 , 30.7)** | **1264.6 (1200.1 , 1330.5)** | **39701 (32827 , 47120)** | **27.3 (26 , 28.7)** | **933 (771.8 , 1102.5)** | **-26.2 (-38.7 , -11.7)** |
| **Azerbaijan** | **53134 (47282 , 59057)** | **23 (21 , 24.8)** | **958.1 (855.7 , 1062.9)** | **85901 (65619 , 110136)** | **23.5 (20.2 , 26.9)** | **800.3 (620.3 , 1017.5)** | **-16.5 (-34.6 , 8.1)** |
| **Georgia** | **64598 (57926 , 71864)** | **26.3 (24.5 , 27.9)** | **990.3 (889.7 , 1100.3)** | **56911 (46883 , 67177)** | **26.6 (25.1 , 28.3)** | **1015.9 (836.9 , 1200.1)** | **2.6 (-16.6 , 24.1)** |
| **Kazakhstan** | **193295 (178426 , 208119)** | **28.1 (26.2 , 29.9)** | **1412 (1303.6 , 1516.9)** | **130569 (111113 , 151061)** | **21.7 (20.3 , 23.2)** | **695.2 (592.6 , 801.6)** | **-50.8 (-58.4 , -42.3)** |
| **Kyrgyzstan** | **32294 (29942 , 34637)** | **25.1 (23.7 , 26.4)** | **1024.7 (952.4 , 1098.6)** | **23907 (20542 , 27741)** | **19.1 (17.7 , 20.6)** | **481.6 (416.3 , 554.9)** | **-53 (-59.5 , -46.1)** |
| **Mongolia** | **14801 (11579 , 18411)** | **17.9 (15.3 , 20.5)** | **1392.2 (1098.9 , 1720.4)** | **31053 (22853 , 41728)** | **18.2 (14.7 , 21.3)** | **1266.5 (961.7 , 1652.2)** | **-9 (-31.8 , 21.4)** |
| **Tajikistan** | **23350 (20834 , 26177)** | **19 (17.3 , 20.9)** | **795.6 (711.5 , 892.4)** | **20386 (15896 , 27126)** | **11.3 (10.2 , 13.1)** | **376.9 (298.8 , 490.9)** | **-52.6 (-62.3 , -38.1)** |
| **Turkmenistan** | **19682 (18456 , 20888)** | **22.7 (21 , 24.2)** | **937.9 (883.5 , 993.8)** | **18220 (14281 , 23126)** | **15 (13.9 , 16)** | **409.3 (322.8 , 517.5)** | **-56.4 (-65.5 , -45.5)** |
| **Uzbekistan** | **63842 (56901 , 71237)** | **14.5 (12.9 , 16.1)** | **528.7 (470.4 , 588.9)** | **89122 (71958 , 106758)** | **11.5 (10.6 , 12.5)** | **360.2 (295.2 , 427.4)** | **-31.9 (-44.1 , -17)** |
| **Central Latin America** | **401729 (362847 , 439065)** | **13.3 (12.1 , 14.6)** | **470.6 (429 , 510.8)** | **566546 (468545 , 680541)** | **9.3 (8.3 , 10.2)** | **239.1 (198.6 , 286.6)** | **-49.2 (-57.1 , -40)** |
| **Colombia** | **100001 (89562 , 111635)** | **14.2 (12.8 , 15.9)** | **550.4 (494.5 , 611)** | **116669 (88281 , 155600)** | **9.4 (8.4 , 10.5)** | **222.2 (168.4 , 295.5)** | **-59.6 (-69.2 , -47)** |
| **Costa Rica** | **9541 (8586 , 10575)** | **14.8 (13.4 , 16.4)** | **543.4 (491 , 601.9)** | **15294 (11642 , 19960)** | **10.5 (9.5 , 11.6)** | **296.8 (226.5 , 385.8)** | **-45.4 (-58.7 , -28.9)** |
| **El Salvador** | **6283 (5427 , 7322)** | **7.6 (6.6 , 8.7)** | **207.8 (180.3 , 240.6)** | **10981 (7952 , 14859)** | **7.5 (6.5 , 8.6)** | **189.5 (137.1 , 255.7)** | **-8.8 (-34.2 , 22.8)** |
| **Guatemala** | **9029 (7252 , 11026)** | **7 (5.7 , 8.4)** | **241.8 (197.7 , 291.5)** | **20197 (14571 , 27021)** | **5.7 (4.7 , 6.8)** | **181.1 (131.1 , 241.7)** | **-25.1 (-45.6 , 1)** |
| **Honduras** | **7629 (6026 , 9215)** | **9.5 (8 , 11.1)** | **357.2 (281.3 , 432.2)** | **24237 (17728 , 31876)** | **11.4 (9.1 , 13.8)** | **395.6 (289 , 519.2)** | **10.8 (-16.2 , 45.7)** |
| **Mexico** | **194467 (172902 , 216808)** | **13.1 (11.6 , 14.7)** | **450 (402.9 , 498.4)** | **238684 (195725 , 288040)** | **8.3 (7.2 , 9.4)** | **204.3 (168.2 , 246)** | **-54.6 (-62.1 , -45.6)** |
| **Nicaragua** | **4272 (3683 , 4971)** | **8.4 (7.2 , 9.7)** | **269.6 (235.6 , 309.2)** | **9132 (7089 , 11278)** | **7.6 (6.7 , 8.7)** | **204.6 (160 , 251.7)** | **-24.1 (-40.6 , -5.8)** |
| **Panama** | **6279 (5676 , 6974)** | **13.3 (12.1 , 14.6)** | **418.9 (378.5 , 464.4)** | **8279 (6147 , 10936)** | **8.3 (7.5 , 9.3)** | **200.9 (149.3 , 264.7)** | **-52.1 (-63.7 , -37.2)** |
| **Venezuela (Bolivarian Republic of)** | **64229 (57872 , 70940)** | **16.6 (15 , 18.3)** | **637.3 (576.8 , 698)** | **123073 (90282 , 164631)** | **13.4 (12 , 15.1)** | **411.8 (302.7 , 547.1)** | **-35.4 (-52.1 , -14.1)** |
| **Andean Latin America** | **54117 (45688 , 63310)** | **6.5 (5.6 , 7.4)** | **262.1 (222.9 , 304.7)** | **90847 (70790 , 113852)** | **5.5 (4.8 , 6.3)** | **162.7 (126.9 , 203.7)** | **-37.9 (-49.6 , -23.7)** |
| **Bolivia (Plurinational State of)** | **14703 (11019 , 18701)** | **8.5 (7.1 , 10)** | **449.6 (339.4 , 565.4)** | **24739 (17269 , 33508)** | **6.8 (5.5 , 8)** | **276.8 (194.4 , 373.3)** | **-38.4 (-53.9 , -17.7)** |
| **Ecuador** | **16339 (14010 , 18653)** | **9 (7.8 , 10.3)** | **306.4 (264.7 , 348.3)** | **32356 (24939 , 42061)** | **7.2 (6.3 , 8.2)** | **214.5 (165.7 , 277.3)** | **-30 (-45.4 , -8.5)** |
| **Peru** | **23074 (18493 , 28636)** | **4.8 (4 , 5.7)** | **191.2 (154.5 , 237)** | **33752 (23436 , 47342)** | **4 (3.4 , 4.7)** | **106.1 (73.7 , 148.8)** | **-44.5 (-61.8 , -20.2)** |
| **Caribbean** | **176448 (164311 , 189071)** | **18 (16.7 , 19.6)** | **672.9 (626.8 , 720.1)** | **309857 (260087 , 365171)** | **18 (16.2 , 19.7)** | **595.5 (500 , 701.6)** | **-11.5 (-24.9 , 3.2)** |
| **Antigua and Barbuda** | **157 (138 , 178)** | **9 (8 , 10.2)** | **310.4 (271.9 , 352)** | **303 (249 , 371)** | **9.3 (8.2 , 10.5)** | **289.6 (238.9 , 351.9)** | **-6.7 (-23.8 , 12.9)** |
| **Barbados** | **906 (793 , 1021)** | **8.7 (7.8 , 9.7)** | **336.7 (294.2 , 378.3)** | **1322 (1055 , 1643)** | **7.3 (6.6 , 8.2)** | **272.7 (217.1 , 339.4)** | **-19 (-34.3 , -1.2)** |
| **Belize** | **290 (253 , 329)** | **10.8 (9.5 , 12.1)** | **314.7 (274.7 , 355.7)** | **1005 (844 , 1182)** | **11.6 (10.4 , 12.9)** | **351.4 (296.4 , 410.1)** | **11.6 (-6.5 , 33.6)** |
| **Bermuda** | **526 (453 , 606)** | **17.5 (15.1 , 19.9)** | **830 (714.9 , 954.2)** | **632 (521 , 771)** | **17.6 (15.9 , 19.3)** | **498.4 (410.5 , 610.1)** | **-39.9 (-51.1 , -25.5)** |
| **Bahamas** | **805 (681 , 938)** | **10.4 (9 , 11.9)** | **505 (427.3 , 586.7)** | **1490 (1169 , 1867)** | **9.4 (8.3 , 10.6)** | **360.1 (283.1 , 450.5)** | **-28.7 (-44.9 , -8.1)** |
| **Cuba** | **105310 (98804 , 111606)** | **28.4 (26.7 , 30.1)** | **1026.9 (962.5 , 1088.1)** | **183981 (147188 , 223852)** | **29.9 (28.2 , 32)** | **982 (786.6 , 1195.7)** | **-4.4 (-22.7 , 17.4)** |
| **Dominica** | **351 (301 , 405)** | **10.8 (9.6 , 11.9)** | **519.7 (442.9 , 606.3)** | **385 (306 , 479)** | **9.6 (8.6 , 10.7)** | **428.6 (340.4 , 534.8)** | **-17.5 (-35.2 , 4)** |
| **Dominican Republic** | **14318 (12075 , 16859)** | **13.4 (11.6 , 15.2)** | **379.5 (322 , 443.7)** | **45402 (33001 , 61262)** | **16 (14.3 , 17.6)** | **487.8 (356.9 , 654.7)** | **28.5 (-8 , 72.9)** |
| **Grenada** | **304 (263 , 348)** | **9.4 (8.3 , 10.7)** | **458.6 (395 , 525.6)** | **477 (413 , 545)** | **10 (8.9 , 11.1)** | **403.8 (350.6 , 461.3)** | **-12 (-25.2 , 2.9)** |
| **Guyana** | **1380 (1144 , 1661)** | **8.7 (7.6 , 10.1)** | **344.1 (285.6 , 410.2)** | **1870 (1404 , 2487)** | **8.2 (7.2 , 9.2)** | **275.3 (208.2 , 364.9)** | **-20 (-40.4 , 6.8)** |
| **Haiti** | **14088 (8492 , 19185)** | **6.8 (5.3 , 8.5)** | **390.8 (239.5 , 527.6)** | **17231 (10895 , 24982)** | **5 (4.1 , 6.2)** | **228.6 (146.5 , 326.5)** | **-41.5 (-57.9 , -19)** |
| **Jamaica** | **8138 (7350 , 9002)** | **16 (14.5 , 17.6)** | **478.4 (432.6 , 530.5)** | **14843 (11584 , 18956)** | **14 (12.7 , 15.5)** | **502.9 (393.1 , 641.3)** | **5.1 (-18.2 , 32.5)** |
| **Puerto Rico** | **17398 (15308 , 19583)** | **16.1 (14.2 , 18.1)** | **480.2 (422.2 , 541.8)** | **19075 (14291 , 24890)** | **13.5 (12 , 15.1)** | **288.1 (214.1 , 378.3)** | **-40 (-54.7 , -20.2)** |
| **Saint Kitts and Nevis** | **126 (107 , 148)** | **6.8 (5.9 , 7.9)** | **359 (306.2 , 424)** | **216 (173 , 265)** | **8.5 (7.5 , 9.5)** | **300.2 (243.9 , 362.9)** | **-16.4 (-32.9 , 4)** |
| **Saint Lucia** | **460 (408 , 519)** | **11.7 (10.5 , 13)** | **527.1 (468.6 , 593.8)** | **854 (703 , 1026)** | **11.7 (10.5 , 12.7)** | **387.8 (319.9 , 465.4)** | **-26.4 (-39.8 , -11.8)** |
| **Saint Vincent and the Grenadines** | **245 (211 , 281)** | **8.1 (7 , 9.1)** | **346.3 (298.6 , 398)** | **458 (385 , 551)** | **9 (7.9 , 10.2)** | **329.9 (277.4 , 396.3)** | **-4.7 (-21.2 , 13.8)** |
| **Suriname** | **1233 (1063 , 1421)** | **14.4 (12.7 , 16.4)** | **458.4 (397 , 525)** | **2935 (2358 , 3594)** | **15.4 (13.8 , 17.1)** | **469.1 (378.8 , 572.4)** | **2.4 (-16.4 , 26.5)** |
| **Trinidad and Tobago** | **4138 (3726 , 4589)** | **13.1 (11.8 , 14.4)** | **483.3 (436.3 , 534.3)** | **5940 (4330 , 7952)** | **12 (10.8 , 13.2)** | **311.8 (228 , 415.7)** | **-35.5 (-52.9 , -14.8)** |
| **United States Virgin Islands** | **396 (319 , 487)** | **11.3 (9.5 , 13.3)** | **438.5 (355.9 , 535.7)** | **943 (753 , 1132)** | **12.7 (11.1 , 14.5)** | **497 (392.5 , 602.5)** | **13.3 (-12.2 , 45.6)** |
| **Tropical Latin America** | **883764 (821618 , 951004)** | **24.2 (22.5 , 26.1)** | **913.7 (851 , 981.2)** | **1255064 (1156179 , 1350816)** | **17.9 (16.8 , 19.1)** | **506.9 (467.2 , 545.4)** | **-44.5 (-47.7 , -41)** |
| **Brazil** | **872083 (810714 , 938025)** | **24.3 (22.6 , 26.2)** | **923.3 (859.9 , 990.5)** | **1225384 (1128038 , 1317838)** | **17.9 (16.8 , 19.1)** | **506.3 (466.1 , 545)** | **-45.2 (-48.3 , -41.6)** |
| **Paraguay** | **11682 (9940 , 13663)** | **18.8 (16.6 , 21.2)** | **517.5 (444.9 , 601.7)** | **29680 (22176 , 39390)** | **18.1 (16.3 , 19.9)** | **532.1 (398.7 , 700)** | **2.8 (-23.5 , 36)** |
| **East Asia** | **10379695 (8543364 , 12291522)** | **22.3 (19.7 , 24.9)** | **1113.9 (925.6 , 1308.9)** | **21001451 (16984703 , 25387046)** | **30 (27.2 , 32.6)** | **972.6 (790.5 , 1171.3)** | **-12.7 (-32.4 , 14.3)** |
| **China** | **10086980 (8272812 , 11986648)** | **22.4 (19.6 , 25)** | **1124.4 (929.5 , 1327)** | **20400908 (16369209 , 24820749)** | **30.2 (27.3 , 32.9)** | **978.2 (790.2 , 1185.3)** | **-13 (-33.3 , 14.6)** |
| **Democratic People's Republic of Korea** | **141502 (106996 , 188008)** | **18 (15.9 , 20.3)** | **781.6 (599.5 , 1021.3)** | **274839 (215980 , 341506)** | **24 (21.4 , 26.7)** | **823.9 (652 , 1021.2)** | **5.4 (-19.8 , 35.1)** |
| **Taiwan (Province of China)** | **151212 (140602 , 160936)** | **24.8 (23.1 , 26.3)** | **881.8 (822.6 , 936.9)** | **325703 (251623 , 429023)** | **24.4 (23 , 25.9)** | **828.8 (642.8 , 1083.4)** | **-6 (-27 , 25)** |
| **Southeast Asia** | **1651602 (1488929 , 1824205)** | **17 (15.3 , 18.8)** | **615.2 (555.6 , 675.8)** | **3469585 (3009633 , 4007278)** | **18.3 (16.7 , 19.9)** | **546.4 (475.7 , 628.9)** | **-11.2 (-24.2 , 4)** |
| **Cambodia** | **37876 (30702 , 46275)** | **16.4 (13.3 , 20)** | **819.4 (670.7 , 988.1)** | **84080 (65431 , 100469)** | **19.4 (17.6 , 21.5)** | **693.8 (545.4 , 821.9)** | **-15.3 (-36.1 , 8.9)** |
| **Indonesia** | **424450 (364695 , 488218)** | **12.5 (10.6 , 15.2)** | **411.2 (355.7 , 470.1)** | **1186528 (930963 , 1492888)** | **17.6 (14.5 , 20.9)** | **528.9 (419.2 , 655.7)** | **28.6 (-0.6 , 62)** |
| **Lao People's Democratic Republic** | **19551 (14578 , 26367)** | **18.2 (14.7 , 21.7)** | **885.5 (668.2 , 1180.1)** | **26946 (20097 , 34356)** | **17.3 (15.5 , 19.1)** | **602.1 (455.9 , 758)** | **-32 (-52 , -8.5)** |
| **Malaysia** | **55086 (49005 , 61272)** | **15.8 (14.2 , 17.1)** | **579.6 (518.5 , 643.5)** | **137587 (106537 , 173990)** | **16.1 (14.8 , 17.5)** | **500.9 (392.4 , 632.4)** | **-13.6 (-33.7 , 10.2)** |
| **Maldives** | **472 (351 , 583)** | **13.9 (11.2 , 16.7)** | **526.6 (404.4 , 643.7)** | **910 (737 , 1095)** | **15.2 (13.9 , 16.5)** | **301.6 (244.2 , 360.7)** | **-42.7 (-57.6 , -20.4)** |
| **Mauritius** | **3411 (3141 , 3694)** | **17.3 (16 , 18.6)** | **448.2 (413.4 , 483.6)** | **5530 (4389 , 6860)** | **14.1 (13.1 , 15.2)** | **305.1 (244.5 , 378.5)** | **-31.9 (-45.8 , -16.4)** |
| **Myanmar** | **217786 (159492 , 302303)** | **18.5 (14.9 , 21.6)** | **896.3 (662.2 , 1229.8)** | **248889 (190493 , 324323)** | **16.2 (14 , 18.9)** | **529.8 (408.1 , 682.9)** | **-40.9 (-56 , -16.4)** |
| **Philippines** | **233402 (195965 , 268068)** | **18 (15.6 , 20.3)** | **736 (623.7 , 841.1)** | **425734 (331924 , 537123)** | **16.6 (14.1 , 19.3)** | **519.8 (410.6 , 650.8)** | **-29.4 (-46 , -6.2)** |
| **Sri Lanka** | **33254 (29074 , 37644)** | **12.8 (11.3 , 14.2)** | **300.7 (263.6 , 339)** | **61404 (44297 , 83884)** | **12.6 (11.4 , 13.9)** | **230.7 (167.6 , 313.9)** | **-23.3 (-44.8 , 4.7)** |
| **Seychelles** | **476 (411 , 535)** | **20.2 (18.1 , 22.4)** | **852 (735.8 , 959.9)** | **914 (789 , 1076)** | **19 (17.2 , 20.9)** | **794.3 (688.1 , 926.8)** | **-6.8 (-20.2 , 10.4)** |
| **Thailand** | **351291 (303205 , 406547)** | **23 (20.7 , 25.3)** | **923.3 (798.3 , 1062.2)** | **562590 (400506 , 756760)** | **19.3 (17 , 21.5)** | **539.4 (384.8 , 721.9)** | **-41.6 (-58.2 , -20.3)** |
| **Timor-Leste** | **1356 (1017 , 1789)** | **12 (8.9 , 16.9)** | **454.8 (347.6 , 598.9)** | **3883 (2599 , 5010)** | **16.2 (13.3 , 19.6)** | **465.1 (313.8 , 593.4)** | **2.3 (-26.8 , 39.5)** |
| **Viet Nam** | **270997 (214032 , 334003)** | **20.6 (18.8 , 22.3)** | **654.7 (517.3 , 802.4)** | **720046 (557659 , 896163)** | **22.6 (20.7 , 24.8)** | **715.2 (559.7 , 881.7)** | **9.2 (-19.7 , 47)** |
| **Oceania** | **18477 (14816 , 23522)** | **15.2 (13.1 , 17.4)** | **566.8 (457 , 717.9)** | **39453 (29635 , 52553)** | **13.1 (11.2 , 15.2)** | **512.5 (394.3 , 674.9)** | **-9.6 (-27.4 , 12.1)** |
| **American Samoa** | **181 (153 , 208)** | **19.5 (17.3 , 21.8)** | **756 (649 , 863.1)** | **334 (277 , 393)** | **18.3 (16.3 , 20.4)** | **667.9 (558.2 , 779.4)** | **-11.7 (-27.6 , 6.4)** |
| **Cook Islands** | **110 (93 , 130)** | **21.8 (18.7 , 24.9)** | **852.9 (723.8 , 1005.1)** | **153 (126 , 185)** | **21.1 (18.7 , 23.7)** | **607.6 (494.8 , 736.2)** | **-28.8 (-43 , -10.9)** |
| **Micronesia (Federated States of)** | **480 (354 , 636)** | **19.8 (16.8 , 23)** | **954.5 (714.3 , 1255.7)** | **745 (470 , 1060)** | **21 (17.4 , 25.1)** | **945.4 (620.6 , 1315.4)** | **-1 (-33.8 , 36.7)** |
| **Fiji** | **2003 (1551 , 2534)** | **13.6 (11.5 , 15.9)** | **497.7 (393 , 615.5)** | **2815 (2081 , 3755)** | **11.3 (9.7 , 13)** | **349.2 (263.1 , 457.8)** | **-29.8 (-47.9 , -4.4)** |
| **Guam** | **612 (507 , 726)** | **23 (20.1 , 26.2)** | **745.2 (615.4 , 883.3)** | **1199 (959 , 1468)** | **22.2 (19.9 , 24.8)** | **616.8 (495.3 , 752.6)** | **-17.2 (-34.5 , 6.4)** |
| **Kiribati** | **635 (478 , 802)** | **23.6 (19.6 , 27.4)** | **1504.4 (1149.7 , 1872.6)** | **1087 (786 , 1438)** | **24.2 (20.5 , 28.1)** | **1375.5 (1020.3 , 1769.2)** | **-8.6 (-31.4 , 18.6)** |
| **Marshall Islands** | **122 (86 , 172)** | **14.2 (11 , 18.2)** | **702.8 (496.8 , 990.5)** | **257 (168 , 372)** | **14.2 (11.5 , 17.6)** | **668.2 (452.1 , 958.1)** | **-4.9 (-26.4 , 25)** |
| **Nauru** | **51 (36 , 69)** | **19.2 (16.2 , 22.3)** | **1163 (839.7 , 1526.2)** | **53 (36 , 71)** | **18 (14.7 , 21.3)** | **1043.6 (733.8 , 1365.7)** | **-10.3 (-29.4 , 14)** |
| **Niue** | **14 (12 , 18)** | **19.2 (16.6 , 22.4)** | **686.5 (553.6 , 852.3)** | **14 (11 , 17)** | **19 (16.5 , 22)** | **619.7 (486.2 , 767.8)** | **-9.7 (-32.4 , 17.2)** |
| **Northern Mariana Islands** | **244 (200 , 304)** | **21.9 (19.1 , 24.9)** | **1242.4 (1058.8 , 1501.9)** | **545 (447 , 647)** | **24.1 (21.5 , 27)** | **946.7 (791.9 , 1097.3)** | **-23.8 (-36.8 , -11)** |
| **Palau** | **99 (75 , 131)** | **18.3 (15.5 , 21.2)** | **952.5 (729.6 , 1248.7)** | **190 (145 , 249)** | **18.3 (15.8 , 21.2)** | **797.7 (619.2 , 1031.3)** | **-16.3 (-39 , 12.6)** |
| **Papua New Guinea** | **9918 (7069 , 13443)** | **13.8 (11.2 , 16.9)** | **486.1 (351.3 , 655.4)** | **24436 (16764 , 34486)** | **12.2 (9.9 , 14.7)** | **470.4 (330.8 , 657.5)** | **-3.2 (-29.4 , 30.4)** |
| **Samoa** | **534 (425 , 656)** | **16.9 (15 , 18.7)** | **583.7 (468.7 , 712.3)** | **761 (607 , 969)** | **16.6 (14.4 , 18.8)** | **499.4 (405.7 , 630.3)** | **-14.4 (-33.4 , 10.5)** |
| **Solomon Islands** | **1547 (977 , 2242)** | **18.5 (15.2 , 22.7)** | **1009.4 (661.5 , 1440.9)** | **3461 (2225 , 5080)** | **14.2 (11.3 , 17.4)** | **992.3 (669.1 , 1425.1)** | **-1.7 (-27.9 , 28.9)** |
| **Tokelau** | **8 (6 , 10)** | **17.9 (14.9 , 21.3)** | **610.5 (466.6 , 784.3)** | **7 (6 , 10)** | **17.7 (15 , 20.4)** | **549.3 (420.7 , 729)** | **-10 (-32.1 , 19.7)** |
| **Tonga** | **484 (385 , 595)** | **19.8 (17 , 22.8)** | **853.6 (683.1 , 1045.5)** | **609 (485 , 755)** | **18.3 (15.9 , 20.6)** | **773.1 (617.9 , 954.7)** | **-9.4 (-29.3 , 16.5)** |
| **Tuvalu** | **59 (45 , 79)** | **19 (16.3 , 22.4)** | **795.2 (608.3 , 1061.5)** | **73 (54 , 97)** | **18.9 (16.5 , 21.7)** | **682.2 (513.9 , 903.6)** | **-14.2 (-36.6 , 18)** |
| **Vanuatu** | **354 (247 , 506)** | **14.4 (11.6 , 17.9)** | **526.1 (369.7 , 740)** | **852 (601 , 1201)** | **12.2 (10 , 15)** | **480.3 (340.9 , 673.4)** | **-8.7 (-32.8 , 27.3)** |
| **North Africa and Middle East** | **1100400 (959002 , 1257193)** | **18.2 (16.7 , 19.6)** | **600.3 (521.6 , 682.7)** | **2336468 (2053920 , 2660607)** | **19 (17.7 , 20.4)** | **515.6 (454.9 , 585.4)** | **-14.1 (-27 , 3.5)** |
| **Afghanistan** | **20838 (13433 , 30775)** | **5.4 (4.2 , 6.9)** | **279.8 (182.7 , 409.6)** | **49006 (33261 , 69565)** | **6.1 (4.9 , 7.6)** | **352.4 (246.6 , 486)** | **26 (-9.8 , 72.7)** |
| **Algeria** | **49903 (39456 , 62283)** | **14 (12.2 , 15.3)** | **406.1 (324.5 , 503.3)** | **100346 (78062 , 126324)** | **14.8 (13.4 , 16.3)** | **294.6 (230.8 , 369.8)** | **-27.5 (-45.7 , -3.3)** |
| **Bahrain** | **1680 (1423 , 1963)** | **21.3 (19.1 , 23.3)** | **1024.5 (874.2 , 1180)** | **3849 (2911 , 4997)** | **15.5 (13.6 , 17.7)** | **433 (335.5 , 544.5)** | **-57.7 (-68.3 , -43.7)** |
| **Egypt** | **113608 (96636 , 129410)** | **13.8 (11.5 , 15.7)** | **353.7 (300.7 , 401.6)** | **329753 (235512 , 454247)** | **18.1 (15.7 , 20.6)** | **467.7 (335.5 , 638.2)** | **32.2 (-3.5 , 79.5)** |
| **Iran (Islamic Republic of)** | **124334 (106565 , 142235)** | **13.4 (11.9 , 15)** | **439.8 (376 , 502.7)** | **265137 (242541 , 291851)** | **14.7 (13.6 , 16.2)** | **355 (325.4 , 389)** | **-19.3 (-30.9 , 1.4)** |
| **Iraq** | **46902 (37619 , 57817)** | **16.7 (14.2 , 19.4)** | **590.8 (475.3 , 724)** | **134803 (101742 , 166346)** | **17.3 (15.9 , 18.8)** | **575.4 (442.9 , 698.8)** | **-2.6 (-27.4 , 27.1)** |
| **Jordan** | **7955 (6595 , 9557)** | **16.4 (14.7 , 18.2)** | **554.3 (461 , 660)** | **33347 (26344 , 41090)** | **19.4 (16.5 , 21.9)** | **483.1 (383.7 , 589.4)** | **-12.8 (-34.1 , 14.5)** |
| **Kuwait** | **2581 (2293 , 2877)** | **14.1 (12.7 , 15.7)** | **389.9 (347 , 435.3)** | **7198 (5681 , 8874)** | **15.1 (13.1 , 17)** | **285.6 (225.7 , 350.7)** | **-26.7 (-41.2 , -9.2)** |
| **Lebanon** | **20062 (16140 , 24772)** | **23.9 (21.5 , 26.3)** | **842 (683.5 , 1032)** | **49626 (40614 , 63924)** | **26.9 (24.7 , 29.4)** | **957.2 (781.7 , 1234.1)** | **13.7 (-13.3 , 59.9)** |
| **Libya** | **12681 (9649 , 16064)** | **19.3 (16.6 , 21.6)** | **656.2 (505 , 823.3)** | **30683 (23180 , 39023)** | **19 (16.9 , 20.7)** | **572.6 (432.7 , 724.6)** | **-12.7 (-36.9 , 24.5)** |
| **Morocco** | **64303 (48735 , 77851)** | **18.2 (15.6 , 20.8)** | **439.6 (329.7 , 530.1)** | **137467 (99894 , 176007)** | **17 (14.7 , 19)** | **405.5 (296 , 512.3)** | **-7.8 (-33.4 , 21.1)** |
| **Palestine** | **5415 (4100 , 6991)** | **15.5 (13.4 , 17.6)** | **622.2 (475.8 , 795.9)** | **14786 (12459 , 17423)** | **16.7 (15.4 , 18.1)** | **602.4 (508.8 , 707)** | **-3.2 (-28.2 , 30.6)** |
| **Oman** | **2187 (1595 , 2837)** | **11.3 (9.6 , 13)** | **303.7 (227.7 , 387.3)** | **3414 (2665 , 4446)** | **7.6 (6.5 , 8.8)** | **194.6 (158.3 , 241.2)** | **-35.9 (-52.6 , -9.7)** |
| **Qatar** | **645 (496 , 833)** | **13.4 (11.3 , 15.8)** | **565.5 (440 , 706.9)** | **3849 (2743 , 5183)** | **13.4 (11.6 , 15.2)** | **458.1 (340.9 , 595.4)** | **-19 (-43.9 , 18)** |
| **Saudi Arabia** | **14290 (10306 , 18685)** | **10.2 (8.6 , 11.9)** | **227.3 (167.6 , 292)** | **50355 (37918 , 64799)** | **10.9 (9.5 , 12.4)** | **238.7 (187.5 , 293.6)** | **5 (-25.5 , 52.8)** |
| **Sudan** | **32298 (20534 , 46254)** | **10.8 (8.1 , 13.4)** | **335.6 (215.5 , 476.3)** | **63065 (41176 , 89237)** | **11.2 (9.3 , 13.5)** | **331.2 (219.3 , 460.2)** | **-1.3 (-27.1 , 37.4)** |
| **Syrian Arab Republic** | **24623 (18085 , 32232)** | **14.3 (11.5 , 17.1)** | **444.3 (328.7 , 573.8)** | **48921 (35487 , 66014)** | **18.9 (16.6 , 21)** | **370.7 (271.9 , 490.7)** | **-16.6 (-41.8 , 23.9)** |
| **Tunisia** | **31709 (25998 , 37764)** | **26.7 (24.2 , 29.4)** | **610.1 (503.6 , 724.2)** | **74419 (53164 , 103458)** | **28.5 (26.2 , 31)** | **572.4 (410.7 , 792.8)** | **-6.2 (-36.2 , 34.3)** |
| **Turkey** | **494872 (404043 , 595664)** | **28.9 (26.2 , 31.8)** | **1283.9 (1055.2 , 1528.2)** | **839987 (663469 , 1050212)** | **31.5 (29.8 , 33.3)** | **920.1 (726.2 , 1148.8)** | **-28.3 (-47 , -4.2)** |
| **United Arab Emirates** | **2983 (2149 , 3875)** | **11.8 (9.7 , 13.9)** | **618.9 (447.9 , 780.9)** | **28658 (19907 , 40146)** | **13.6 (11.7 , 15.5)** | **572.3 (438.5 , 727.4)** | **-7.5 (-33.9 , 36.9)** |
| **Yemen** | **25791 (18431 , 36031)** | **15.8 (12.7 , 18.5)** | **483.8 (348.9 , 669.8)** | **65426 (48048 , 88756)** | **15.7 (13.8 , 18)** | **468 (346.7 , 629.3)** | **-3.3 (-29.1 , 35.7)** |
| **South Asia** | **2576896 (2252272 , 2875777)** | **14.5 (12.9 , 16.2)** | **428.5 (371.7 , 478.1)** | **4759026 (4062951 , 5602234)** | **12.8 (11.4 , 14.3)** | **326.4 (279.3 , 382.2)** | **-23.8 (-35.9 , -7.9)** |
| **Bangladesh** | **271274 (224212 , 316479)** | **14.1 (11.7 , 16.5)** | **562.5 (464 , 653.6)** | **422635 (304419 , 582733)** | **13.6 (11.7 , 15.6)** | **317.1 (228.8 , 436.1)** | **-43.6 (-59.4 , -23.4)** |
| **Bhutan** | **735 (522 , 983)** | **9.5 (7.5 , 12)** | **273.3 (197.3 , 358.6)** | **1294 (951 , 1678)** | **9.8 (8.2 , 11.5)** | **228.1 (169.5 , 292.6)** | **-16.5 (-42.5 , 21.1)** |
| **India** | **1822657 (1560362 , 2072868)** | **13.9 (12.2 , 15.7)** | **374.2 (318.2 , 423.6)** | **3462910 (2833733 , 4206027)** | **12.7 (10.9 , 14.5)** | **293.2 (241.3 , 355)** | **-21.6 (-37.5 , -1.4)** |
| **Nepal** | **46970 (35768 , 59934)** | **13.5 (10.7 , 16.5)** | **468.2 (358.8 , 594.7)** | **79926 (60845 , 98062)** | **12.7 (11.3 , 14.3)** | **356.2 (272.7 , 434.6)** | **-23.9 (-44.5 , 0.7)** |
| **Pakistan** | **435260 (373821 , 497080)** | **18 (15.8 , 20.2)** | **744.8 (638.7 , 853)** | **792261 (610931 , 1037609)** | **12.6 (10.2 , 15.3)** | **669.3 (513.8 , 868.5)** | **-10.1 (-32.2 , 18.5)** |
| **Southern Sub-Saharan Africa** | **244249 (210020 , 289892)** | **22.2 (20.1 , 25.1)** | **838.3 (721.6 , 998.5)** | **321174 (287410 , 358475)** | **15.7 (14.2 , 17)** | **543.5 (487.1 , 605.2)** | **-35.2 (-43.6 , -25.7)** |
| **Botswana** | **3994 (2987 , 5082)** | **18.1 (15.9 , 20.6)** | **660.1 (501.6 , 831.6)** | **9564 (6791 , 12672)** | **15.3 (13.2 , 17.2)** | **651.7 (473.7 , 839.8)** | **-1.3 (-29.8 , 32.7)** |
| **Lesotho** | **5302 (4110 , 6725)** | **17.4 (14.9 , 20.5)** | **516.1 (401.7 , 651.7)** | **10317 (7777 , 13714)** | **15.7 (13.5 , 18.1)** | **761.4 (576.8 , 1010.9)** | **47.5 (4.4 , 106.8)** |
| **Namibia** | **2293 (1750 , 2854)** | **12.6 (10.5 , 15)** | **311.9 (239.7 , 387.2)** | **4083 (3087 , 5308)** | **8.8 (7.4 , 10.3)** | **291.9 (224.4 , 377.4)** | **-6.4 (-28 , 26.3)** |
| **South Africa** | **201013 (170749 , 243943)** | **24 (21.5 , 27.2)** | **907.1 (767.5 , 1110.8)** | **250523 (220439 , 285576)** | **17.1 (15.5 , 18.6)** | **533.5 (471.4 , 605.6)** | **-41.2 (-49.8 , -31.6)** |
| **Eswatini** | **1307 (969 , 1716)** | **10.3 (8.3 , 12.7)** | **433.8 (326.4 , 561.9)** | **2105 (1454 , 2904)** | **7 (5.6 , 8.6)** | **354.7 (247.5 , 484.1)** | **-18.2 (-40.8 , 15)** |
| **Zimbabwe** | **30340 (25868 , 35476)** | **17.2 (15.1 , 19.5)** | **719.2 (612.8 , 838.1)** | **44582 (34603 , 54650)** | **11.9 (10.1 , 13.6)** | **615.3 (484.8 , 746.6)** | **-14.4 (-32.4 , 5.3)** |
| **Western Sub-Saharan Africa** | **145525 (120283 , 173973)** | **5.5 (4.8 , 6.2)** | **159.6 (132.5 , 189.2)** | **294397 (239268 , 358661)** | **4.8 (4.1 , 5.4)** | **152.2 (125.6 , 183.5)** | **-4.6 (-19.4 , 13.3)** |
| **Benin** | **5127 (4145 , 6292)** | **7.5 (6.3 , 8.8)** | **256.4 (207.8 , 311.7)** | **9113 (6714 , 12149)** | **5.3 (4.6 , 6.1)** | **187.7 (141.2 , 246.1)** | **-26.8 (-44.4 , -3.9)** |
| **Burkina Faso** | **7060 (5395 , 9140)** | **5 (4.1 , 6.1)** | **151.8 (117.1 , 196.2)** | **13549 (10127 , 17429)** | **3.9 (3.3 , 4.6)** | **139.2 (105.9 , 178.6)** | **-8.3 (-28.6 , 18.1)** |
| **Cameroon** | **11736 (9237 , 14648)** | **7.8 (6.7 , 8.9)** | **246.8 (193.7 , 308)** | **30386 (21792 , 41756)** | **6.7 (5.7 , 7.7)** | **243.8 (177.7 , 330)** | **-1.2 (-30.2 , 37.6)** |
| **Cabo Verde** | **559 (483 , 656)** | **8.6 (7.5 , 9.8)** | **253.9 (218.8 , 298.7)** | **1422 (1159 , 1706)** | **8.4 (7.3 , 9.5)** | **332.9 (269.6 , 398.2)** | **31.1 (8.5 , 60)** |
| **Chad** | **5844 (4425 , 7527)** | **7.1 (5.6 , 8.8)** | **204 (154.6 , 261.2)** | **12938 (9423 , 17418)** | **6.2 (5 , 7.5)** | **228.8 (168.6 , 308.6)** | **12.1 (-16.5 , 49.4)** |
| **Côte d’Ivoire** | **13381 (10095 , 17018)** | **8.3 (7.2 , 9.7)** | **301 (229.3 , 378.7)** | **35319 (25818 , 46468)** | **9.4 (8.3 , 10.6)** | **309.1 (229.9 , 401.6)** | **2.7 (-23.8 , 35.1)** |
| **Gambia** | **1055 (693 , 1524)** | **9.5 (6.6 , 12.4)** | **278.3 (188.2 , 388.7)** | **2612 (1700 , 3753)** | **7.6 (5.3 , 10)** | **260.8 (174.4 , 364.7)** | **-6.3 (-34.5 , 31.7)** |
| **Ghana** | **10380 (7971 , 13202)** | **4.1 (3.5 , 4.7)** | **160.6 (124.5 , 200.8)** | **21910 (16768 , 27651)** | **3.8 (3.2 , 4.4)** | **133.8 (104.1 , 167.9)** | **-16.7 (-37 , 8.9)** |
| **Guinea** | **10654 (8013 , 13722)** | **7.5 (5.9 , 9.2)** | **311.1 (233.4 , 399.3)** | **22494 (15851 , 30594)** | **8.5 (6.7 , 10.5)** | **394.8 (281.7 , 529.3)** | **26.9 (-5.1 , 68.5)** |
| **Guinea-Bissau** | **1064 (698 , 1453)** | **5.4 (4.4 , 6.6)** | **248 (165.4 , 336.1)** | **1423 (999 , 1937)** | **4.3 (3.6 , 5.2)** | **184 (130.6 , 246.9)** | **-25.8 (-46.4 , 3.2)** |
| **Liberia** | **2629 (2030 , 3303)** | **7.4 (6 , 8.9)** | **227.7 (176.3 , 283.7)** | **3656 (2534 , 5072)** | **5.7 (4.8 , 6.7)** | **174.6 (122.9 , 239.5)** | **-23.3 (-45.7 , 5.4)** |
| **Mali** | **7283 (5602 , 9278)** | **4.8 (3.8 , 5.9)** | **167.5 (130 , 212.1)** | **18906 (13467 , 25399)** | **6.3 (5 , 7.6)** | **215.1 (154.3 , 286.9)** | **28.4 (-3.5 , 70.9)** |
| **Mauritania** | **2961 (2344 , 3672)** | **8.6 (7.4 , 10.1)** | **281.6 (224 , 346.3)** | **4643 (3153 , 6341)** | **8.3 (7 , 9.8)** | **214.6 (148.8 , 288.3)** | **-23.8 (-48.9 , 11)** |
| **Niger** | **2567 (1824 , 3458)** | **2.8 (2 , 3.5)** | **86.1 (61.3 , 115)** | **8155 (5505 , 11431)** | **3.4 (2.7 , 4.1)** | **102.2 (70 , 142.4)** | **18.6 (-11.4 , 59.5)** |
| **Nigeria** | **42903 (30153 , 56761)** | **3.9 (3.1 , 4.8)** | **95.3 (67.5 , 124.9)** | **69290 (50643 , 94463)** | **2.8 (2.1 , 3.5)** | **80.6 (59.9 , 108.8)** | **-15.4 (-41.6 , 24.9)** |
| **Sao Tome and Principe** | **107 (82 , 134)** | **4.4 (3.6 , 5.3)** | **159.8 (123.5 , 199)** | **259 (200 , 326)** | **5.8 (4.9 , 6.8)** | **245.2 (193.3 , 303.1)** | **53.4 (14.9 , 106.1)** |
| **Senegal** | **9165 (6997 , 11499)** | **8.4 (7 , 9.9)** | **271.1 (208.6 , 337.4)** | **17400 (12830 , 22759)** | **7.3 (6.4 , 8.4)** | **224.9 (168.3 , 292.4)** | **-17.1 (-38.7 , 14.7)** |
| **Sierra Leone** | **6678 (5140 , 8480)** | **11.5 (9.5 , 13.7)** | **342 (264.3 , 433.1)** | **10067 (7157 , 13738)** | **8.2 (6.9 , 9.7)** | **270.2 (194.5 , 363.5)** | **-21 (-42.7 , 7.3)** |
| **Togo** | **4368 (3435 , 5465)** | **9.4 (7.6 , 11.3)** | **343.1 (273.2 , 424.4)** | **10851 (7959 , 14562)** | **8.9 (7.6 , 10.3)** | **283.8 (211.5 , 377.2)** | **-17.3 (-38.4 , 12.8)** |
| **Eastern Sub-Saharan Africa** | **211976 (170862 , 255913)** | **5.6 (4.4 , 7.2)** | **269 (219.6 , 322.1)** | **379002 (302179 , 476721)** | **5.7 (4.9 , 6.7)** | **222.9 (180.7 , 274.9)** | **-17.2 (-28.5 , -3.5)** |
| **Burundi** | **10646 (7888 , 13595)** | **8.4 (6.5 , 10.4)** | **434.5 (324.2 , 548.4)** | **11423 (7866 , 16041)** | **6 (5 , 7.3)** | **227.8 (160.5 , 311.4)** | **-47.6 (-62.4 , -24.9)** |
| **Comoros** | **709 (358 , 972)** | **8.5 (6.8 , 10.4)** | **314.8 (166.8 , 427.4)** | **1161 (840 , 1571)** | **7.3 (6.1 , 8.6)** | **234.2 (171.8 , 311.7)** | **-25.6 (-48.8 , 38.9)** |
| **Djibouti** | **607 (402 , 874)** | **8.6 (6.7 , 10.6)** | **390.8 (271.9 , 550.8)** | **2832 (1850 , 4313)** | **11.1 (8.9 , 13.8)** | **439.8 (301.3 , 656.8)** | **12.6 (-18.5 , 59)** |
| **Eritrea** | **2771 (1660 , 3740)** | **5.3 (3.4 , 7)** | **225.2 (132.8 , 299.7)** | **5737 (3965 , 7708)** | **4.4 (3.4 , 5.4)** | **177.6 (124.9 , 233.7)** | **-21.1 (-43.8 , 20.4)** |
| **Ethiopia** | **29700 (20176 , 43032)** | **2.4 (1.6 , 3.7)** | **139 (93.5 , 200.9)** | **29693 (21166 , 40264)** | **2.1 (1.6 , 2.8)** | **72.2 (51.2 , 98)** | **-48 (-65.5 , -25.6)** |
| **Kenya** | **16518 (12872 , 20779)** | **7 (5.7 , 8.5)** | **194.7 (152.6 , 243.3)** | **49857 (39609 , 61941)** | **6.7 (5.6 , 7.8)** | **214.3 (172.6 , 262.6)** | **10 (-10.1 , 37.6)** |
| **Madagascar** | **15076 (11274 , 19156)** | **6.7 (5.1 , 8.5)** | **277.1 (208.1 , 351.3)** | **18415 (13040 , 26067)** | **4.7 (3.8 , 5.8)** | **150.1 (108.5 , 207.2)** | **-45.8 (-61.1 , -27.4)** |
| **Malawi** | **12325 (9681 , 15897)** | **4.5 (3.3 , 6.1)** | **307.8 (245.9 , 390.1)** | **24449 (17755 , 33339)** | **6.6 (5.2 , 8.2)** | **325.5 (242.7 , 434.1)** | **5.8 (-18.7 , 36.4)** |
| **Mozambique** | **12746 (9575 , 16458)** | **5.6 (4.4 , 6.8)** | **202.7 (156.6 , 256.4)** | **27868 (20471 , 37794)** | **5.6 (4.5 , 6.9)** | **243.4 (184.3 , 318.3)** | **20.1 (-12.2 , 63)** |
| **Rwanda** | **15851 (11684 , 20329)** | **9.3 (7.2 , 11.7)** | **523.7 (392.7 , 662.8)** | **24552 (17951 , 33224)** | **10.4 (8.6 , 12.5)** | **403.5 (304.9 , 530.8)** | **-22.9 (-43.3 , 12.5)** |
| **Somalia** | **8615 (5682 , 12082)** | **6.4 (4.7 , 8.1)** | **305.6 (207.3 , 421.9)** | **16236 (10199 , 23399)** | **5.3 (4.1 , 6.8)** | **222.7 (139.5 , 316.9)** | **-27.1 (-46.7 , 3.5)** |
| **South Sudan** | **7822 (5419 , 10756)** | **7.3 (5.4 , 9.3)** | **321 (224.2 , 438.8)** | **9038 (6151 , 12903)** | **6.3 (4.9 , 7.7)** | **230.1 (159.1 , 323.1)** | **-28.3 (-49 , -0.2)** |
| **United Republic of Tanzania** | **51681 (38373 , 69894)** | **9.9 (7.7 , 12.6)** | **447.1 (337.6 , 599.3)** | **97706 (69278 , 137723)** | **9.1 (7.3 , 11.2)** | **378.7 (275 , 524.6)** | **-15.3 (-34 , 5.9)** |
| **Uganda** | **15683 (11769 , 20236)** | **5.5 (4.4 , 6.8)** | **232.1 (176.2 , 296.4)** | **37089 (28154 , 47736)** | **5 (4.1 , 6.1)** | **250.4 (193 , 319.3)** | **7.9 (-16.7 , 38.7)** |
| **Zambia** | **11070 (8625 , 13813)** | **6.2 (4.7 , 7.8)** | **382.1 (300.7 , 473.2)** | **22644 (16682 , 29294)** | **6.5 (5.5 , 7.6)** | **335.1 (252.6 , 427)** | **-12.3 (-33.2 , 14.9)** |
| **Central Sub-Saharan Africa** | **83105 (59646 , 129035)** | **8.5 (6.5 , 11.5)** | **336.2 (244.2 , 518.7)** | **140195 (99531 , 198136)** | **7.3 (6 , 9.4)** | **242 (172.7 , 340.9)** | **-28 (-45.2 , -6.2)** |
| **Angola** | **19220 (14042 , 25643)** | **10.6 (8.1 , 12.7)** | **446.9 (326.9 , 582)** | **43595 (34175 , 56349)** | **10 (8.8 , 11.2)** | **356.4 (285.3 , 449.4)** | **-20.2 (-43.2 , 12.1)** |
| **Central African Republic** | **5257 (3670 , 7510)** | **9 (7 , 11.6)** | **398.6 (283.5 , 558.5)** | **6842 (4473 , 10415)** | **7.2 (5.6 , 9.4)** | **273.2 (184.3 , 410)** | **-31.5 (-48.9 , -11.5)** |
| **Congo** | **4697 (3284 , 6342)** | **8.7 (6.9 , 10.7)** | **412.1 (296.3 , 547.4)** | **8586 (6446 , 11173)** | **8.1 (6.9 , 9.4)** | **308.9 (241.1 , 397.1)** | **-25.1 (-44.2 , 1.5)** |
| **Democratic Republic of the Congo** | **51003 (33325 , 88973)** | **7.8 (5.6 , 11.6)** | **298.5 (195.4 , 518.6)** | **75904 (47002 , 121259)** | **6.2 (4.6 , 9.2)** | **195.5 (121.6 , 310.9)** | **-34.5 (-51.7 , -10.6)** |
| **Equatorial Guinea** | **715 (450 , 1096)** | **8.9 (6.4 , 12.1)** | **329.3 (207.2 , 497.9)** | **1348 (886 , 2021)** | **7.3 (5.9 , 9.1)** | **272.1 (183.7 , 395.4)** | **-17.4 (-51.9 , 40.7)** |
| **Gabon** | **2213 (1557 , 3021)** | **9.2 (7.6 , 11.3)** | **372 (264.1 , 504)** | **3921 (2827 , 5136)** | **9.5 (8 , 11.1)** | **349.3 (255.3 , 454.2)** | **-6.1 (-30.5 , 25.6)** |
